# Neural Functions Play Different Roles in Triple Negative Breast Cancer (TNBC) and non-TNBC

**DOI:** 10.1038/s41598-020-60030-5

**Published:** 2020-02-20

**Authors:** Renbo Tan, Haoyang Li, Zhenyu Huang, Yi Zhou, Mingxin Tao, Xin Gao, Ying Xu

**Affiliations:** 1 School of Life Sciences, Tsinghua University, Beijing, 100084 China; 2 Cancer Systems Biology Center, The China-Japan Union Hospital of Jilin University, Changchun, 130033 China; 3 College of Computer Science and Technology, Jilin University, Changchun, 130012 China; 4 Computational Bioscience Research Center, King Abdullah University of Science and Technology, Thuwal, 23955 Saudi Arabia; 5 Computational Systems Biology Lab, Department of Biochemistry and Molecular Biology and Institute of Bioinformatics, University of Georgia, Athens, 30602 USA

**Keywords:** Breast cancer, Breast cancer

## Abstract

Triple negative breast cancer (TNBC) represents the most malignant subtype of breast cancer, and yet our understanding about its unique biology remains elusive. We have conducted a comparative computational analysis of transcriptomic data of TNBC and non-TNBC (NTNBC) tissue samples from the TCGA database, focused on genes involved in neural functions. Our main discoveries are: (1) while both subtypes involve neural functions, TNBC has substantially more up-regulated neural genes than NTNBC, suggesting that TNBC is more complex than NTNBC; (2) non-neural functions related to cell-microenvironment interactions and intracellular damage processing are key inducers of the neural genes in both TNBC and NTNBC, but the inducer-responder relationships are different in the two cancer subtypes; (3) key neural functions such as neural crest formation are predicted to enhance adaptive immunity in TNBC while glia development, along with a few other neural functions, induce both innate and adaptive immunity in NTNBC. These results reveal key differences in the biology between the two cancer subtypes, particularly in terms of the roles that neural functions play. Our findings may open new doors for further investigation of the distinct biology of TNBC vs. NTNBC.

## Introduction

Triple negative breast cancer (TNBC) is a most malignant subtype of breast cancer as it is more aggressive and easier to metastasize compared to the other subtypes^1,2^. The disease counts for about 15–20% of all breast cancer cases. African Americans and young women in their 30’s – 40’s are known to have un-proportionally elevated occurrence rates of TNBCs compared to other races and age groups in the USA^3^. TNBC gets its name from its characteristic repression of gene expressions of its estrogen receptor (ER), progesterone receptor (PR), and human epidermal growth factor receptor 2 (HER2) while other breast cancers generally have at least one of these receptors over-expressed. Hence, drugs targeted at any of these receptors are not useful to TNBC patients. Chemotherapy represents the predominant approach to treating the disease. Published data suggest that the lack of expressions by ER, PR and HER2 may not necessarily be the reason for the deadly nature of this disease. While substantial efforts have been invested into studies of the TNBC biology and treatment, the overall understanding about the unique biology of the disease remains limited.

Like angiogenesis and lymphogenesis in cancer, the term “neo-neurogenesis” has been coined by cancer researchers to study functional roles played by the neural system in cancer^4,5^. Functional involvement of neural processes in cancer has been studied by numerous authors, such as that (1) perineural invasion has been observed in multiple cancer types^6,7^; (2) cancer cells surrounding nerves tend to be more resistant to apoptosis^8,9^; and (3) denervation has been found to be effective in slowing down cancer progression^10–12^. Furthermore, cross-talks have been observed between cancer cells and nerves, including neurites growing towards cancer cells and cancer cells invading nerves^13,14^. It has been shown that sympathetic nerves function at the onset of the prostate cancer; and parasympathetic nerves are involved in cancer metastasis in both xenograft and transgenic prostate cancer mouse models^15^. However, the detailed functional roles by the neural systems in cancer formation and development, particularly in breast cancer, remain elusive.

Nervous system is one of the most sophisticated systems in human body, which is divided into the afferent and efferent nerves. Previous studies have been largely focused on roles played by the sympathetic and parasympathetic nerves in cancer development^16–18^, where the two together make the autonomic nerves, a subtype of the efferent nerve, while the functional roles by other components of the nervous system are largely unknown in cancer formation. Development of the nervous system can be partitioned into the following four stages from early to late: (a) neural fate commitment, neural plate formation (e.g., continuous proliferation of neural precursors) and neural tube closure^19^; (b) neuron differentiation and migration^20^; (c) axon growth, dendrite pruning and glial differentiation^21^; and (d) synapse formation, neurotransmitter production and synapse apoptosis^22^.

We present a computational study of transcriptomic data of TNBCs and non-TNBCs (or NTNBCs) in the TCGA database focusing on the distinct functional roles played by the neural system throughout the development of the two subtypes of breast cancers^23,24^. Our main findings are: (i) numerous neural genes are found up-regulated in both TNBC and NTNBC, but TNBCs have considerably more such genes than in NTNBC, indicating that TNBCs have higher levels of neural functions; (ii) neural genes are transcriptionally correlated with genes involved in cell-microenvironment interactions and intracellular damage response processes in both TNBC and NTNBC but with different co-expression patterns between the subtypes; (iii) substantially more neural functions are utilized in TNBC than in NTNBC; (iv) genes involved in neural crest development and neurotransmitter secretion are predicted to regulate adaptive immunity and cell cycle only in TNBC, while genes relevant to glia development seem to promote both innate and adaptive immunity, as well as cell cycle regulation in NTNBC.

## Results

### Identification of neural genes in cancer

The following is conducted to identify a maximal set of neural genes. 18,020 human genes with functional annotations were downloaded from the Gene Ontology Database. Genes labelled as neural function related are kept, giving rise to 4,115 neural genes, which fall into 1,225 GO biological processes and are known to be expressed in some neural structures or neural development processes in human.

### TNBC and NTNBC use different neural functions

Differential expression analyses are carried out on the 4,039 neural genes between TNBC and matching controls; and similar are done between NTNBC and corresponding controls. Supplementary Table S1 shows the numbers of up-regulated neural genes in TNBC and NTNBC, respectively.

We have examined the up-regulated non-neural genes that exhibit strong co-expressions with the above neural genes in TNBC and NTNBC, respectively. Supplementary Table S2 summarizes the numbers of the neural genes correlated with the expressed non-neural genes, or simply NCN genes across different stages of TNBC and NTNBC, respectively. We note from the table that TNBC has considerably more NCN genes than NTNBC, suggesting that neural genes play more significant roles in TNBC compared to NTNBCs.

Pathway enrichment analyses are carried out over the NCN genes in TNBC and NTNBC, respectively, with each enriched pathway termed as a neural pathway. Overall 589 and 220 distinct neural pathways are enriched in TNBC and NTNBC, respectively. These pathways were classified into eight functional categories as detailed in Table 1.

**Table 1:** Enriched neural pathways by NCN of TNBC and NTNBC.

Neural pathways	TNBC	NTNBC
neurotransmitter secretion	29	0
neural crest development	10	0
axon growth and dendrite pruning	61	6
synapse formation	142	12
neuron projection and apoptosis	85	37
neural structure formation in CNS	104	69
neuron differentiation	140	84
development of glia	18	12
Total number	589	220

From the table, we see: (1) more neural pathways are involved in TNBC than NTNBC in each of the eight categories; and (2) the level of difference in four categories, namely neurotransmitter secretion, neural crest development, axon growth and dendrite pruning, and synapse formation, is particularly prominent, where these four categories of neural functions are generally used in late developmental stages of the neural system.

**Figure 1:**
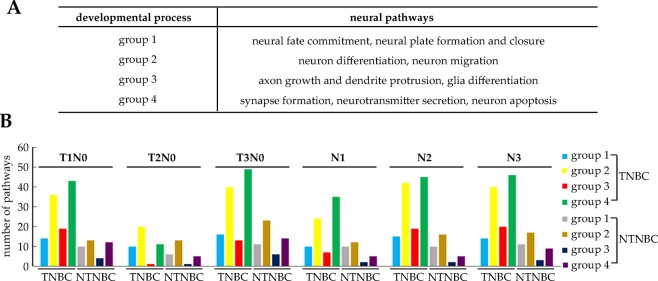
The numbers of neural pathways of different groups in distinct developmental stages in TNBC and NTNBC, respectively. (**A**) Definition of four groups of neural functions. (**B**) The number of neural pathways in each group in TNBC vs. NTNBC, where the color definition is given on the right side of the figure.

We have then classified all these neural pathways into four groups according to the relative order of their occurrence during the developmental process of the neural system, as shown in Fig. 1(A). Specifically, group 1 consists of pathways relevant to neural fate commitment, neural plate formation and closure; group 2 is composed of pathways related to neuron differentiation and migration; axon growth, dendrite protrusion, and glia differentiation make up group 3; and group 4 is largely made of synapse formation, neurotransmitter secretion and neuron apoptosis pathways^22^. Figure 1(B) shows the number of the pathways in each group used by cancer samples at different stages of TNBC and NTNBC, respectively.

Overall, substantially more neural pathways in every group are involved in TNBC than in NTNBC, ranging from 1.07 to 3.12 times more from group 1 to group 4. It is noteworthy that increasingly more neural pathways are employed in more advanced developmental groups in TNBC than in NTNBC as shown in Fig. 1(B). This is consistent with the data shown Table 1, which shows that TNBC utilizes more neural developmental pathways than NTNBC.

### Neural genes-related non-neural processes in TNBC vs. NTNBC

**Figure 2:**
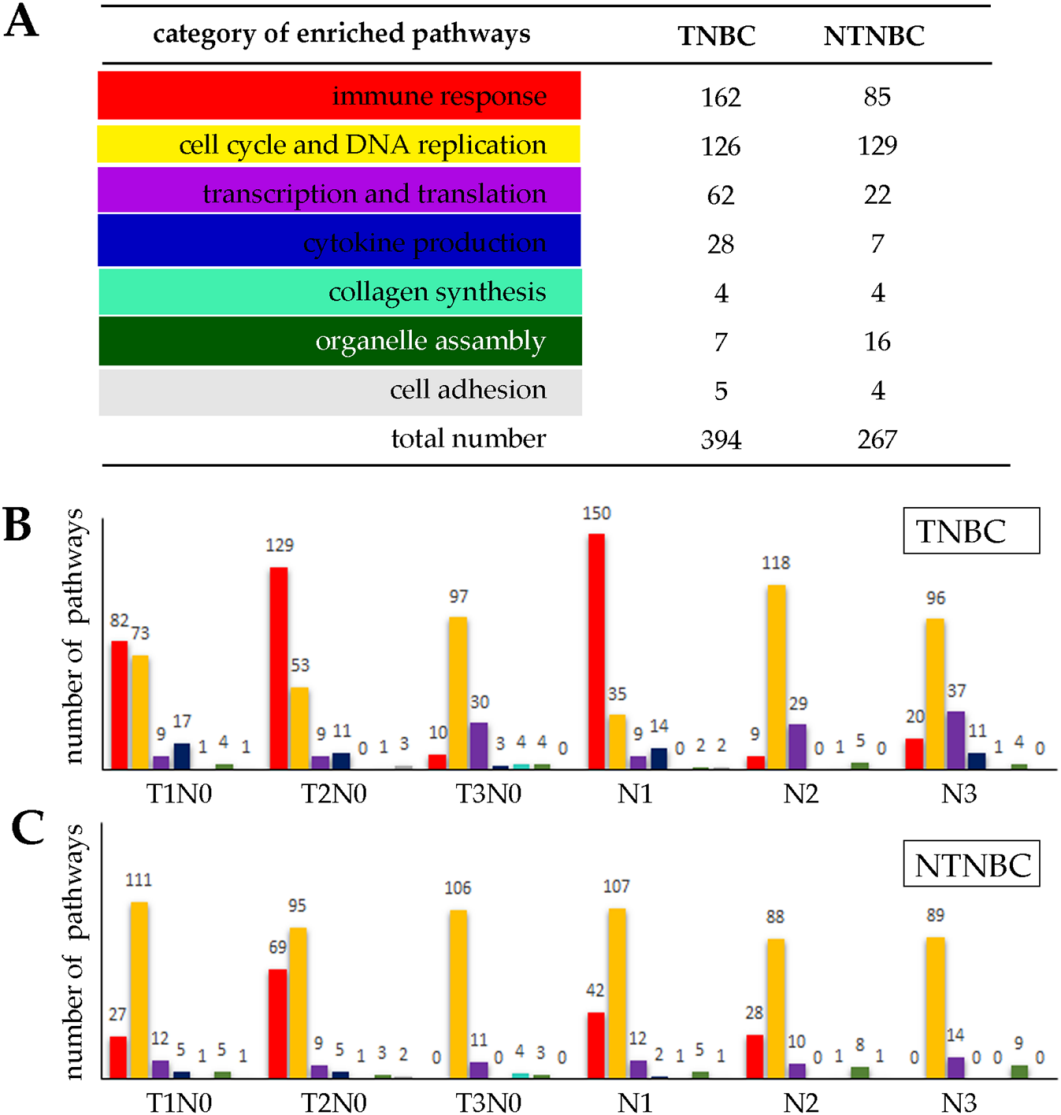
The numbers and distributions of enriched NNCN pathways in TNBC and NTBC, respectively. (**A**) The first column is for pathway category; the second and the third are for the numbers of enriched pathways in TNBC and NTNBC, respectively. (**B**) The numbers of each category across different stages of TNBC, where red represents “immune response”; yellow for “cell cycle and DNA replication”; purple for “transcription and translation”; dark blue for “cytokine production”; light blue for “collagen synthesis”; dark green for “organelle assembly”; and gray for “cell adhesion”. (**C**) The numbers of each category across different stages of NTNBC.

To further our understanding of the functions played by neural genes in breast cancer, we have examined the functions of the up-regulated non-neural genes that are positively correlated with the expressed neural genes, referred to as NNCN. 4,233 and 598 such genes are identified in TNBC and NTNBC, respectively. Pathway enrichment analyses are conducted over the NNCN genes in TNBC and NTNBC (p-value < 0.05), respectively, using the same selection criteria for NCN genes to select the NNCN genes. 394 and 267 pathways are enriched in TNBC and NTNBC, respectively (Fig. 2(A)). We then binned the pathways into eight functional categories: immune response related, cell cycle and DNA replication, transcription and translation, cytokine production, collagen synthesis, organelle assembly, and cell adhesion related pathways. Figure 2(B,C) show the number of pathways in each category across the six stages of TNBC and NTNBC, respectively.

We note: (1) throughout the entire development, the predominant NNCN pathways enriched in NTNBC are cell cycle and DNA replication pathways; as a comparison, immune response pathways represent the mostly used in TNBC with cell cycle and DNA replication pathways as the second; (2) interestingly, more organelle assembly pathways are employed in NTNBC than in TNBC; and (3) comparable numbers of collagen synthesis as well as cell adhesion pathways are utilized in TNBC and NTNBC.

Our interpretation of these data is: (i) the extents of neural gene-related cell cycle & DNA replication pathways are comparable in TNBC and NTNBC, suggesting that the neural system may play similar roles in cell division of TNBC and NTNBC; (ii) immune responses play more significant roles in TNBC than in NTNBC, suggesting higher levels of tissue damages in TNBC than in NTNBC, which is consistent with the observed stronger associations between neural genes and cytokine production in TNBC; (iii) NTNBC shows stronger association with organelle assembly, suggesting that TNBC has reduced level of activities in maintaining organelle structures, which is consistent with the literature regarding more malignant cancer^25^; and (iv) overall, involvement by neural genes in non-neural functions tend to be considerably more extensive in TNBC than in NTNBC, suggesting that higher levels of challenges faced by TNBCs and hence requiring more involvement by the neural genes, knowing that they tend to lead the way in various developmental processes^26,27^.

### Functional relations between neural and non-neural functions in TNBC vs. NTNBC

We have examined co-expressions between neural genes and non-neural genes at each stage of the disease. Here we show the relevant data for T3N0 (advanced stage primary tumor without metastasis) and N1 with metastasis to one lympho-node, while data for the other stages are given in Supplementary Figs. S1–4.

**Figure 3:**
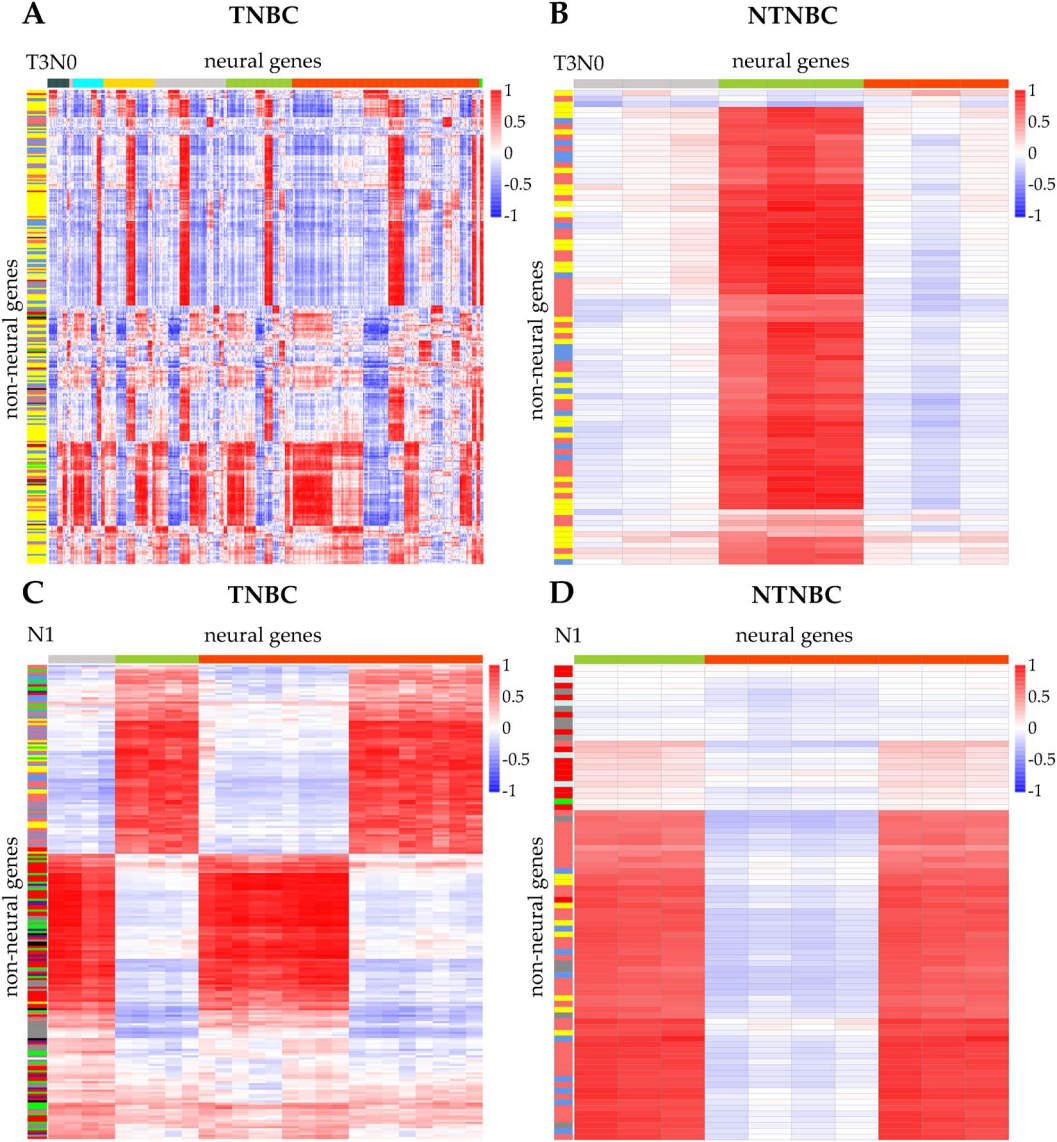
Heat-maps for correlations between non-neural genes and neural genes at stages T3N0 and N1 in TNBC and NTNBC, respectively. The horizontal and vertical axes are selected neural and non-neural genes, respectively, at T3N0 (**A**) and N1 (**C**) in TNBC; and at T3N0 (**B**) and N1 (**D**) in NTNBC, where the sequential order of the neural genes is determined as follows: dark blue for neurotransmitter secretion related genes, pink for neural crest formation related genes, light blue for axon and dendrite growth related genes, yellow for synapse formation related genes, gray for neuron projection related genes, dark green for neural structure formation in CNS related genes, orange for neuron differentiation related genes, and light green for glia development related genes. Similarly, the color scheme for the non-neural genes is defined as: red for cell adhesion; dusty blue for calcium sequestering; green for cytokine and chemokine production; gray for regulation of cell death; white for ECM synthesis; black for hemopoiesis; purple for endocytosis; yellow for DNA damage and metabolism; pink for organelle assembly; blue for cell skeleton synthesis; and brown for de-development process.

We have examined all non-neural genes found in Fig. 2 having Pearson correlation coefficients with neural genes at least 0.8 and p-value < 0.05. We note: the so selected non-neural and neural genes have the following properties: (1) the functional categories enriched by non-neural and neural genes, separately, in TNBC contain those in NTNBC at stage T3N0, except for ECM production, which is observed only in NTNBC; (2) similar is observed for stage N1 with one exception for calcium activity, which is observed only in NTNBC; and (3) from stage T3N0 to stage N1, the number of neural functions involved tend to decrease in both TNBC and NTNBC while in comparison, the associated non-neural functions tend to shift from being highly DNA-damage and metabolism related at stage T3N0 to highly cell adhesion and organelle assembly related in both TNBC and NTNBC. All these are shown in Fig. 3.

The information shown in Fig. 3 has revealed that: (1) TNBC involves considerably more interactions between neural and non-neural functions than NTNBC; (2) TNBC has considerably more complex co-expression patterns between neural and non-neural genes than NTNBC, together strongly suggesting that the complexity and hence possibly challenges in TNBC are substantially higher than in NTNBC.

We have then checked if the observed neural functions could be statistically explained by the non-neural functions, aiming to lay a foundation for further investigation of the possible causal relationships between them. We consider that a neural function is statistically explained by non-neural functions if changes in the expression levels of the neural genes can be well represented as a function of changes in the expression levels of the non-neural genes. This can be done through a regression analysis of the expressions of the neural genes against the expressions of the non-neural genes, followed by a feature-selection procedure to remove all the non-neural genes that have insignificant contributions to the regression result. Specifically, a set of neural genes is considered as explainable by a set of non-neural genes if the R^2^ value of the linear regression is at least above 2/3 = 0.67 (with p-value < 0.05) and this set of non-neural genes is minimal, i.e., any proper subset of the pathways could not reach this R^2^ level.

**Figure 4:**
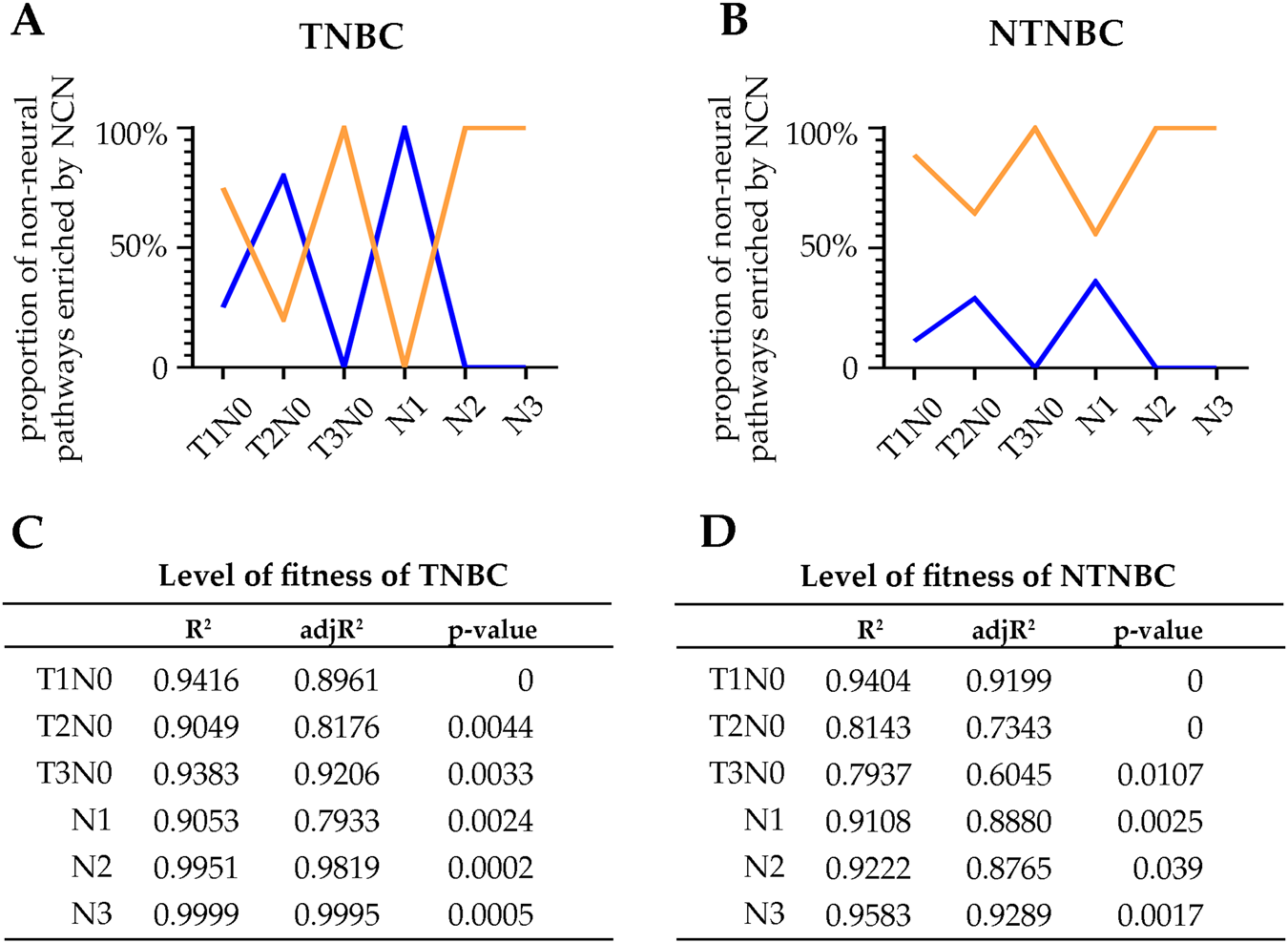
Percentages of non-neural pathways that correlate with neural genes and fall into the categories of cell-environment interaction and intracellular damage processing out of all the neural genes associated non-neural pathways in TNBC (**A**) and NTNBC (**B**) across different stages, respectively, where the y-axis denotes the fraction. The blue line represents the percentages of non-neural pathways related to cell-environment interactions and the orange line for intracellular damage-processing activities. The quality of the regression models of TNBC and NTNBC given in (**C**,**D**).

A regression analysis is conducted over samples in each stage of TNBC and NTNBC, respectively, with details given in Fig. 4. The regression results have revealed a particularly interesting and unexpected relationship between neural vs. non-neural pathways in TNBC: neural pathways predominantly correlate with pathways associated with cytokine and chemokine production, cell adhesion, calcium sequestering, hemopoiesis, and extracellular matrix production at T2N0 and N1 while they virtually all correlate with pathways associated with cell skeleton synthesis, DNA damage response and organelle assembly in T1N0, T3N0, N2 and N3. For simplicity, we refer to these two classes of pathways as cell-environment interactions and intracellular damage processing, respectively. This striking behavior is shown in Fig. 4(A). Somewhat similar but clearly weaker patterns were observed in NTNBC, as given in Fig. 4(B), namely, neural pathways mostly correlate with intracellular damage processing activities in stages T2N0 and N1 but with cell-environment interaction activities in the alternating stages. These results are consistent with the results shown in Fig. 3. In addition, these observations are consistent with a previous study showing that more immune cells are involved in TNBC compared to NTNBC^28^.

Based on these data, we predict: neural functions may be activated for different reasons in TNBC vs. NTNBC. In TNBC, the predominant reasons for neural activities are cell-environment interactions and intracellular damage processing but in a mutually exclusive manner while in NTNBC, intracellular damage processing seems be the major reason for neural pathways activation while cell-environment interactions play a minor but complementary role in activating the neural pathways.

To demonstrate the possible contribution by cell-environment interaction and intracellular damage processing activities to the induction of neural functions, a regression analysis for each neural function (Table 1) against the expressions of all the non-neural pathways at each stage of TNBC and NTNBC was carried out, respectively, again using R^2^ > 2/3 = 0.67 and p-value < 0.05 as cutoffs. The goal is to identify cell-environment interaction and intracellular damage processing related pathways that can statistically explain each category of neural pathways, hence providing statistical evidence for the possible causes for the activation of individual neural functions.

The regression results are shown in Supplementary Table S3, with the number of unique non-neural pathways belonging to “cell-environment interaction pathways” and “intracellular damage processing pathways”, respectively. More detailed information such as the R^2^ values and p-values of the regressions in TNBC and NTNBC are given in Supplementary Tables S4 and S5, respectively. Also, non-neural genes belonging to non-neural pathways, which are considered as potential explanations for specific neural functions are shown in these two Supplementary Tables.

So far we have noted: (1) non-neural pathways in TNBC are involved in majority of the neural development except for glia development, while in NTNBC, six neural functions could be statistically explained by cell-environment interaction and intracellular damage processing activities except for neurotransmitter secretion and neural-crest development; (2) in TNBC, both cell-environment interaction and intracellular damage processing activities seem to contribute to the induction of neural functions at stage T2N0; between the two, cell-environment interactions seem to have higher levels of contribution to the induction; and at stage N1, cell-environment interactions seem to play a major role in inducting the neural pathways; (3) compared to TNBC, intracellular damage processing activities seem to be the predominant inducer of the neural functions at stages T2N0 and N1 in NTNBC.

### Different neural functions promote the immune response and cell proliferation in TNBC vs. NTNBC

It has been reported that the nervous system plays essential roles in changing the microenvironment during cancer progression, where enhanced immune responses and cell cycle activities are observed^29,30^. So far, we have statistically shown that several cell-environment interaction and intracellular damage processing aim intend to find out which neural functions may induce some of the observed non-neural functions. We have specifically examined which neural functions may possibly enhance immune response and promote cell cycle and DNA replication. Similar regression analyses were conducted to those in the previous section with detailed regression data given in Supplementary Tables S6 and S7. Tables 2 and 3 summarize the predicted level of contributions to the enhanced immunity and cell proliferation by neural functions in TNBC and NTNBC across different stages, where a neural function is considered as contributing to a specific category if the difference in R^2^ is >0.01 with vs. without the neural function with p-value < 0.01.

**Table 2:** Neural functions predicted to contribute to immune responses and proliferation process in TNBC across different stages, respectively.

	T1N0	T2N0	T3N0	N1	N2	N3
innate immunity	telencephalon development; pallium development	CNS neuron differentiation; neural tube closure; telen-cephalon development	associative learning	regulation of dendrite morpho-genesis		cerebral cortex development
adaptive immunity	telencephalon development; pallium development	pallium development; neural precursor cell proliferation	associative learning; cerebral cortex development	neuron death; neural crest cell differen-tiation		
cell cycle regulation	telencephalon development	negative regulation of synaptic transmission; cerebral cortex development	forebrain neuron differentiation; neuroblast proliferation	regulation of neurotransmitter secretion; regulation of dendrite morpho-genesis		
DNA replication	regulation of synaptic transmission; telencephalon development		neuroblast proliferation; forebrain neuron differentiation	primary neural tube formation		dendritic spine development

**Table 3:** Neural functions predicted to contribute to immune responses and proliferation process across different stages in NTNBC.

	T1N0	T2N0	T3N0	N1	N2	N3
innate immunity	regulation of neuron apoptotic process; neuron fate commitment; axonal transport	neuron death; CNS neuron differentiation; pallium development		neural tube closure; pallium development; gliogenesis; cerebral cortex development	sensory perception of sound; pallium development; neuron fate commitment	
adaptive immunity	neural tube formation; negative regulation of synaptic transmission; brain development	forebrain development, CNS development; neuron death; CNS neuron differentiation		gliogenesis; neural precursor cell proliferation; neuron fate commitment; CNS development	CNS neuron differentiation; primary neural tube formation	
cell cycle regulation		regulation of nervous system development	gliogenesis; regulation of gliogenesis	pallium development		regulation of neural precursor cell proliferation; sensory perception of sound; neural tube closure; sensory perception of mechanical stimulus
DNA replication		cerebral cortex development; neuron apoptotic process	primary neural tube formation	spinal cord development		neural tube formation; pallium development

Based on the regression results, we predict: (1) neural development processes play roles in enhancing innate and adaptive immunity in both TNBC and NTNBC with the following differences: “neural crest cell differentiation” is expressed only in TNBC, while “glio-genesis” is observed only in NTNBC; (2) substantially different sets of neural functions are involved in enhancing the cell cycle and DNA replication process in TNBC vs. NTNBC, again highlighting the differences in the basic biology in the two subtypes of breast cancers; (3) among other neural functions, “regulation of neurotransmitter secretion” seems to be used to promote cell cycle in TNBC while in NTNBC “glio-genesis” and “regulation of glio-genesis” may play this role; (4) “neural crest cell differentiation” and “regulation of neurotransmitter secretion”, uniquely observed in TNBC, seems to play roles in contributing to the induction of immunity and cell proliferation; and (5) “glio-genesis” seems to play roles in inducing both the innate and the adaptive immunity, as well as cell cycle progression in NTNBC, which is supported by the data shown in Supplementary Table S3.

These predictions are supported by published studies as detailed below. (1) It is reported that thymus cannot develop properly without embryonic connective tissue ablation, which originates from neural crest^31,32^. In addition, it has been reported that neural crest is indispensable in T-cell egress from thymus^33^. (2) Neurotransmitters, such as neuropeptide Y are essential in proliferation of cultured fibroblasts and keratinocytes *in vitro*^34,35^. (3) An immunologic function of glia in CNS is to destroy pathogens and clear dead neurons^36^, which are recruited by pro-inflammation signals and act like macrophages in CNS^37,38^. (4) Schwann cells, the glia in PNS, participate in innate immunity by producing proinflammatory factors, resulting in the recruitment of immune cells, and releasing anti-inflammatory cytokines to modulate immunity activities^39^. Schwann cells can also regulate the complement cascade^40^, where major histocompatibility (MHC) classes I and II help Schwann cell recognize exogenous and endogenous antigens and present them to immune cells^41^. And (5) Schwann cells are known to be reactivated during tissue repair by stimulating proliferation of the surrounding mesenchymal precursor cells^42^.

## Discussion

**Figure 5:**
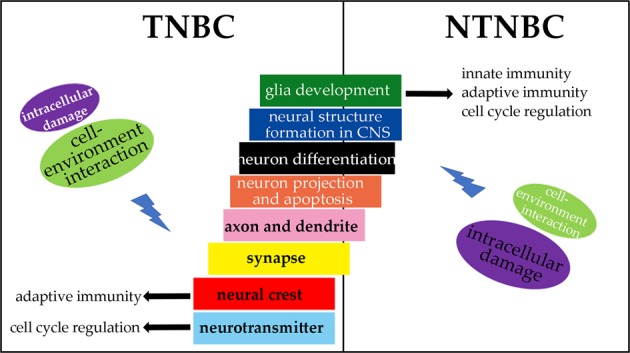
A model for cross-talks between non-neural functions and neural functions in TNBC and NTNBC, respectively, where the location of each box with respect to the middle dividing line is proportional to the numbers of relevant pathways observed in TNBC vs. NTNBC.

In this study, we have demonstrated that there are substantial differences in terms of the utilization of the neural functions in TNBC vs. NTNBC; and predicted their possible causes and functions. We have statistically shown that considerably more neural functions are involved in TNBC than NTNBC. Based on these, we have predicted that cell-environment interactions and intracellular damage processing activities represent two large classes of signals that may induce the neural activities in both TNBC and NTNBC, with TNBC having substantially more interactions between neural and non-neural functions than in NTNBC. Furthermore, we have provided evidence regarding how neural functions might have driven different immune responses in the two subtypes of breast cancer, knowing the leading roles of neural functions in organ development and tissue repair, as well as in modulating immunity response and cell proliferation^43–48^. Figure 5 summarizes the cross-talks between different neural and non-neural functions in TNBC and NTNBC.

It is noteworthy that TNBC does not represent a homogeneous class of breast cancer. Previous studies have classified TNBC into six subgroups, namely two basal-like-related subgroups (BL1 and BL2), two mesenchymal-related subgroups (M and MSL), one immunomodulatory subgroup (IM) and one luminal androgen receptor group (LAR)^49^. Published data have documented that this class of heterogeneous tumors has higher propensity for higher mutation burden, distant visceral metastasis, worse outcomes and a more aggressive presentation than the other subtypes of breast cancers^50^. These similar aggressive behaviors suggest that TNBC tumors may share some common characteristics, which are not shared by non-TNBC tumors, which forms an even larger class of breast cancers consisting of luminal A, luminal B, HER2-enriched and basal-like tumors^51^. Our study examined some of these commonalities from the perspective of neural functions, and offered explanations of how they drive the more aggressive behaviors of the TNBC tumors compared to the non-TNBC tumors.

Substantial interactions have been observed between breast cancer cells and the microenvironment via the nervous system^12,52,53^, namely: (i) breast cancer cells and local immune cells release neurotrophic factors (such as nerve growth factor, NGF) to facilitate nerve invasion^54,55^; (ii) neurotransmitter secreted by nerve sprouting can act on both breast cancer cells and stromal cells; (iii) repression of neurotrophic factors and receptors can slow down the disease progression^56^; (iv) once cancer cells penetrate the nerve endoneuria and migrate along the nerve fiber, relapse tends to happen more frequently^57^; and (v) TNBC has higher death rate than those of the other breast-cancer subtypes, one reason being its high recurrence rates, and the central nerve system (CNS) is one of the most likely location of recurrence in TNBC.

Based on these observations, we aim to elucidate how the nervous system interacts with the tumor microenvironment to facilitate tumor growth. Specifically, we are interested in deriving information towards establishing the possible causal relationships between the observed neural functions and the elevated non-neural functions, based on gene-expression data. This is done largely through regression analyses between gene-expression data associated with neural functions vs. those of non-neural functions. Statistical explainability, based on the regression results, provides the directionality of the statistical associations established via our analyses, which is further supported by published studies on each predicted causal relationship.

The tumor microenvironment is inflammatory because of the inflammatory cells and increased cytokine concentrations. Previous studies have demonstrated that sensory nerves can convey peripheral inflammation signals into the CNS, leading to the release of certain neurotransmitters and neuropeptides in autonomic nerves, triggering the production of pro-inflammatory or anti-inflammatory mediators^58^. It was inferred that sympathetic nerves recruit a large number of immune cells by secreting epinephrine and norepinephrine to promote the inflammatory responses, while parasympathetic nerves inhibit the inflammatory response by secreting acetylcholine^59^. Our data strongly suggest that neural functions participate in immune responses in both TNBC and NTNBC but there is a clear difference in terms of the detailed neural functions. We believe that neural genes, correlated with inflammatory tumor microenvironment signals could interact with central nervous system and reshape the tumor microenvironment through autonomic nerves, but the mechanisms need further investigation.

Due to the limited experimental data available, considerable questions of neural functions at the tissue level remain to be elucidated. Bioinformatics techniques provide novel angles through systematic comparative analyses of gene-expression data of cancers of different subtypes. It has been widely accepted that neural functions are indispensable in tissue repair^43,45–47^. For example, tissue repair in patients with nerve damages or spinal cord injury tends to be slow^35^. In rat corneal repair, nerve sprouting towards the wound can accelerate the wound healing process^48^. These are clearly consistent with our prediction that neural functions may participate in tissue repair. Based on this and our earlier discussion, we posit that more complex and intensive tissue repair might have been a key reason for the worse prognoses than those involving tissue repair to lesser extent. In tumor research, the study of regulating the balance between tissue damage and tissue repair by neural systems is of great interest as well as challenge, which we aim to pursue further.

Overall, our analyses suggest that the more complex and challenging microenvironments that derive the more extensive utilization of the neural system in TNBC vs. NTNBC. To the best of our knowledge, this is the first report regarding how extracellular and intracellular environments may have triggered extensive involvement of neural functions in breast cancer. Clearly, further investigation is needed in figuring out the detailed regulatory mechanisms from the intracellular and extracellular cues to the induction of the neural system.

## Methods

### Data sets

RNA-sequence data of 119 TNBC tumor and 11 control samples, 1,066 NTNBC tumor and 109 control samples, along with corresponding clinical information were downloaded from The Cancer Genome Atlas (TCGA) database https://portal.gdc.cancer.gov. Each tumor sample is classified into one of the following stages based on the clinical information: T1N0, T2N0, T3N0, N1, N2, and N3, where Ni means that a sample has metastasized to i lymph nodes for i $\le \,2$, and to at least 3 nodes for i = 3; and TjN0 means that the non-metastasized sample is at stage j. The detailed staging information of all the tumor samples is given in Supplementary Table S8.

### Data processing and basic analyses

We have conducted differential gene-expression analyses over all the tumor samples of each stage against controls defined above using Deseq2 in the R package^60^. A gene is considered differentially expressed in cancer vs. controls if its fold change is greater than 2.0 with p-value < 0.05^61^.

Co-expression analyses are carried out based on the transcripts per million (TPM) levels among selected genes, measured using the Spearman’s rank correlation coefficient and R^2^ ≥ 0.8 and p-value < 0.05 as the thresholds^62,63^. Gene length is calculated using GDC reference files https://gdc.cancer.gov/about-data/data-harmonization-and-generation/gdc-reference-files. Heatmap.2 of the gplots package of the R statistical computing and graphics software environment^64^, was used to generate a heatmap for co-expressions between specified neural and non-neural genes, with neural genes represented as rows and non-neural genes as columns, and the value in each cell being the correlation level between a neural and a non-neural gene defined above. Pathway enrichment analyses are conducted using topGO with p-value < 0.05 as the cutoff ^65^.

### Linear regression

A generalized linear regression is conducted to identify non-neural pathways whose expressions can statistically represent (or explain) the observed expressions of a given set of neural pathways. We have normalized all gene-expression values across all breast cancer samples, using the min-max normalization to [0,1] after removing the unexpressed genes.

For a set of given neural pathways and their genes $\{{g}_{1},\,{g}_{2},\,\cdots ,{g}_{k}\}$, let $Y\in {{\mathbb{R}}}^{m\times k}$ be its gene-expression matrix over m samples and k genes. Our goal is to identify a set of non-neural pathways and their genes $X\in {{\mathbb{R}}}^{m\times n}$ and find a matrix $B\,\in \,{{\mathbb{R}}}^{n\times k}\,$ so that *||E||* is as small as possible, where $E=Y-XB$ is an error matrix. This problem can be formulated as the following linear regression problem with lasso penalty to avoid selecting too many non-neural genes to represent the given neural genes, where β refers to an element of B, k is the number of genes in Y, and n is the number of genes in X.

$$\mathop{\min }\limits_{\lambda  > 0,\,{\beta }_{i,j}}(||\,Y-XB{||}^{2}+\lambda \mathop{\sum }\limits_{(i,j)=(1,1)}^{(i,j)=(n,k)}{|{\beta }_{i,j}|}^{2}).$$

This problem can be considered as a Ridge regression problem and solved using the ridge function in the R package in an iterative fashion^66^. Initially, all up-regulated non-neural pathway genes are included as candidate genes for explaining the Y genes. For each round of regression, the program calculates a coefficient matrix B to give rise to a (local) minimal objective value along with an estimate of the level of contribution by each non-neural gene, measured using a p-value. Before the next iteration, the procedure removes those non-neural genes with insignificant p-values and then repeat the repression process using the selected genes until the procedure converges.

The calculated parameters in the final regression model for each category of neural functions along with the p-values of the selected non-neural genes are given in Supplementary Tables S4–S7. Three parameters: R^2^, adjusted R^2^ and p-value of the adjusted R^2^ are used to assess the quality of a regression result, where p-value is calculated based on random permutations repeated 10,000 times^67^.

## Supplementary information

Supplementary materials.

## Data Availability

Gene expression counts and clinical information of TNBC and NTNBC samples are available from The Cancer Genome Atlas (TCGA) database https://portal.gdc.cancer.gov.
