# Supplementary material for: Neural Functions Play Different Roles in Triple Negative Breast Cancer (TNBC) and non-TNBC
Source: Sci Rep. 2020 Feb 20;10:3065. doi: 10.1038/s41598-020-60030-5 (PMC7033128; doi:10.1038/s41598-020-60030-5)
Supplement: Supplementary file 1 — Supplementary materials. [file 41598_2020_60030_MOESM1_ESM.pdf]

Neural Functions Play Different Roles in Triple Negative Breast Cancer (TNBC) and non-TNBC

Renbo Tan[1,2,+], Haoyang Li[2,3,+], Zhenyu Huang[2], Yi Zhou[3], Mingxin Tao[2], Xin Gao[4], Ying Xu[2,3,5,*]

[1] School of Life Sciences, Tsinghua University, Beijing, 100084, China, tanrenbo@126.com
[2] Cancer Systems Biology Center, the China-Japan Union Hospital of Jilin University, Changchun, 130033, China
[3] College of Computer Science and Technology, Jilin University, Changchun, 130012, China
[4] Computational Bioscience Research Center, King Abdullah University of Science and Technology, Thuwal, 23955, Saudi Arabia
[5] Computational Systems Biology Lab, Department of Biochemistry and Molecular Biology and Institute of Bioinformatics, University of Georgia, Athens, 30602, USA
* Corresponding: xyn@uga.edu
+ These authors contributed equally to this work

Supplementary Figure S1-4: Heat-maps for correlations between non-neural genes and neural genes at stages N0/T1 N0/T2 N2 and N3. Supplementary Tables S1 and S2, up-regulated neural genes and NCN genes across different stages of TNBC and NTNBC. Supplementary Table S3, contributions of two major non-neural pathways to neural function in TNBC and NTNBC. Supplementary Tables S4 and S5, detain information of regression of Supplementary Table S3. Supplementary Tables S6 and S7, p-value and the difference of R^2 of TNBC and NTNBC, respectively. Supplementary Table S8, sample size distributed by staging information in TNBC and NTNBC.

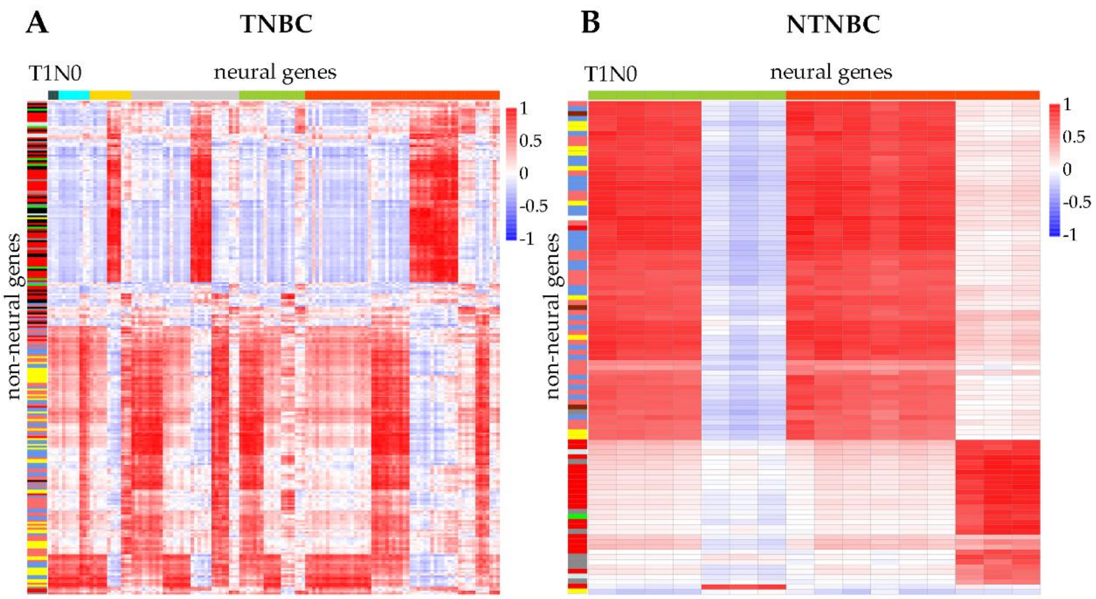

Supplementary Figure 1. Heat-maps for correlations between non-neural genes and neural genes at stages T1N0. The horizontal and vertical axes are selected neural and non-neural genes, respectively. The sequential order of the neural genes is determined as follows: dark blue for neurotransmitter secretion related genes, pink for neural crest formation related genes, light blue for axon and dendrite growth related genes, yellow for synapse formation related genes, gray for neuron projection related genes, dark green for neural structure formation in CNS related genes, orange for neuron differentiation related genes, and light green for glia development related genes. Non-neural genes are tagged by different color based on their function: red for cell adhesion, dusty blue for calcium sequestering, green for cytokine and chemokine production, gray for regulation of cell killing, white for ECM synthesis, black for hemopoiesis, purple for endocytosis, yellow for DNA damage and metabolism, pink for organelle assembly, and blue for cell skeleton synthesis, brown for de-development process.

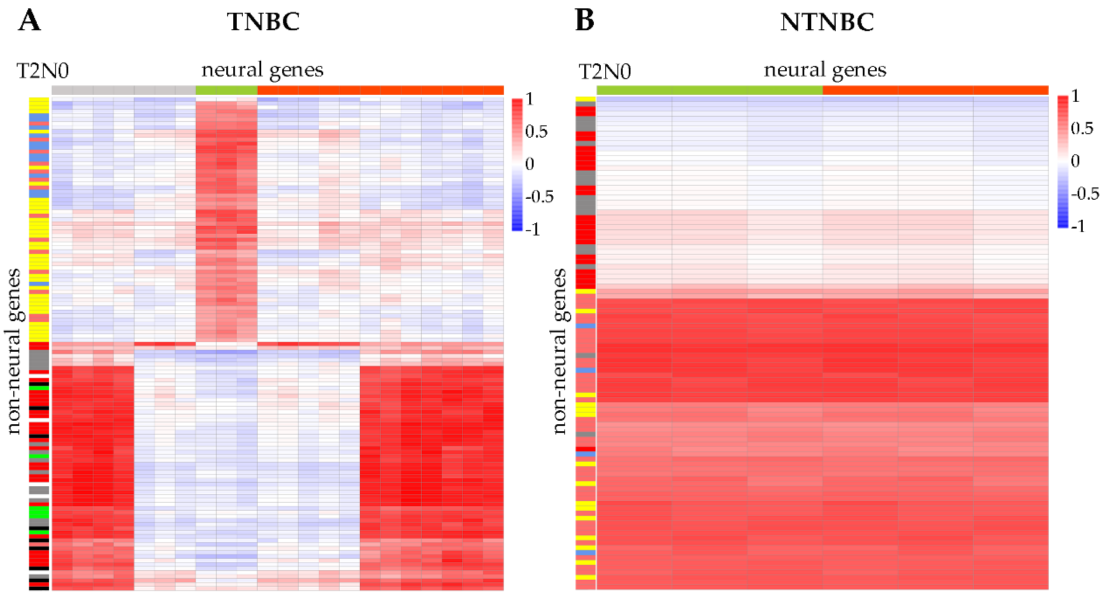

Supplementary Figure 2. Heat-maps for correlations between non-neural genes and neural genes at stages T2N0. The horizontal and vertical axes are selected neural and non-neural genes, respectively. The sequential order of the neural genes is determined as follows: dark blue for neurotransmitter secretion related genes, pink for neural crest formation related genes, light blue for axon and dendrite growth related genes, yellow for synapse formation related genes, gray for neuron projection related genes, dark green for neural structure formation in CNS related genes, orange for neuron differentiation related genes, and light green for glia development related genes. Non-neural genes are tagged by different color based on their function: red for cell adhesion, dusty blue for calcium sequestering, green for cytokine and chemokine production, gray for regulation of cell killing, white for ECM synthesis, black for hemopoiesis, purple for endocytosis, yellow for DNA damage and metabolism, pink for organelle assembly, and blue for cell skeleton synthesis, brown for de-development process.

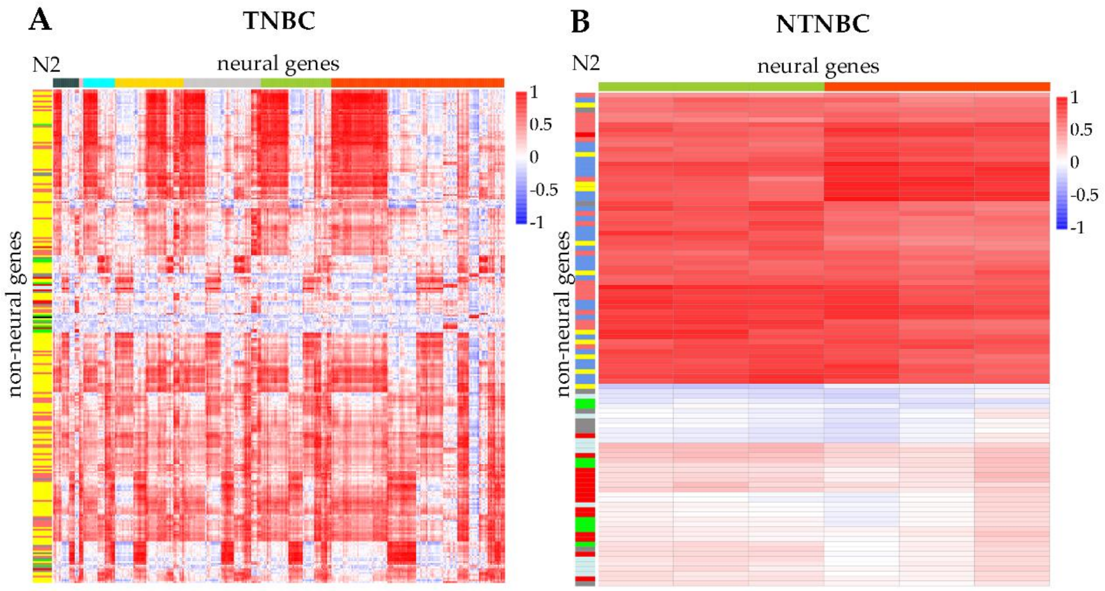

Supplementary Figure 3. Heat-maps for correlations between non-neural genes and neural genes at stages N2. The horizontal and vertical axes are selected neural and non-neural genes, respectively. The sequential order of the neural genes is determined as follows: dark blue for neurotransmitter secretion related genes, pink for neural crest formation related genes, light blue for axon and dendrite growth related genes, yellow for synapse formation related genes, gray for neuron projection related genes, dark green for neural structure formation in CNS related genes, orange for neuron differentiation related genes, and light green for glia development related genes. Non-neural genes are tagged by different color based on their function: red for cell adhesion, dusty blue for calcium sequestering, green for cytokine and chemokine production, gray for regulation of cell killing, white for ECM synthesis, black for hemopoiesis, purple for endocytosis, yellow for DNA damage and metabolism, pink for organelle assembly, and blue for cell skeleton synthesis, brown for de-development process.

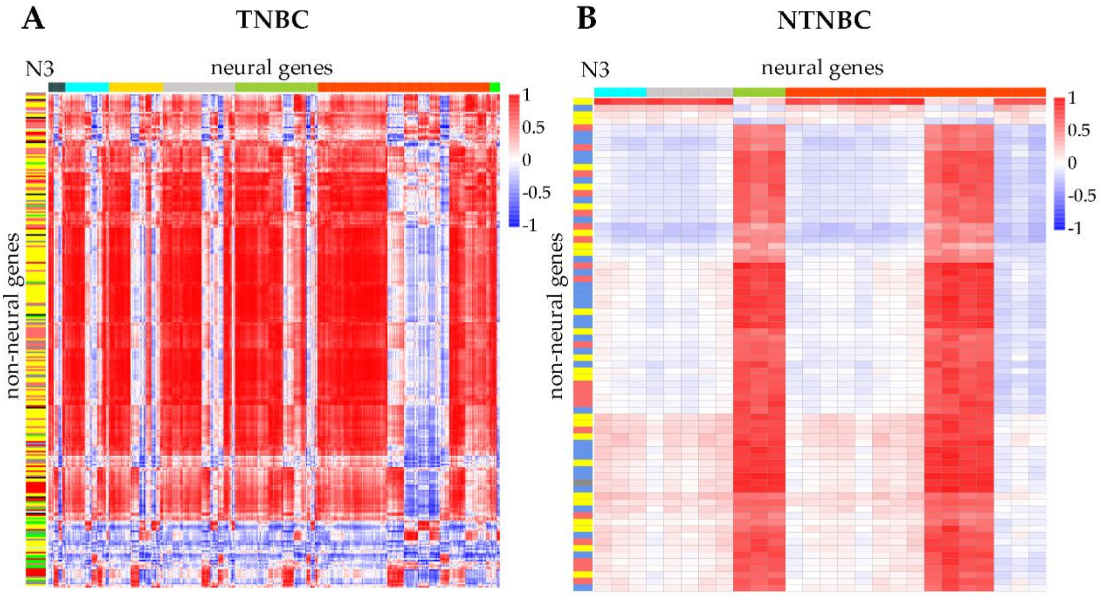

Supplementary Figure 4. Heat-maps for correlations between non-neural genes and neural genes at stages N3. The horizontal and vertical axes are selected neural and non-neural genes, respectively. The sequential order of the neural genes is determined as follows: dark blue for neurotransmitter secretion related genes, pink for neural crest formation related genes, light blue for axon and dendrite growth related genes, yellow for synapse formation related genes, gray for neuron projection related genes, dark green for neural structure formation in CNS related genes, orange for neuron differentiation related genes, and light green for glia development related genes. Non-neural genes are tagged by different color based on their function: red for cell adhesion, dusty blue for calcium sequestering, green for cytokine and chemokine production, gray for regulation of cell killing, white for ECM synthesis, black for hemopoiesis, purple for endocytosis, yellow for DNA damage and metabolism, pink for organelle assembly, and blue for cell skeleton synthesis, brown for de-development process.

Supplementary Table S1. Up-regulated neural genes across different stages of TNBC and NTNBC.

	**TNBC**	**NTNBC**
T1N0	545	640
T2N0	630	730
T3N0	530	652
N1	627	640
N2	459	606
N3	431	595

Supplementary Table S2. Up-regulated NCN genes across different stages of TNBC and NTNBC.

	**TNBC**	**NTNBC**
T1N0	415	83
T2N0	117	82
T3N0	523	114
N1	243	78
N2	443	85
N3	429	99

Supplementary Table S3. Contributions by cell-environment interaction and intracellular damage processing pathways to the induction of neural functions in TNBC and NTNBC, respectively.

	TNBC						NTNBC					
	**T1N0**	**T2N0**	**T3N0**	**N1**	**N2**	**N3**	**T1N0**	**T2N0**	**T3N0**	**N1**	**N2**	**N3**
neurotransmitter secretion	C-0<br>I-3		C-0<br>I-3	C-7<br>I-0	C-0<br>I-1							
neural crest development			C-0<br>I-2	C-6<br>I-0	C-0<br>I-2							
axon growth and dendrite pruning	C-0<br>I-5		C-0<br>I-1	C-2<br>I-0								
synapse formation	C-1<br>I-1	C-4<br>I-2			C-0<br>I-1							C-0<br>I-2
neuron projection and apoptosis		C-4<br>I-1	C-0<br>I-2	C-3<br>I-0			C-1<br>I-10	C-4<br>I-23	C-0<br>I-5	C-7<br>I-23	C-0<br>I-16	C-0<br>I-7
neural structure formation in CNS		C-2<br>I-2	C-0<br>I-2	C-1<br>I-0			C-1<br>I-5	C-1<br>I-20	C-0<br>I-9		C-0<br>I-5	
neuron differentiation	C-1<br>I-4	C-4<br>I-0	C-0<br>I-3	C-6<br>I-0			C-1<br>I-13	C-3<br>I-19	C-0<br>I-7	C-8<br>I-16	C-0<br>I-12	C-0<br>I-3
development of glia							C-1<br>I-9			C-5<br>I-13	C-0<br>I-7	C-0<br>I-3

n: C-i means the number i of pathways involved in cell-environment interactions;
I-j means the number j of pathways belonging to intracellular damage process.
A blank indicates that the expression of the neural function cannot be explained by these two categories of non-neural pathways directly, suggesting that the relevant neural functions require other non-neural functions to explain.

Supplementary Table S4. Information of cell-microenvironment interaction and intracellular damage processes contribution to TNBC.

neurotransmitter secretion (R^2_0.8894, adjR^2_0.8469, p-value_0.0019) at T1N0			
related non-neural pathways	gene number	gene name	p-value
cell cycle DNA replication	24	AICDA ATAD5 BRCA2 CDC45 CDC7 CDT1 CHEK2 DNA2 DONSON E2F7 E2F8 FBXO5 GINS1 LIG1 NUGGC PCNA POLA2 POLD1 PRIM2 RAD51 RFC2 RFC3 RFC4 SLBP	0.0227
regulation of metaphase/anaphase transition of cell cycle	21	ANAPC11 AURKB BUB1 BUB1B CCNB1 CDC6 CDT1 CENPE DLGAP5 ESPL1 FBXO5 GEN1 MAD2L1 MAD2L2 MAPK15 NDC80 PLK1 RAD21 SPDL1 TRIP13 TTK	0.0337
nuclear DNA replication	21	AICDA ATAD5 BRCA2 CDC45 CDC7 CDT1 CHEK2 DNA2 DONSON GINS1 LIG1 NUGGC PCNA POLA2 POLD1 PRIM2 RAD51 RFC2 RFC3 RFC4 SLBP	0.0337
axon growth and dendrite prunding (R^2_0.9376, adjR^2_0.8396, p-value_0.0019) at T1N0			
related non-neural pathways	gene number	gene name	p-value
metaphase/anaphase transition of cell cycle	21	ANAPC11 AURKB BUB1 BUB1B CCNB1 CDC6 CDT1 CENPE DLGAP5 ESPL1 FBXO5 GEN1 MAD2L1 MAD2L2 MAPK15 NDC80 PLK1 RAD21 SPDL1 TRIP13 TTK	0
regulation of mitotic sister chromatid separation	21	ANAPC11 AURKB BUB1 BUB1B CCNB1 CDC6 CDT1 CENPE DLGAP5 ESPL1 FBXO5 GEN1 MAD2L1 MAD2L2 NDC80 PLK1 PTTG1 RAD21 SPDL1 TRIP13 TTK	0.0003
regulation of chromosome separation	22	ANAPC11 AURKB BUB1 BUB1B CCNB1 CDC6 CDT1 CENPE DLGAP5 ESPL1 FBXO5 GEN1 MAD2L1 MAD2L2 MAPK15 NDC80 PLK1 PTTG1 RAD21 SPDL1 TRIP13 TTK	0.0068
regulation of metaphase/anaphase transition of cell cycle	21	ANAPC11 AURKB BUB1 BUB1B CCNB1 CDC6 CDT1 CENPE DLGAP5 ESPL1 FBXO5 GEN1 MAD2L1 MAD2L2 MAPK15 NDC80 PLK1 RAD21 SPDL1 TRIP13 TTK	0.012
regulation of mitotic metaphase/anaphase transition	20	ANAPC11 AURKB BUB1 BUB1B CCNB1 CDC6 CDT1 CENPE DLGAP5 ESPL1 FBXO5 GEN1 MAD2L1 MAD2L2 NDC80 PLK1 RAD21 SPDL1 TRIP13 TTK	0.012
synapse formation (R^2_0.9122, adjR^2_0.8683, p-value_0.0026) at T1N0			
related non-neural pathways	gene number	gene name	p-value
regulation of mitotic metaphase/anaphase transition	20	ANAPC11 AURKB BUB1 BUB1B CCNB1 CDC6 CDT1 CENPE DLGAP5 ESPL1 FBXO5 GEN1 MAD2L1 MAD2L2 NDC80 PLK1 RAD21 SPDL1 TRIP13 TTK	0.0135
positive regulation of leukocyte mediated cytotoxicity	20	CD1B CD1E CRTAM FAM49B HLA-A HLA-B HLA-C HLA-F HLA-G IL12RB1 IL21 IL23A LAG3 NCR3 PVR SH2D1A SLAMF6 ULBP1 ULBP3 VAV1	0.0332

neuron differentiation (R^2_0.9350, adjR^2_0.8330, p-value_0.0000) at T1N0			
related non-neural pathways	gene number	gene name	p-value
positive regulation of leukocyte mediated cytotoxicity	20	CD1B CD1E CRTAM FAM49B HLA-A HLA-B HLA-C HLA-F HLA-G IL12RB1 IL21 IL23A LAG3 NCR3 PVR SH2D1A SLAMF6 ULBP1 ULBP3 VAV1	0
nuclear DNA replication	21	AICDA ATAD5 BRCA2 CDC45 CDC7 CDT1 CHEK2 DNA2 DONSON GINS1 LIG1 NUGGC PCNA POLA2 POLD1 PRIM2 RAD51 RFC2 RFC3 RFC4 SLBP	0.0007
regulation of mitotic sister chromatid separation	21	ANAPC11 AURKB BUB1 BUB1B CCNB1 CDC6 CDT1 CENPE DLGAP5 ESPL1 FBXO5 GEN1 MAD2L1 MAD2L2 NDC80 PLK1 PTTG1 RAD21 SPDL1 TRIP13 TTK	0.0020
metaphase/anaphase transition of mitotic cell cycle	20	ANAPC11 AURKB BUB1 BUB1B CCNB1 CDC6 CDT1 CENPE DLGAP5 ESPL1 FBXO5 GEN1 MAD2L1 MAD2L2 NDC80 PLK1 RAD21 SPDL1 TRIP13 TTK	0.0019
regulation of chromosome separation	22	ANAPC11 AURKB BUB1 BUB1B CCNB1 CDC6 CDT1 CENPE DLGAP5 ESPL1 FBXO5 GEN1 MAD2L1 MAD2L2 MAPK15 NDC80 PLK1 PTTG1 RAD21 SPDL1 TRIP13 TTK	0.0476
synapse formation (R^2_0.7866, adjR^2_0.7025, p-value_0.0000) at T2N0			
related non-neural pathways	gene number	gene name	p-value
regulation of mitotic metaphase/anaphase transition	7	BUB1 BUB1B CCNB1 CDC6 ESPL1 NDC80 RAD21	0
alpha-beta T cell activation involved in immune response	8	CD80 CD86 FOXP3 IFNG IL12RB1 JAK3 SLAMF6 TNFSF4	0.0164
mitotic sister chromatid segregation	21	BUB1 BUB1B CCNB1 CDC6 CDCA8 ESPL1 KIF18A KIF18B KIF2C KIFC1 NCAPD2 NCAPG NCAPH NDC80 NEK2 NUSAP1 RAD21 SEH1L SMC2 SMC4 ZWINT	0.0078
alpha-beta T cell differentiation involved in immune response	8	CD80 CD86 FOXP3 IFNG IL12RB1 JAK3 SLAMF6 TNFSF4	0.0078
regulation of interleukin-12 production	7	CCR7 IFNG JAK3 LILRB1 LTB TIGIT TNFSF4	0.0201
CD4-positive, alpha-beta T cell activation	12	CD80 CD86 FOXP3 FUT7 IFNG IL12RB1 JAK3 PLA2G2D SASH3 SLAMF6 SOCS1 TNFSF4	0.0201
neuron projection and apoptosis (R^2_0.8903, adjR^2_0.8197, p-value_0.0060) at T2N0			

related non-neural pathways	gene number	gene name	p-value
lymphocyte costimulation	11	BTLA CCR7 CD5 CD80 CD86 CTLA4 ICOS LCK PDCD1 TNFSF13B TNFSF4	0.0248
interleukin-12 production	7	CCR7 IFNG JAK3 LILRB1 LTB TIGIT TNFSF4	0.0127
alpha-beta T cell differentiation	13	CD80 CD86 FOXP3 FUT7 GPR18 IFNG IL12RB1 JAK3 PLA2G2D SASH3 SLAMF6 SOCS1 TNFSF4	0.0127
regulation of mitotic metaphase/anaphase transition	7	BUB1 BUB1B CCNB1 CDC6 ESPL1 NDC80 RAD21	0.0344
alpha-beta T cell activation involved in immune response	8	CD80 CD86 FOXP3 IFNG IL12RB1 JAK3 SLAMF6 TNFSF4	0.0344
neural structure formation in CNS (R^2_0.8867, adjR^2_0.8421, p-value_0.0196) at T2N0			
related non-neural pathways	gene number	gene name	p-value
T cell costimulation	11	BTLA CCR7 CD5 CD80 CD86 CTLA4 ICOS LCK PDCD1 TNFSF13B TNFSF4	0.0118
mitotic sister chromatid segregation	21	BUB1 BUB1B CCNB1 CDC6 CDCA8 ESPL1 KIF18A KIF18B KIF2C KIFC1 NCAPD2 NCAPG NCAPH NDC80 NEK2 NUSAP1 RAD21 SEH1L SMC2 SMC4 ZWINT	0.0009
regulation of mitotic metaphase/anaphase transition	7	BUB1 BUB1B CCNB1 CDC6 ESPL1 NDC80 RAD21	0.0009
CD4-positive, alpha-beta T cell differentiation involved in immune response	8	CD80 CD86 FOXP3 IFNG IL12RB1 JAK3 SLAMF6 TNFSF4	0.0488
neuron differenetiation (R^2_0.8606, adjR^2_0.8167, p-value_0.0109) at T2N0			
related non-neural pathways	gene number	gene name	p-value
regulation of CD4-positive, alpha-beta T cell activation	9	CD80 CD86 FOXP3 IFNG IL12RB1 JAK3 SASH3 SOCS1 TNFSF4	0.0088
T cell differentiation involved in immune response	10	CD80 CD86 CLEC4E FCER1G FOXP3 IFNG IL12RB1 JAK3 SLAMF6 TNFSF4	0.0088

alpha-beta T cell differentiation involved in immune response	8	CD80 CD86 FOXP3 IFNG IL12RB1 JAK3 SLAMF6 TNFSF4	0.014
alpha-beta T cell activation involved in immune response	8	CD80 CD86 FOXP3 IFNG IL12RB1 JAK3 SLAMF6 TNFSF4	0.014
neurotransmitter secretion (R^2_0.9627, adjR^2_0.8320, p-value_0.0000) at T3N0			
related non-neural pathways	gene number	gene name	p-value
metaphase/anaphase transition of cell cycle	23	ANAPC11 AURKB BUB1 BUB1B CCNB1 CDC6 CDT1 CENPE DLGAP5 ESPL1 FBXO5 GEN1 MAD1L1 MAD2L1 MAD2L2 MAPK15 NDC80 NSMCE2 PLK1 RAD21 SPDL1 TRIP13 TTK	0.0397
regulation of mitotic metaphase/anaphase transition	22	ANAPC11 AURKB BUB1 BUB1B CCNB1 CDC6 CDT1 CENPE DLGAP5 ESPL1 FBXO5 GEN1 MAD1L1 MAD2L1 MAD2L2 NDC80 NSMCE2 PLK1 RAD21 SPDL1 TRIP13 TTK	0.0264
metaphase/anaphase transition of mitotic cell cycle	22	ANAPC11 AURKB BUB1 BUB1B CCNB1 CDC6 CDT1 CENPE DLGAP5 ESPL1 FBXO5 GEN1 MAD1L1 MAD2L1 MAD2L2 NDC80 NSMCE2 PLK1 RAD21 SPDL1 TRIP13 TTK	0.0252
neural crest development (R^2_0.9080, adjR^2_0.8816, p-value_0.0072) at T3N0			
related non-neural pathways	gene number	gene name	p-value
regulation of metaphase/anaphase transition of cell cycle	23	ANAPC11 AURKB BUB1 BUB1B CCNB1 CDC6 CDT1 CENPE DLGAP5 ESPL1 FBXO5 GEN1 MAD1L1 MAD2L1 MAD2L2 MAPK15 NDC80 NSMCE2 PLK1 RAD21 SPDL1 TRIP13 TTK	0.0023
regulation of mitotic metaphase/anaphase transition	22	ANAPC11 AURKB BUB1 BUB1B CCNB1 CDC6 CDT1 CENPE DLGAP5 ESPL1 FBXO5 GEN1 MAD1L1 MAD2L1 MAD2L2 NDC80 NSMCE2 PLK1 RAD21 SPDL1 TRIP13 TTK	0.0074
axon growth and dendrite prunding (R^2_0.9366, adjR^2_0.9050, p-value_0.0105) at T3N0			
related non-neural pathways	gene number	gene name	p-value
regulation of mitotic metaphase/anaphase transition	22	ANAPC11 AURKB BUB1 BUB1B CCNB1 CDC6 CDT1 CENPE DLGAP5 ESPL1 FBXO5 GEN1 MAD1L1 MAD2L1 MAD2L2 NDC80 NSMCE2 PLK1 RAD21 SPDL1 TRIP13 TTK	0.0012
neuron projection and apoptosis (R^2_0.9605, adjR^2_0.9408, p-value_0.011) at T3N0			
related non-neural pathways	gene number	gene name	p-value
metaphase/anaphase transition of cell cycle	23	ANAPC11 AURKB BUB1 BUB1B CCNB1 CDC6 CDT1 CENPE DLGAP5 ESPL1 FBXO5 GEN1 MAD1L1 MAD2L1 MAD2L2 MAPK15 NDC80 NSMCE2 PLK1 RAD21 SPDL1 TRIP13 TTK	0
regulation of mitotic metaphase/anaphase transition	22	ANAPC11 AURKB BUB1 BUB1B CCNB1 CDC6 CDT1 CENPE DLGAP5 ESPL1 FBXO5 GEN1 MAD1L1 MAD2L1 MAD2L2 NDC80 NSMCE2 PLK1 RAD21 SPDL1 TRIP13 TTK	0

neural structure formation in CNS (R^2_0.9751, adjR^2_0.9626, p-value_0) at T3N0			
related non-neural pathways	gene number	gene name	p-value
regulation of mitotic metaphase/anaphase transition	22	ANAPC11 AURKB BUB1 BUB1B CCNB1 CDC6 CDT1 CENPE DLGAP5 ESPL1 FBXO5 GEN1 MAD1L1 MAD2L1 MAD2L2 NDC80 NSMCE2 PLK1 RAD21 SPDL1 TRIP13 TTK	0
metaphase/anaphase transition of cell cycle	23	ANAPC11 AURKB BUB1 BUB1B CCNB1 CDC6 CDT1 CENPE DLGAP5 ESPL1 FBXO5 GEN1 MAD1L1 MAD2L1 MAD2L2 MAPK15 NDC80 NSMCE2 PLK1 RAD21 SPDL1 TRIP13 TTK	0
neuron differentiation (R^2_0.9199, adjR^2_0.8799, p-value_0.014) at T3N0			
related non-neural pathways	gene number	gene name	p-value
regulation of metaphase/anaphase transition of cell cycle	23	ANAPC11 AURKB BUB1 BUB1B CCNB1 CDC6 CDT1 CENPE DLGAP5 ESPL1 FBXO5 GEN1 MAD1L1 MAD2L1 MAD2L2 MAPK15 NDC80 NSMCE2 PLK1 RAD21 SPDL1 TRIP13 TTK	0.0215
metaphase/anaphase transition of cell cycle	23	ANAPC11 AURKB BUB1 BUB1B CCNB1 CDC6 CDT1 CENPE DLGAP5 ESPL1 FBXO5 GEN1 MAD1L1 MAD2L1 MAD2L2 MAPK15 NDC80 NSMCE2 PLK1 RAD21 SPDL1 TRIP13 TTK	0.0033
regulation of mitotic metaphase/anaphase transition	22	ANAPC11 AURKB BUB1 BUB1B CCNB1 CDC6 CDT1 CENPE DLGAP5 ESPL1 FBXO5 GEN1 MAD1L1 MAD2L1 MAD2L2 NDC80 NSMCE2 PLK1 RAD21 SPDL1 TRIP13 TTK	0.0018
neurotransmitter secretion (R^2_0.9704, adjR^2_0.8986, p-value_0) at N1			
related non-neural pathways	gene number	gene name	p-value
regulation of humoral immune response	51	C1QB C1QC C2 CCR7 CD19 CR2 CXCL13 IGHG1 IGHG2 IGHG4 IGHV1-2 IGHV1-69 IGHV2-5 IGHV3-11 IGHV3-23 IGHV3-30 IGHV3-33 IGHV3-53 IGHV4-34 IGHV4-39 IGHV4-59 IGKV1-33 IGKV1-39 IGKV1-5 IGKV1D-12 IGKV1D-16 IGKV1D-33 IGKV1D-39 IGKV2-28 IGKV2-30 IGKV2D-28 IGKV2D-30 IGKV2D-40 IGKV3-11 IGKV3-15 IGKV3-20 IGKV4-1 IGLC6 IGLV1-44 IGLV1-47 IGLV1-51 IGLV2-14 IGLV2-23 IGLV2-8 IGLV3-1 IGLV3-19 IGLV3-21 IGLV6-57 IGLV7-43 PTPN6 ZP3	0

Fc-gamma receptor signaling pathway involved in phagocytosis	52	CD247 CD3G FCGR1A FCGR2A FCGR3A HCK IGHG1 IGHG2 IGHG4 IGHV1-2 IGHV1-69 IGHV2-5 IGHV3-11 IGHV3-23 IGHV3-30 IGHV3-33 IGHV3-53 IGHV4-34 IGHV4-39 IGHV4-59 IGKV1-33 IGKV1-39 IGKV1-5 IGKV1D-12 IGKV1D-16 IGKV1D-33 IGKV1D-39 IGKV2-28 IGKV2-30 IGKV2D-28 IGKV2D-30 IGKV2D-40 IGKV3-11 IGKV3-15 IGKV3-20 IGKV4-1 IGLC6 IGLV1-44 IGLV1-47 IGLV1-51 IGLV2-14 IGLV2-23 IGLV2-8 IGLV3-1 IGLV3-19 IGLV3-21 IGLV6-57 IGLV7-43 MYO1G NCKAP1L VAV1 WAS	0.005
Fc receptor mediated stimulatory signaling pathway	53	CD247 CD3G FCER1G FCGR1A FCGR2A FCGR3A HCK IGHG1 IGHG2 IGHG4 IGHV1-2 IGHV1-69 IGHV2-5 IGHV3-11 IGHV3-23 IGHV3-30 IGHV3-33 IGHV3-53 IGHV4-34 IGHV4-39 IGHV4-59 IGKV1-33 IGKV1-39 IGKV1-5 IGKV1D-12 IGKV1D-16 IGKV1D-33 IGKV1D-39 IGKV2-28 IGKV2-30 IGKV2D-28 IGKV2D-30 IGKV2D-40 IGKV3-11 IGKV3-15 IGKV3-20 IGKV4-1 IGLC6 IGLV1-44 IGLV1-47 IGLV1-51 IGLV2-14 IGLV2-23 IGLV2-8 IGLV3-1 IGLV3-19 IGLV3-21 IGLV6-57 IGLV7-43 MYO1G NCKAP1L VAV1 WAS	0.0025
regulation of complement activation	47	C1QB C1QC C2 CD19 CR2 IGHG1 IGHG2 IGHG4 IGHV1-2 IGHV1-69 IGHV2-5 IGHV3-11 IGHV3-23 IGHV3-30 IGHV3-33 IGHV3-53 IGHV4-34 IGHV4-39 IGHV4-59 IGKV1-33 IGKV1-39 IGKV1-5 IGKV1D-12 IGKV1D-16 IGKV1D-33 IGKV1D-39 IGKV2-28 IGKV2-30 IGKV2D-28 IGKV2D-30 IGKV2D-40 IGKV3-11 IGKV3-15 IGKV3-20 IGKV4-1 IGLC6 IGLV1-44 IGLV1-47 IGLV1-51 IGLV2-14 IGLV2-23 IGLV2-8 IGLV3-1 IGLV3-19 IGLV3-21 IGLV6-57 IGLV7-43	0.0064
Fc-epsilon receptor signaling pathway	48	BTK CARD11 FCER1G IGHV1-2 IGHV1-69 IGHV2-5 IGHV3-11 IGHV3-23 IGHV3-30 IGHV3-33 IGHV3-53 IGHV4-34 IGHV4-39 IGHV4-59 IGKV1-33 IGKV1-39 IGKV1-5 IGKV1D-12 IGKV1D-16 IGKV1D-33 IGKV1D-39 IGKV2-28 IGKV2-30 IGKV2D-28 IGKV2D-30 IGKV2D-40 IGKV3-11 IGKV3-15 IGKV3-20 IGKV4-1 IGLC6 IGLV1-44 IGLV1-47 IGLV1-51 IGLV2-14 IGLV2-23 IGLV2-8 IGLV3-1 IGLV3-19 IGLV3-21 IGLV6-57 IGLV7-43 ITK LAT LAT2 LCP2 LILRA4 VAV1	0.0021

phagocytosis, recognition	41	IGHG1 IGHG2 IGHG4 IGHM IGHV1-18 IGHV1-2 IGHV1-69 IGHV1OR15-9 IGHV1OR21-1 IGHV2-5 IGHV3-11 IGHV3-15 IGHV3-20 IGHV3-21 IGHV3-23 IGHV3-30 IGHV3-33 IGHV3-43 IGHV3-49 IGHV3-53 IGHV3-64 IGHV3-66 IGHV3-74 IGHV3OR16-10 IGHV3OR16-13 IGHV3OR16-8 IGHV3OR16-9 IGHV4-28 IGHV4-31 IGHV4-34 IGHV4-39 IGHV4-59 IGHV4-61 IGHV4OR15-8 IGHV5-51 IGLC2 IGLC3 IGLC6 IGLL1 IGLL5 TRBC2	0.0165
B cell mediated immunity	88	ATAD5 BTK C17orf99 C1QB C1QC C2 CD27 CD28 CR2 FCER1G FOXP3 IGHG1 IGHG2 IGHG4 IGHM IGHV1-18 IGHV1-2 IGHV1-69 IGHV1OR15-9 IGHV1OR21-1 IGHV2-5 IGHV3-11 IGHV3-15 IGHV3-20 IGHV3-21 IGHV3-23 IGHV3-30 IGHV3-33 IGHV3-43 IGHV3-49 IGHV3-53 IGHV3-64 IGHV3-66 IGHV3-74 IGHV3OR16-10 IGHV3OR16-13 IGHV3OR16-8 IGHV3OR16-9 IGHV4-28 IGHV4-31 IGHV4-34 IGHV4-39 IGHV4-59 IGHV4-61 IGHV4OR15-8 IGHV5-51 IGKV1-33 IGKV1-39 IGKV1-5 IGKV1D-12 IGKV1D-16 IGKV1D-33 IGKV1D-39 IGKV2-28 IGKV2-30 IGKV2D-28 IGKV2D-30 IGKV2D-40 IGKV3-11 IGKV3-15 IGKV3-20 IGKV4-1 IGLC2 IGLC3 IGLC6 IGLL1 IGLL5 IGLV1-44 IGLV1-47 IGLV1-51 IGLV2-14 IGLV2-23 IGLV2-8 IGLV3-1 IGLV3-19 IGLV3-21 IGLV6-57 IGLV7-43 IRF7 MSH6 POU2F2 PTPN6 RNF8 SLA2 TBX21 TRBC2 UNG ZP3	0.0289
neural crest development (R^2_0.6945, adjR^2_0.3335, p-value_0.0011) at N1			
related non-neural pathways	gene number	gene name	p-value
regulation of humoral immune response	51	C1QB C1QC C2 CCR7 CD19 CR2 CXCL13 IGHG1 IGHG2 IGHG4 IGHV1-2 IGHV1-69 IGHV2-5 IGHV3-11 IGHV3-23 IGHV3-30 IGHV3-33 IGHV3-53 IGHV4-34 IGHV4-39 IGHV4-59 IGKV1-33 IGKV1-39 IGKV1-5 IGKV1D-12 IGKV1D-16 IGKV1D-33 IGKV1D-39 IGKV2-28 IGKV2-30 IGKV2D-28 IGKV2D-30 IGKV2D-40 IGKV3-11 IGKV3-15 IGKV3-20 IGKV4-1 IGLC6 IGLV1-44 IGLV1-47 IGLV1-51 IGLV2-14 IGLV2-23 IGLV2-8 IGLV3-1 IGLV3-19 IGLV3-21 IGLV6-57 IGLV7-43 PTPN6 ZP3	0

phagocytosis, recognition	41	IGHG1 IGHG2 IGHG4 IGHM IGHV1-18 IGHV1-2 IGHV1-69 IGHV1OR15-9 IGHV1OR21-1 IGHV2-5 IGHV3-11 IGHV3-15 IGHV3-20 IGHV3-21 IGHV3-23 IGHV3-30 IGHV3-33 IGHV3-43 IGHV3-49 IGHV3-53 IGHV3-64 IGHV3-66 IGHV3-74 IGHV3OR16-10 IGHV3OR16-13 IGHV3OR16-8 IGHV3OR16-9 IGHV4-28 IGHV4-31 IGHV4-34 IGHV4-39 IGHV4-59 IGHV4-61 IGHV4OR15-8 IGHV5-51 IGLC2 IGLC3 IGLC6 IGLL1 IGLL5 TRBC2	0
regulation of complement activation	47	C1QB C1QC C2 CD19 CR2 IGHG1 IGHG2 IGHG4 IGHV1-2 IGHV1-69 IGHV2-5 IGHV3-11 IGHV3-23 IGHV3-30 IGHV3-33 IGHV3-53 IGHV4-34 IGHV4-39 IGHV4-59 IGKV1-33 IGKV1-39 IGKV1-5 IGKV1D-12 IGKV1D-16 IGKV1D-33 IGKV1D-39 IGKV2-28 IGKV2-30 IGKV2D-28 IGKV2D-30 IGKV2D-40 IGKV3-11 IGKV3-15 IGKV3-20 IGKV4-1 IGLC6 IGLV1-44 IGLV1-47 IGLV1-51 IGLV2-14 IGLV2-23 IGLV2-8 IGLV3-1 IGLV3-19 IGLV3-21 IGLV6-57 IGLV7-43	0.0019
regulation of protein activation cascade	47	C1QB C1QC C2 CD19 CR2 IGHG1 IGHG2 IGHG4 IGHV1-2 IGHV1-69 IGHV2-5 IGHV3-11 IGHV3-23 IGHV3-30 IGHV3-33 IGHV3-53 IGHV4-34 IGHV4-39 IGHV4-59 IGKV1-33 IGKV1-39 IGKV1-5 IGKV1D-12 IGKV1D-16 IGKV1D-33 IGKV1D-39 IGKV2-28 IGKV2-30 IGKV2D-28 IGKV2D-30 IGKV2D-40 IGKV3-11 IGKV3-15 IGKV3-20 IGKV4-1 IGLC6 IGLV1-44 IGLV1-47 IGLV1-51 IGLV2-14 IGLV2-23 IGLV2-8 IGLV3-1 IGLV3-19 IGLV3-21 IGLV6-57 IGLV7-43	0.0007
Fc-epsilon receptor signaling pathway	48	BTK CARD11 FCER1G IGHV1-2 IGHV1-69 IGHV2-5 IGHV3-11 IGHV3-23 IGHV3-30 IGHV3-33 IGHV3-53 IGHV4-34 IGHV4-39 IGHV4-59 IGKV1-33 IGKV1-39 IGKV1-5 IGKV1D-12 IGKV1D-16 IGKV1D-33 IGKV1D-39 IGKV2-28 IGKV2-30 IGKV2D-28 IGKV2D-30 IGKV2D-40 IGKV3-11 IGKV3-15 IGKV3-20 IGKV4-1 IGLC6 IGLV1-44 IGLV1-47 IGLV1-51 IGLV2-14 IGLV2-23 IGLV2-8 IGLV3-1 IGLV3-19 IGLV3-21 IGLV6-57 IGLV7-43 ITK LAT LAT2 LCP2 LILRA4 VAV1	0.0007

Fc-gamma receptor signaling pathway	54	CD247 CD3G CLEC4E FCER1G FCGR1A FCGR2A FCGR3A HCK IGHG1 IGHG2 IGHG4 IGHV1-2 IGHV1-69 IGHV2-5 IGHV3-11 IGHV3-23 IGHV3-30 IGHV3-33 IGHV3-53 IGHV4-34 IGHV4-39 IGHV4-59 IGKV1-33 IGKV1-39 IGKV1-5 IGKV1D-12 IGKV1D-16 IGKV1D-33 IGKV1D-39 IGKV2-28 IGKV2-30 IGKV2D-28 IGKV2D-30 IGKV2D-40 IGKV3-11 IGKV3-15 IGKV3-20 IGKV4-1 IGLC6 IGLV1-44 IGLV1-47 IGLV1-51 IGLV2-14 IGLV2-23 IGLV2-8 IGLV3-1 IGLV3-19 IGLV3-21 IGLV6-57 IGLV7-43 MYO1G NCKAP1L VAV1 WAS	0.0008
axon growth and dendrite pruning (R^2_0.7675, adjR^2_0.6513, p-value_0.004) at N1			
related non-neural pathways	gene number	gene name	p-value
Fc-epsilon receptor signaling pathway	48	BTK CARD11 FCER1G IGHV1-2 IGHV1-69 IGHV2-5 IGHV3-11 IGHV3-23 IGHV3-30 IGHV3-33 IGHV3-53 IGHV4-34 IGHV4-39 IGHV4-59 IGKV1-33 IGKV1-39 IGKV1-5 IGKV1D-12 IGKV1D-16 IGKV1D-33 IGKV1D-39 IGKV2-28 IGKV2-30 IGKV2D-28 IGKV2D-30 IGKV2D-40 IGKV3-11 IGKV3-15 IGKV3-20 IGKV4-1 IGLC6 IGLV1-44 IGLV1-47 IGLV1-51 IGLV2-14 IGLV2-23 IGLV2-8 IGLV3-1 IGLV3-19 IGLV3-21 IGLV6-57 IGLV7-43 ITK LAT LAT2 LCP2 LILRA4 VAV1	0.0037
regulation of complement activation	47	C1QB C1QC C2 CD19 CR2 IGHG1 IGHG2 IGHG4 IGHV1-2 IGHV1-69 IGHV2-5 IGHV3-11 IGHV3-23 IGHV3-30 IGHV3-33 IGHV3-53 IGHV4-34 IGHV4-39 IGHV4-59 IGKV1-33 IGKV1-39 IGKV1-5 IGKV1D-12 IGKV1D-16 IGKV1D-33 IGKV1D-39 IGKV2-28 IGKV2-30 IGKV2D-28 IGKV2D-30 IGKV2D-40 IGKV3-11 IGKV3-15 IGKV3-20 IGKV4-1 IGLC6 IGLV1-44 IGLV1-47 IGLV1-51 IGLV2-14 IGLV2-23 IGLV2-8 IGLV3-1 IGLV3-19 IGLV3-21 IGLV6-57 IGLV7-43	0.0037
neuron projection and apoptosis (R^2_0.8921, adjR^2_0.741, p-value_0.0115) at N1			
related non-neural pathways	gene number	gene name	p-value

B cell mediated immunity	88	ATAD5 BTK C17orf99 C1QB C1QC C2 CD27 CD28 CR2 FCER1G FOXP3 IGHG1 IGHG2 IGHG4 IGHM IGHV1-18 IGHV1-2 IGHV1-69 IGHV1OR15-9 IGHV1OR21-1 IGHV2-5 IGHV3-11 IGHV3-15 IGHV3-20 IGHV3-21 IGHV3-23 IGHV3-30 IGHV3-33 IGHV3-43 IGHV3-49 IGHV3-53 IGHV3-64 IGHV3-66 IGHV3-74 IGHV3OR16-10 IGHV3OR16-13 IGHV3OR16-8 IGHV3OR16-9 IGHV4-28 IGHV4-31 IGHV4-34 IGHV4-39 IGHV4-59 IGHV4-61 IGHV4OR15-8 IGHV5-51 IGKV1-33 IGKV1-39 IGKV1-5 IGKV1D-12 IGKV1D-16 IGKV1D-33 IGKV1D-39 IGKV2-28 IGKV2-30 IGKV2D-28 IGKV2D-30 IGKV2D-40 IGKV3-11 IGKV3-15 IGKV3-20 IGKV4-1 IGLC2 IGLC3 IGLC6 IGLL1 IGLL5 IGLV1-44 IGLV1-47 IGLV1-51 IGLV2-14 IGLV2-23 IGLV2-8 IGLV3-1 IGLV3-19 IGLV3-21 IGLV6-57 IGLV7-43 IRF7 MSH6 POU2F2 PTPN6 RNF8 SLA2 TBX21 TRBC2 UNG ZP3	0.0447
complement activation	73	C1QB C1QC C2 CD19 CR2 IGHG1 IGHG2 IGHG4 IGHM IGHV1-18 IGHV1-2 IGHV1-69 IGHV1OR15-9 IGHV1OR21-1 IGHV2-5 IGHV3-11 IGHV3-15 IGHV3-20 IGHV3-21 IGHV3-23 IGHV3-30 IGHV3-33 IGHV3-43 IGHV3-49 IGHV3-53 IGHV3-64 IGHV3-66 IGHV3-74 IGHV3OR16-10 IGHV3OR16-13 IGHV3OR16-8 IGHV3OR16-9 IGHV4-28 IGHV4-31 IGHV4-34 IGHV4-39 IGHV4-59 IGHV4-61 IGHV4OR15-8 IGHV5-51 IGKV1-33 IGKV1-39 IGKV1-5 IGKV1D-12 IGKV1D-16 IGKV1D-33 IGKV1D-39 IGKV2-28 IGKV2-30 IGKV2D-28 IGKV2D-30 IGKV2D-40 IGKV3-11 IGKV3-15 IGKV3-20 IGKV4-1 IGLC2 IGLC3 IGLC6 IGLL1 IGLL5 IGLV1-44 IGLV1-47 IGLV1-51 IGLV2-14 IGLV2-23 IGLV2-8 IGLV3-1 IGLV3-19 IGLV3-21 IGLV6-57 IGLV7-43 TRBC2	0.0447
regulation of humoral immune response	51	C1QB C1QC C2 CCR7 CD19 CR2 CXCL13 IGHG1 IGHG2 IGHG4 IGHV1-2 IGHV1-69 IGHV2-5 IGHV3-11 IGHV3-23 IGHV3-30 IGHV3-33 IGHV3-53 IGHV4-34 IGHV4-39 IGHV4-59 IGKV1-33 IGKV1-39 IGKV1-5 IGKV1D-12 IGKV1D-16 IGKV1D-33 IGKV1D-39 IGKV2-28 IGKV2-30 IGKV2D-28 IGKV2D-30 IGKV2D-40 IGKV3-11 IGKV3-15 IGKV3-20 IGKV4-1 IGLC6 IGLV1-44 IGLV1-47 IGLV1-51 IGLV2-14 IGLV2-23 IGLV2-8 IGLV3-1 IGLV3-19 IGLV3-21 IGLV6-57 IGLV7-43 PTPN6 ZP3	0.0261
neural structure formation in CNS (R^2_0.8951, adjR^2_0.7901, p-value_0.0033) at N1			
related non-neural pathways	gene number	gene name	p-value

Fc-gamma receptor signaling pathway involved in phagocytosis	52	CD247 CD3G FCGR1A FCGR2A FCGR3A HCK IGHG1 IGHG2 IGHG4 IGHV1-2 IGHV1-69 IGHV2-5 IGHV3-11 IGHV3-23 IGHV3-30 IGHV3-33 IGHV3-53 IGHV4-34 IGHV4-39 IGHV4-59 IGKV1-33 IGKV1-39 IGKV1-5 IGKV1D-12 IGKV1D-16 IGKV1D-33 IGKV1D-39 IGKV2-28 IGKV2-30 IGKV2D-28 IGKV2D-30 IGKV2D-40 IGKV3-11 IGKV3-15 IGKV3-20 IGKV4-1 IGLC6 IGLV1-44 IGLV1-47 IGLV1-51 IGLV2-14 IGLV2-23 IGLV2-8 IGLV3-1 IGLV3-19 IGLV3-21 IGLV6-57 IGLV7-43 MYO1G NCKAP1L VAV1 WAS	0.0195
neuron differentiation (R^2_0.8979, adjR^2_0.7772, p-value_0) at N1			
related non-neural pathways	gene number	gene name	p-value
phagocytosis, recognition	41	IGHG1 IGHG2 IGHG4 IGHM IGHV1-18 IGHV1-2 IGHV1-69 IGHV1OR15-9 IGHV1OR21-1 IGHV2-5 IGHV3-11 IGHV3-15 IGHV3-20 IGHV3-21 IGHV3-23 IGHV3-30 IGHV3-33 IGHV3-43 IGHV3-49 IGHV3-53 IGHV3-64 IGHV3-66 IGHV3-74 IGHV3OR16-10 IGHV3OR16-13 IGHV3OR16-8 IGHV3OR16-9 IGHV4-28 IGHV4-31 IGHV4-34 IGHV4-39 IGHV4-59 IGHV4-61 IGHV4OR15-8 IGHV5-51 IGLC2 IGLC3 IGLC6 IGLL1 IGLL5 TRBC2	0.0003
Fc-gamma receptor signaling pathway	54	CD247 CD3G CLEC4E FCER1G FCGR1A FCGR2A FCGR3A HCK IGHG1 IGHG2 IGHG4 IGHV1-2 IGHV1-69 IGHV2-5 IGHV3-11 IGHV3-23 IGHV3-30 IGHV3-33 IGHV3-53 IGHV4-34 IGHV4-39 IGHV4-59 IGKV1-33 IGKV1-39 IGKV1-5 IGKV1D-12 IGKV1D-16 IGKV1D-33 IGKV1D-39 IGKV2-28 IGKV2-30 IGKV2D-28 IGKV2D-30 IGKV2D-40 IGKV3-11 IGKV3-15 IGKV3-20 IGKV4-1 IGLC6 IGLV1-44 IGLV1-47 IGLV1-51 IGLV2-14 IGLV2-23 IGLV2-8 IGLV3-1 IGLV3-19 IGLV3-21 IGLV6-57 IGLV7-43 MYO1G NCKAP1L VAV1 WAS	0.0001
regulation of humoral immune response	51	C1QB C1QC C2 CCR7 CD19 CR2 CXCL13 IGHG1 IGHG2 IGHG4 IGHV1-2 IGHV1-69 IGHV2-5 IGHV3-11 IGHV3-23 IGHV3-30 IGHV3-33 IGHV3-53 IGHV4-34 IGHV4-39 IGHV4-59 IGKV1-33 IGKV1-39 IGKV1-5 IGKV1D-12 IGKV1D-16 IGKV1D-33 IGKV1D-39 IGKV2-28 IGKV2-30 IGKV2D-28 IGKV2D-30 IGKV2D-40 IGKV3-11 IGKV3-15 IGKV3-20 IGKV4-1 IGLC6 IGLV1-44 IGLV1-47 IGLV1-51 IGLV2-14 IGLV2-23 IGLV2-8 IGLV3-1 IGLV3-19 IGLV3-21 IGLV6-57 IGLV7-43 PTPN6 ZP3	0.0002

B cell receptor signaling pathway	60	BLK BTK CD19 CD22 CD300A CD79A CD79B CTLA4 FCRL3 IGHG1 IGHG2 IGHG4 IGHM IGHV1-18 IGHV1-2 IGHV1-69 IGHV1OR15-9 IGHV1OR21-1 IGHV2-5 IGHV3-11 IGHV3-15 IGHV3-20 IGHV3-21 IGHV3-23 IGHV3-30 IGHV3-33 IGHV3-43 IGHV3-49 IGHV3-53 IGHV3-64 IGHV3-66 IGHV3-74 IGHV3OR16-10 IGHV3OR16-13 IGHV3OR16-8 IGHV3OR16-9 IGHV4-28 IGHV4-31 IGHV4-34 IGHV4-39 IGHV4-59 IGHV4-61 IGHV4OR15-8 IGHV5-51 IGLC2 IGLC3 IGLC6 IGLL1 IGLL5 ITK KLHL6 LAT2 LCK LPXN MNDA NCKAP1L NFAM1 PTPN22 PTPN6 TRBC2	0.0081
humoral immune response mediated by circulating immunoglobulin	74	C1QB C1QC C2 CR2 IGHG1 IGHG2 IGHG4 IGHM IGHV1-18 IGHV1-2 IGHV1-69 IGHV1OR15-9 IGHV1OR21-1 IGHV2-5 IGHV3-11 IGHV3-15 IGHV3-20 IGHV3-21 IGHV3-23 IGHV3-30 IGHV3-33 IGHV3-43 IGHV3-49 IGHV3-53 IGHV3-64 IGHV3-66 IGHV3-74 IGHV3OR16-10 IGHV3OR16-13 IGHV3OR16-8 IGHV3OR16-9 IGHV4-28 IGHV4-31 IGHV4-34 IGHV4-39 IGHV4-59 IGHV4-61 IGHV4OR15-8 IGHV5-51 IGKV1-33 IGKV1-39 IGKV1-5 IGKV1D-12 IGKV1D-16 IGKV1D-33 IGKV1D-39 IGKV2-28 IGKV2-30 IGKV2D-28 IGKV2D-30 IGKV2D-40 IGKV3-11 IGKV3-15 IGKV3-20 IGKV4-1 IGLC2 IGLC3 IGLC6 IGLL1 IGLL5 IGLV1-44 IGLV1-47 IGLV1-51 IGLV2-14 IGLV2-23 IGLV2-8 IGLV3-1 IGLV3-19 IGLV3-21 IGLV6-57 IGLV7-43 PTPN6 TRBC2 ZP3	0.0049
Fc-epsilon receptor signaling pathway	48	BTK CARD11 FCER1G IGHV1-2 IGHV1-69 IGHV2-5 IGHV3-11 IGHV3-23 IGHV3-30 IGHV3-33 IGHV3-53 IGHV4-34 IGHV4-39 IGHV4-59 IGKV1-33 IGKV1-39 IGKV1-5 IGKV1D-12 IGKV1D-16 IGKV1D-33 IGKV1D-39 IGKV2-28 IGKV2-30 IGKV2D-28 IGKV2D-30 IGKV2D-40 IGKV3-11 IGKV3-15 IGKV3-20 IGKV4-1 IGLC6 IGLV1-44 IGLV1-47 IGLV1-51 IGLV2-14 IGLV2-23 IGLV2-8 IGLV3-1 IGLV3-19 IGLV3-21 IGLV6-57 IGLV7-43 ITK LAT LAT2 LCP2 LILRA4 VAV1	0.0049
neurotransmitter secretion (R^2_0.8202, adjR^2_0.7528, p-value_0.0046) at N2			
related non-neural pathways	gene number	gene name	p-value
regulation of metaphase/anaphase transition of cell cycle	20	AURKB BUB1 BUB1B CCNB1 CDC6 CDT1 CENPE DLGAP5 ESPL1 FBXO5 GEN1 MAD2L1 MAD2L2 MAPK15 NDC80 PLK1 RAD21 SPDL1 TRIP13 TTK	0.0162
neural crest development (R^2_0.9624, adjR^2_0.8967, p-value_0) at N2			

related non-neural pathways	gene number	gene name	p-value
DNA-dependent DNA replication	62	ALYREF ATAD5 BAZ1A BLM BRCA2 CCNE1 CCNE2 CDC45 CDC6 CDC7 CDK2 CDK2AP1 CDT1 CHEK2 CHTF18 DDX11 DNA2 DONSON DSCC1 E2F7 E2F8 EME1 FBXO5 GEN1 GINS1 GINS2 GINS3 GINS4 GMNN HMGA1 LIG1 LRWD1 MCM10 MCM2 MCM3 MCM4 MCM5 MCM6 MCM7 MMS22L MSH6 ORC1 ORC6 PCNA POLA2 POLD1 POLE POLE2 POLQ PRIM2 RAD51 RFC2 RFC4 RFC5 RFWD3 RNASEH2A STOML2 TICRR TIMELESS TONSL WDHD1 WRNIP1	0.0467
regulation of metaphase/anaphase transition of cell cycle	20	AURKB BUB1 BUB1B CCNB1 CDC6 CDT1 CENPE DLGAP5 ESPL1 FBXO5 GEN1 MAD2L1 MAD2L2 MAPK15 NDC80 PLK1 RAD21 SPDL1 TRIP13 TTK	0.0285
synapse formation (R^2_0.8946, adjR^2_0.8550, p-value_0.0022) at N2			
related non-neural pathways	gene number	gene name	p-value
regulation of DNA-dependent DNA replication	21	BLM BRCA2 CDC7 CDK2 CDT1 CHEK2 CHTF18 DONSON DSCC1 E2F7 E2F8 FBXO5 GMNN MSH6 RFC2 RFC4 RFC5 STOML2 TICRR TIMELESS WRNIP1	0.0034

Supplementary Table S5. Information of cell-microenvironment interaction and intracellular damage processes contribution to NTNBC.

neuron projection and apoptosis (R^2_0.8596, adjR^2_0.8411, p-value_0) at T1N0			
related non-neural pathways	gene number	gene name	p-value
negative regulation of mitotic nuclear division	11	AURKB BUB1 BUB1B CCNB1 CDT1 MAD2L1 NDC80 PLK1 PTTG1 TRIP13 TTK	0.0001
metaphase/anaphase transition of cell cycle	13	AURKB BUB1 BUB1B CCNB1 CDT1 CENPE DLGAP5 ESPL1 MAD2L1 NDC80 PLK1 TRIP13 TTK	0
metaphase/anaphase transition of mitotic cell cycle	13	AURKB BUB1 BUB1B CCNB1 CDT1 CENPE DLGAP5 ESPL1 MAD2L1 NDC80 PLK1 TRIP13 TTK	0
chromosome localization	14	CCNB1 CDCA5 CDCA8 CDT1 CENPE CEP55 DLGAP5 FAM83D KIF18A KIF2C KIFC1 NDC80 NUF2 SPAG5	0.0131
mitotic sister chromatid separation	14	AURKB BUB1 BUB1B CCNB1 CDT1 CENPE DLGAP5 ESPL1 MAD2L1 NDC80 PLK1 PTTG1 TRIP13 TTK	0.0014
mitotic sister chromatid segregation	31	AURKB BUB1 BUB1B CCNB1 CDCA5 CDCA8 CDT1 CENPE CEP55 DLGAP5 ESPL1 KIF18A KIF18B KIF23 KIF2C KIFC1 MAD2L1 NCAPG NCAPH NDC80 NEK2 NUF2 NUSAP1 PLK1 PRC1 PTTG1 SMC4 SPAG5 TRIP13 TTK ZWINT	0.0081
regulation of sister chromatid segregation	15	AURKB BUB1 BUB1B CCNB1 CDCA5 CDT1 CENPE DLGAP5 ESPL1 MAD2L1 NDC80 PLK1 PTTG1 TRIP13 TTK	0.025
regulation of chromosome separation	14	AURKB BUB1 BUB1B CCNB1 CDT1 CENPE DLGAP5 ESPL1 MAD2L1 NDC80 PLK1 PTTG1 TRIP13 TTK	0
metaphase plate congression	13	CCNB1 CDCA5 CDCA8 CDT1 CENPE CEP55 FAM83D KIF18A KIF2C KIFC1 NDC80 NUF2 SPAG5	0.0002
T cell costimulation	10	BTLA CCL19 CCR7 CD5 CTLA4 ICOS LCK PDCD1 TNFRSF13C TNFSF13B	0.0002
chromosome separation	17	AURKB BLM BUB1 BUB1B CCNB1 CDT1 CENPE DLGAP5 ESPL1 MAD2L1 NCAPH NDC80 PLK1 PTTG1 TOP2A TRIP13 TTK	0.006
neural structure formation in CNS (R^2_0.8937, adjR^2_0.8805, p-value_0.0018) at T1N0			

related non-neural pathways	gene number	gene name	p-value
mitotic sister chromatid separation	14	AURKB BUB1 BUB1B CCNB1 CDT1 CENPE DLGAP5 ESPL1 MAD2L1 NDC80 PLK1 PTTG1 TRIP13 TTK	0
regulation of mitotic sister chromatid segregation	14	AURKB BUB1 BUB1B CCNB1 CDT1 CENPE DLGAP5 ESPL1 MAD2L1 NDC80 PLK1 PTTG1 TRIP13 TTK	0
T cell costimulation	10	BTLA CCL19 CCR7 CD5 CTLA4 ICOS LCK PDCD1 TNFRSF13C TNFSF13B	0
regulation of chromosome segregation	19	AURKB BUB1 BUB1B CCNB1 CDCA5 CDT1 CENPE DLGAP5 ESPL1 KIF2C MAD2L1 MKI67 NDC80 NEK2 PLK1 PTTG1 SPAG5 TRIP13 TTK	0
regulation of sister chromatid segregation	15	AURKB BUB1 BUB1B CCNB1 CDCA5 CDT1 CENPE DLGAP5 ESPL1 MAD2L1 NDC80 PLK1 PTTG1 TRIP13 TTK	0.0073
regulation of chromosome separation	14	AURKB BUB1 BUB1B CCNB1 CDT1 CENPE DLGAP5 ESPL1 MAD2L1 NDC80 PLK1 PTTG1 TRIP13 TTK	0.016
neuron differentiation (R^2_0.8673, adjR^2_0.8283, p-value_0.0003) at T1N0			
related non-neural pathways	gene number	gene name	p-value
negative regulation of mitotic nuclear division	11	AURKB BUB1 BUB1B CCNB1 CDT1 MAD2L1 NDC80 PLK1 PTTG1 TRIP13 TTK	0
T cell costimulation	10	BTLA CCL19 CCR7 CD5 CTLA4 ICOS LCK PDCD1 TNFRSF13C TNFSF13B	0
regulation of DNA-dependent DNA replication	7	BLM BRCA2 CDT1 E2F7 E2F8 TICRR TIMELESS	0
metaphase/anaphase transition of cell cycle	13	AURKB BUB1 BUB1B CCNB1 CDT1 CENPE DLGAP5 ESPL1 MAD2L1 NDC80 PLK1 TRIP13 TTK	0
positive regulation of mitotic cell cycle phase transition	11	CCNB1 CCNE1 CDC25A CDC25C CDC45 CDCA5 CDT1 DLGAP5 DTL ESPL1 UBE2C	0.0031
meiotic chromosome segregation	15	BLM BRIP1 BUB1 BUB1B CCNE1 CCNE2 ESPL1 NCAPH NUF2 PLK1 PTTG1 SMC4 TOP2A TRIP13 TTK	0

establishment of chromosome localization	14	CCNB1 CDCA5 CDCA8 CDT1 CENPE CEP55 DLGAP5 FAM83D KIF18A KIF2C KIFC1 NDC80 NUF2 SPAG5	0.0015
mitotic sister chromatid separation	14	AURKB BUB1 BUB1B CCNB1 CDT1 CENPE DLGAP5 ESPL1 MAD2L1 NDC80 PLK1 PTTG1 TRIP13 TTK	0
chromosome segregation	45	AURKB BLM BRIP1 BUB1 BUB1B CCNB1 CCNE1 CCNE2 CDCA5 CDCA8 CDT1 CENPE CENPW CEP55 DLGAP5 ESCO2 ESPL1 FAM83D HJURP KIF18A KIF18B KIF23 KIF2C KIFC1 MAD2L1 MKI67 NCAPG NCAPH NDC80 NEK2 NUF2 NUSAP1 OIP5 PLK1 PRC1 PTTG1 SKA1 SKA3 SMC4 SPAG5 SPC25 TOP2A TRIP13 TTK ZWINT	0.0003
regulation of mitotic sister chromatid segregation	14	AURKB BUB1 BUB1B CCNB1 CDT1 CENPE DLGAP5 ESPL1 MAD2L1 NDC80 PLK1 PTTG1 TRIP13 TTK	0.0008
regulation of chromosome separation	14	AURKB BUB1 BUB1B CCNB1 CDT1 CENPE DLGAP5 ESPL1 MAD2L1 NDC80 PLK1 PTTG1 TRIP13 TTK	0.004
mitotic cytokinesis	10	ANLN BRCA2 CDT1 CENPA CEP55 ESPL1 KIF20A KIF23 NUSAP1 PLK1	0.0044
regulation of sister chromatid segregation	15	AURKB BUB1 BUB1B CCNB1 CDCA5 CDT1 CENPE DLGAP5 ESPL1 MAD2L1 NDC80 PLK1 PTTG1 TRIP13 TTK	0.001
sister chromatid segregation	33	AURKB BUB1 BUB1B CCNB1 CDCA5 CDCA8 CDT1 CENPE CEP55 DLGAP5 ESCO2 ESPL1 KIF18A KIF18B KIF23 KIF2C KIFC1 MAD2L1 NCAPG NCAPH NDC80 NEK2 NUF2 NUSAP1 PLK1 PRC1 PTTG1 SMC4 SPAG5 TOP2A TRIP13 TTK ZWINT	0.0229
development of glial (R^2_0.8750, adjR^2_0.8566, p-value_0) at T1N0			
related non-neural pathways	gene number	gene name	p-value
negative regulation of nuclear division	11	AURKB BUB1 BUB1B CCNB1 CDT1 MAD2L1 NDC80 PLK1 PTTG1 TRIP13 TTK	0
regulation of mitotic sister chromatid separation	14	AURKB BUB1 BUB1B CCNB1 CDT1 CENPE DLGAP5 ESPL1 MAD2L1 NDC80 PLK1 PTTG1 TRIP13 TTK	0
negative regulation of mitotic nuclear division	11	AURKB BUB1 BUB1B CCNB1 CDT1 MAD2L1 NDC80 PLK1 PTTG1 TRIP13 TTK	0.0003

metaphase plate congression	13	CCNB1 CDCA5 CDCA8 CDT1 CENPE CEP55 FAM83D KIF18A KIF2C KIFC1 NDC80 NUF2 SPAG5	0
chromosome separation	17	AURKB BLM BUB1 BUB1B CCNB1 CDT1 CENPE DLGAP5 ESPL1 MAD2L1 NCAPH NDC80 PLK1 PTTG1 TOP2A TRIP13 TTK	0.0022
regulation of metaphase/anaphase transition of cell cycle	13	AURKB BUB1 BUB1B CCNB1 CDT1 CENPE DLGAP5 ESPL1 MAD2L1 NDC80 PLK1 TRIP13 TTK	0.0159
mitotic sister chromatid segregation	31	AURKB BUB1 BUB1B CCNB1 CDCA5 CDCA8 CDT1 CENPE CEP55 DLGAP5 ESPL1 KIF18A KIF18B KIF23 KIF2C KIFC1 MAD2L1 NCAPG NCAPH NDC80 NEK2 NUF2 NUSAP1 PLK1	0.021
lymphocyte costimulation	10	BTLA CCL19 CCR7 CD5 CTLA4 ICOS LCK PDCD1 TNFRSF13C TNFSF13B	0.0315
regulation of sister chromatid segregation	15	AURKB BUB1 BUB1B CCNB1 CDCA5 CDT1 CENPE DLGAP5 ESPL1 MAD2L1 NDC80 PLK1 PTTG1 TRIP13 TTK	0.0405
regulation of chromosome separation	14	AURKB BUB1 BUB1B CCNB1 CDT1 CENPE DLGAP5 ESPL1 MAD2L1 NDC80 PLK1 PTTG1 TRIP13 TTK	0.0405
neuron projection and apoptosis (R^2_0.6912, adjR^2_0.571, p-value_0.001) at T2N0			
related non-neural pathways	gene number	gene name	p-value
meiosis I cell cycle process	13	BLM CCNE1 CDC25A CDC25C ESPL1 FBXO5 MND1 PLK1 PTTG1 RAD21 RAD51 TOP2A TRIP13	0
cell cycle G2/M phase transition	23	AURKB BLM CCNA2 CCNB1 CCNB2 CDC25A CDC25C CDC6 CDC7 CHEK1 CLSPN DTL FBXO5 FOXM1 GTSE1 HMMR NEK2 ORC1 PLK1 PLK4 RAD21 TICRR TPX2	0
mitotic nuclear division	45	ANLN AURKB BUB1 BUB1B CCNA2 CCNB1 CCNB2 CCNE1 CDC25C CDC6 CDCA5 CDCA8 CDT1 CENPE CEP55 CHEK1 DLGAP5 DSCC1 ESPL1 FBXO5 KIF11 KIF18A KIF18B KIF23 KIF2C KIFC1 MAD2L1 MKI67 NCAPD2 NCAPG NCAPH NDC80 NEK2 NUSAP1 PLK1 PRC1 PTTG1 RAD21 SMC4 SPAG5 TPX2 TRIP13 TTK UBE2C ZWINT	0
positive regulation of mitotic cell cycle	17	CCNB1 CCNE1 CDC25A CDC25C CDC45 CDC6 CDC7 CDCA5 CDT1 DLGAP5 DTL ESPL1 FBXO5 MAD2L1 NDC80 NUSAP1 UBE2C	0

negative regulation of nuclear division	14	AURKB BUB1 BUB1B CCNB1 CDT1 CHEK1 FBXO5 MAD2L1 NDC80 PLK1 PTTG1 RAD21 TRIP13 TTK	0
homologous recombination	6	BLM MND1 RAD21 RAD51 TOP2A TRIP13	0
positive regulation of mitotic cell cycle phase transition	14	CCNB1 CCNE1 CDC25A CDC25C CDC45 CDC6 CDC7 CDCA5 CDT1 DLGAP5 DTL ESPL1 FBXO5 UBE2C	0.0007
organelle fission	51	ANLN AURKB BLM BUB1 BUB1B CCNA2 CCNB1 CCNB2 CCNE1 CDC25C CDC6 CDCA5 CDCA8 CDT1 CENPE CEP55 CHEK1 DLGAP5 DSCC1 ESPL1 FANCA FBXO5 KIF11 KIF18A KIF18B KIF23 KIF2C KIFC1 MAD2L1 MKI67 MND1 MTFR2 NCAPD2 NCAPG NCAPH NDC80 NEK2 NUSAP1 PLK1 PRC1 PTTG1 RAD21 RAD51 SMC4 SPAG5 TOP2A TPX2 TRIP13 TTK UBE2C ZWINT	0
regulation of interleukin-12 production	6	CCL19 CCR7 IDO1 IFNG LTB TIGIT	0.0008
reciprocal meiotic recombination	6	BLM MND1 RAD21 RAD51 TOP2A TRIP13	0.0003
complement activation, classical pathway	19	CR2 IGHG1 IGHG2 IGHM IGHV1-18 IGHV1-24 IGHV1-69-2 IGHV3-21 IGHV3-23 IGHV3-33 IGKV4-1 IGLC2 IGLC3 IGLV1-44 IGLV1-47 IGLV2-14 IGLV3-19 IGLV3-21 TRBC2	0.0003
negative regulation of cell cycle G2/M phase transition	10	AURKB BLM CDC6 CHEK1 CLSPN DTL ORC1 PLK1 RAD21 TICRR	0
mitotic spindle assembly	7	AURKB KIF11 KIF23 KIFC1 NEK2 PLK1 TPX2	0.0097
DNA integrity checkpoint	17	BLM CCNB1 CDC25C CDC45 CDC6 CDT1 CHEK1 CLSPN DTL E2F7 E2F8 GTSE1 ORC1 PLK1 TICRR TIMELESS TOP2A	0.0012
signal transduction involved in DNA damage checkpoint	8	CCNB1 CDC25C CHEK1 DTL E2F7 E2F8 GTSE1 PLK1	0.0012

chromosome segregation	47	AURKB BLM BUB1 BUB1B CCNB1 CCNE1 CDC6 CDCA5 CDCA8 CDT1 CENPE CENPN CENPW CEP55 DLGAP5 DSCC1 ESPL1 FAM83D FBXO5 HJURP KIF18A KIF18B KIF23 KIF2C KIFC1 MAD2L1 MKI67 NCAPD2 NCAPG NCAPH NDC80 NEK2 NUSAP1 OIP5 PLK1 PRC1 PTTG1 RAD21 SKA1 SKA3 SMC4 SPAG5 SPC25 TOP2A TRIP13 TTK ZWINT	0.0001
mitotic sister chromatid segregation	35	AURKB BUB1 BUB1B CCNB1 CDC6 CDCA5 CDCA8 CDT1 CENPE CEP55 DLGAP5 DSCC1 ESPL1 FBXO5 KIF18A KIF18B KIF23 KIF2C KIFC1 MAD2L1 NCAPD2 NCAPG NCAPH NDC80 NEK2 NUSAP1 PLK1 PRC1 PTTG1 RAD21 SMC4 SPAG5 TRIP13 TTK ZWINT	0.0012
cell cycle checkpoint	26	AURKB BLM BUB1 BUB1B CCNB1 CDC25C CDC45 CDC6 CDT1 CHEK1 CLSPN DTL E2F7 E2F8 GTSE1 MAD2L1 NDC80 ORC1 PLK1 TICRR TIMELESS TOP2A TRIP13 TTK ZWILCH ZWINT	0.0005
regulation of mitotic sister chromatid segregation	17	AURKB BUB1 BUB1B CCNB1 CDC6 CDT1 CENPE DLGAP5 ESPL1 FBXO5 MAD2L1 NDC80 PLK1 PTTG1 RAD21 TRIP13 TTK	0.0341
nuclear division	50	ANLN AURKB BLM BUB1 BUB1B CCNA2 CCNB1 CCNB2 CCNE1 CDC25C CDC6 CDCA5 CDCA8 CDT1 CENPE CEP55 CHEK1 DLGAP5 DSCC1 ESPL1 FANCA FBXO5 KIF11 KIF18A KIF18B KIF23 KIF2C KIFC1 MAD2L1 MKI67 MND1 NCAPD2 NCAPG NCAPH NDC80 NEK2 NUSAP1 PLK1 PRC1 PTTG1 RAD21 RAD51 SMC4 SPAG5 TOP2A TPX2 TRIP13 TTK UBE2C ZWINT	0.0062
regulation of mitotic nuclear division	29	ANLN AURKB BUB1 BUB1B CCNA2 CCNB1 CCNB2 CCNE1 CDC25C CDC6 CDCA5 CDT1 CENPE CHEK1 DLGAP5 ESPL1 FBXO5 KIF11 MAD2L1 MKI67 NDC80 NEK2 NUSAP1 PLK1 PTTG1 RAD21 TRIP13 TTK UBE2C	0.0003
nuclear chromosome segregation	39	AURKB BLM BUB1 BUB1B CCNB1 CCNE1 CDC6 CDCA5 CDCA8 CDT1 CENPE CEP55 DLGAP5 DSCC1 ESPL1 FAM83D FBXO5 KIF18A KIF18B KIF23 KIF2C KIFC1 MAD2L1 NCAPD2 NCAPG NCAPH NDC80 NEK2 NUSAP1 PLK1 PRC1 PTTG1 RAD21 SMC4 SPAG5 TOP2A TRIP13 TTK ZWINT	0.0003
DNA-dependent DNA replication	22	ALYREF BLM CCNE1 CDC45 CDC6 CDC7 CDT1 DSCC1 E2F7 E2F8 FBXO5 GINS1 MCM10 MCM2 MCM6 ORC1 ORC6 POLQ RAD51 TICRR TIMELESS WDHD1	0.0003

positive regulation of T cell differentiation	9	CCL19 CD27 FOXP3 IFNG IL12RB1 IL2RA LILRB4 TESPA1 ZAP70	0.0111
humoral immune response mediated by circulating immunoglobulin	19	CR2 IGHG1 IGHG2 IGHM IGHV1-18 IGHV1-24 IGHV1-69-2 IGHV3-21 IGHV3-23 IGHV3-33 IGKV4-1 IGLC2 IGLC3 IGLV1-44 IGLV1-47 IGLV2-14 IGLV3-19 IGLV3-21 TRBC2	0.0111
signal transduction involved in cell cycle checkpoint	8	CCNB1 CDC25C CHEK1 DTL E2F7 E2F8 GTSE1 PLK1	0.0111
G2/M transition of mitotic cell cycle	21	AURKB BLM CCNA2 CCNB1 CCNB2 CDC25A CDC25C CDC6 CDC7 CLSPN DTL FBXO5 FOXM1 HMMR NEK2 ORC1 PLK1 PLK4 RAD21 TICRR TPX2	0.0111
neural structure formation in CNS (R^2_0.8541, adjR^2_0.8243, p-value_0) at T2N0			
related non-neural pathways	gene number	gene name	p-value
adaptive immune response	89	BTLA CCL19 CD27 CD3D CD48 CD6 CD7 CD79A CR2 CRTAM CTLA4 FOXP3 GZMM IFNG IGHG1 IGHG2 IGHM IGHV1-18 IGHV1-24 IGHV1-69-2 IGHV3-21 IGHV3-23 IGHV3-33 IGKV1-8 IGKV4-1 IGLC2 IGLC3 IGLV1-44 IGLV1-47 IGLV2-14 IGLV3-19 IGLV3-21 IL12RB1 LAMP3 LILRB4 MYO1G SH2D1A SIT1 SLA2 SLAMF6 SLAMF7 TNFRSF13B TNFSF13B TRAC TRAT1 TRAV12-1 TRAV12-3 TRAV13-1 TRAV17 TRAV2 TRAV20 TRAV21 TRAV23DV6 TRAV25 TRAV26-1 TRAV27 TRAV29DV5 TRAV3 TRAV4 TRAV41 TRAV6 TRAV8-1 TRAV8-2 TRAV8-3 TRAV8-4 TRAV9-2 TRBC2 TRBV10-3 TRBV11-2 TRBV12-3 TRBV12-4 TRBV13 TRBV14 TRBV18 TRBV19 TRBV2 TRBV20-1 TRBV25-1 TRBV28 TRBV3-1 TRBV4-2 TRBV5-1 TRBV5-4 TRBV5-6 TRBV6-	0
regulation of mitotic nuclear division	29	ANLN AURKB BUB1 BUB1B CCNA2 CCNB1 CCNB2 CCNE1 CDC25C CDC6 CDCA5 CDT1 CENPE CHEK1 DLGAP5 ESPL1 FBXO5 KIF11 MAD2L1 MKI67 NDC80 NEK2 NUSAP1 PLK1 PTTG1 RAD21 TRIP13 TTK UBE2C	0
mitotic spindle assembly	7	AURKB KIF11 KIF23 KIFC1 NEK2 PLK1 TPX2	0
positive regulation of mitotic cell cycle phase transition	14	CCNB1 CCNE1 CDC25A CDC25C CDC45 CDC6 CDC7 CDCA5 CDT1 DLGAP5 DTL ESPL1 FBXO5 UBE2C	0.0002

regulation of mitotic sister chromatid segregation	17	AURKB BUB1 BUB1B CCNB1 CDC6 CDT1 CENPE DLGAP5 ESPL1 FBXO5 MAD2L1 NDC80 PLK1 PTTG1 RAD21 TRIP13 TTK	0
mitotic nuclear division	45	ANLN AURKB BUB1 BUB1B CCNA2 CCNB1 CCNB2 CCNE1 CDC25C CDC6 CDCA5 CDCA8 CDT1 CENPE CEP55 CHEK1 DLGAP5 DSCC1 ESPL1 FBXO5 KIF11 KIF18A KIF18B KIF23 KIF2C KIFC1 MAD2L1 MKI67 NCAPD2 NCAPG NCAPH NDC80 NEK2 NUSAP1 PLK1 PRC1 PTTG1 RAD21 SMC4 SPAG5 TPX2 TRIP13 TTK UBE2C ZWINT	0.0093
establishment of chromosome localization	13	CCNB1 CDCA5 CDCA8 CDT1 CENPE CEP55 DLGAP5 FAM83D KIF18A KIF2C KIFC1 NDC80 SPAG5	0.0016
regulation of mitotic metaphase/anaphase transition	16	AURKB BUB1 BUB1B CCNB1 CDC6 CDT1 CENPE DLGAP5 ESPL1 FBXO5 MAD2L1 NDC80 PLK1 RAD21 TRIP13 TTK	0.0221
metaphase/anaphase transition of cell cycle	16	AURKB BUB1 BUB1B CCNB1 CDC6 CDT1 CENPE DLGAP5 ESPL1 FBXO5 MAD2L1 NDC80 PLK1 RAD21 TRIP13 TTK	0
mitotic cytokinesis	9	ANLN CDT1 CENPA CEP55 ESPL1 KIF20A KIF23 NUSAP1 PLK1	0.028
negative regulation of mitotic cell cycle phase transition	21	AURKB BLM BUB1 BUB1B CCNB1 CDC25C CDC6 CDT1 CLSPN E2F7 E2F8 FBXO5 GTSE1 MAD2L1 NDC80 ORC1 PLK1 RAD21 TICRR TRIP13 TTK	0.0006
chromosome organization involved in meiotic cell cycle	8	BUB1 BUB1B CCNE1 NCAPD2 NCAPH RAD51 SMC4 TRIP13	0.0006
regulation of chromosome separation	17	AURKB BUB1 BUB1B CCNB1 CDC6 CDT1 CENPE DLGAP5 ESPL1 FBXO5 MAD2L1 NDC80 PLK1 PTTG1 RAD21 TRIP13 TTK	0.0004
cell cycle DNA replication	8	CDC45 CDC7 CDT1 E2F7 E2F8 FBXO5 GINS1 RAD51	0.023
regulation of mitotic sister chromatid separation	17	AURKB BUB1 BUB1B CCNB1 CDC6 CDT1 CENPE DLGAP5 ESPL1 FBXO5 MAD2L1 NDC80 PLK1 PTTG1 RAD21 TRIP13 TTK	0.023
regulation of G2/M transition of mitotic cell cycle	18	AURKB BLM CCNB1 CDC25A CDC25C CDC6 CDC7 CLSPN DTL FBXO5 HMMR NEK2 ORC1 PLK1 PLK4 RAD21 TICRR TPX2	0.0015

negative regulation of cell cycle phase transition	23	AURKB BLM BUB1 BUB1B CCNB1 CDC25C CDC6 CDT1 CHEK1 CLSPN DTL E2F7 E2F8 FBXO5 GTSE1 MAD2L1 NDC80 ORC1 PLK1 RAD21 TICRR TRIP13 TTK	0.0015
microtubule cytoskeleton organization involved in mitosis	16	AURKB CCNB1 CENPA CENPE ESPL1 KIF11 KIF23 KIFC1 NDC80 NEK2 NUSAP1 PLK1 PRC1 SPC25 TPX2 TTK	0.0015
regulation of DNA-dependent DNA replication	9	BLM CDC7 CDT1 DSCC1 E2F7 E2F8 FBXO5 TICRR TIMELESS	0.0079
negative regulation of cell cycle G2/M phase transition	10	AURKB BLM CDC6 CHEK1 CLSPN DTL ORC1 PLK1 RAD21 TICRR	0.0079
positive regulation of cell cycle phase transition	15	CCNB1 CCNE1 CDC25A CDC25C CDC45 CDC6 CDC7 CDCA5 CDT1 DLGAP5 DTL ESPL1 FAM83D FBXO5 UBE2C	0.0079
neuron differentiation (R^2_0.725, adjR^2_0.6268, p-value_0) at T2N0			
related non-neural pathways	gene number	gene name	p-value
regulation of DNA replication	12	BLM CCNA2 CDC6 CDC7 CDT1 DBF4 DSCC1 E2F7 E2F8 FBXO5 TICRR TIMELESS	0
signal transduction involved in cell cycle checkpoint	8	CCNB1 CDC25C CHEK1 DTL E2F7 E2F8 GTSE1 PLK1	0
negative regulation of mitotic cell cycle phase transition	21	AURKB BLM BUB1 BUB1B CCNB1 CDC25C CDC6 CDT1 CLSPN E2F7 E2F8 FBXO5 GTSE1 MAD2L1 NDC80 ORC1 PLK1 RAD21 TICRR TRIP13 TTK	0
regulation of mitotic nuclear division	29	ANLN AURKB BUB1 BUB1B CCNA2 CCNB1 CCNB2 CCNE1 CDC25C CDC6 CDCA5 CDT1 CENPE CHEK1 DLGAP5 ESPL1 FBXO5 KIF11 MAD2L1 MKI67 NDC80 NEK2 NUSAP1 PLK1 PTTG1 RAD21 TRIP13 TTK UBE2C	0
regulation of nuclear division	29	ANLN AURKB BUB1 BUB1B CCNA2 CCNB1 CCNB2 CCNE1 CDC25C CDC6 CDCA5 CDT1 CENPE CHEK1 DLGAP5 ESPL1 FBXO5 KIF11 MAD2L1 MKI67 NDC80 NEK2 NUSAP1 PLK1 PTTG1 RAD21 TRIP13 TTK UBE2C	0
regulation of mitotic sister chromatid segregation	17	AURKB BUB1 BUB1B CCNB1 CDC6 CDT1 CENPE DLGAP5 ESPL1 FBXO5 MAD2L1 NDC80 PLK1 PTTG1 RAD21 TRIP13 TTK	0

reciprocal meiotic recombination	6	BLM MND1 RAD21 RAD51 TOP2A TRIP13	0
meiotic chromosome segregation	12	BLM BUB1 BUB1B CCNE1 ESPL1 NCAPH PLK1 PTTG1 SMC4 TOP2A TRIP13 TTK	0.0002
meiosis I cell cycle process	13	BLM CCNE1 CDC25A CDC25C ESPL1 FBXO5 MND1 PLK1 PTTG1 RAD21 RAD51 TOP2A TRIP13	0
natural killer cell mediated immunity	7	CRTAM GZMB LAG3 NCR3 SH2D1A SLAMF6 SLAMF7	0
interleukin-12 production	6	CCL19 CCR7 IDO1 IFNG LTB TIGIT	0
negative regulation of cell cycle G2/M phase transition	10	AURKB BLM CDC6 CHEK1 CLSPN DTL ORC1 PLK1 RAD21 TICRR	0
antigen receptor-mediated signaling pathway	33	BLK CCR7 CD19 CD3D CD79A CTLA4 FCRL3 FOXP3 GBP1 HLA-DQA1 IGHG1 IGHG2 IGHM IGHV1-18 IGHV1-24 IGHV1-69-2 IGHV3-21 IGHV3-23 IGHV3-33 IGLC2 IGLC3 LCK LILRB4 SLA2 TESPA1 TRAC TRAT1 TRAV29DV5 TRAV8-4 TRBC2 TRBV12-3 TRBV7-9 ZAP70	0.0004
nuclear division	50	ANLN AURKB BLM BUB1 BUB1B CCNA2 CCNB1 CCNB2 CCNE1 CDC25C CDC6 CDCA5 CDCA8 CDT1 CENPE CEP55 CHEK1 DLGAP5 DSCC1 ESPL1 FANCA FBXO5 KIF11 KIF18A KIF18B KIF23 KIF2C KIFC1 MAD2L1 MKI67 MND1 NCAPD2 NCAPG NCAPH NDC80 NEK2 NUSAP1 PLK1 PRC1 PTTG1 RAD21 RAD51 SMC4 SPAG5 TOP2A TPX2 TRIP13 TTK UBE2C ZWINT	0.0004
cell cycle DNA replication	8	CDC45 CDC7 CDT1 E2F7 E2F8 FBXO5 GINS1 RAD51	0
metaphase/anaphase transition of cell cycle	16	AURKB BUB1 BUB1B CCNB1 CDC6 CDT1 CENPE DLGAP5 ESPL1 FBXO5 MAD2L1 NDC80 PLK1 RAD21 TRIP13 TTK	0.0195

sister chromatid segregation	36	AURKB BUB1 BUB1B CCNB1 CDC6 CDCA5 CDCA8 CDT1 CENPE CEP55 DLGAP5 DSCC1 ESPL1 FBXO5 KIF18A KIF18B KIF23 KIF2C KIFC1 MAD2L1 NCAPD2 NCAPG NCAPH NDC80 NEK2 NUSAP1 PLK1 PRC1 PTTG1 RAD21 SMC4 SPAG5 TOP2A TRIP13 TTK ZWINT	0.0441
chromosome segregation	47	AURKB BLM BUB1 BUB1B CCNB1 CCNE1 CDC6 CDCA5 CDCA8 CDT1 CENPE CENPN CENPW CEP55 DLGAP5 DSCC1 ESPL1 FAM83D FBXO5 HJURP KIF18A KIF18B KIF23 KIF2C KIFC1 MAD2L1 MKI67 NCAPD2 NCAPG NCAPH NDC80 NEK2 NUSAP1 OIP5 PLK1 PRC1 PTTG1 RAD21 SKA1 SKA3 SMC4 SPAG5 SPC25 TOP2A TRIP13 TTK ZWINT	0.0013
negative regulation of leukocyte apoptotic process	7	AURKB BLM CCL19 CCR5 CCR7 CD27 IDO1	0.0013
nuclear chromosome segregation	39	AURKB BLM BUB1 BUB1B CCNB1 CCNE1 CDC6 CDCA5 CDCA8 CDT1 CENPE CEP55 DLGAP5 DSCC1 ESPL1 FAM83D FBXO5 KIF18A KIF18B KIF23 KIF2C KIFC1 MAD2L1 NCAPD2 NCAPG NCAPH NDC80 NEK2 NUSAP1 PLK1 PRC1 PTTG1 RAD21 SMC4 SPAG5 TOP2A TRIP13 TTK ZWINT	0.0013
regulation of sister chromatid segregation	18	AURKB BUB1 BUB1B CCNB1 CDC6 CDCA5 CDT1 CENPE DLGAP5 ESPL1 FBXO5 MAD2L1 NDC80 PLK1 PTTG1 RAD21 TRIP13 TTK	0.0021
mitotic sister chromatid separation	17	AURKB BUB1 BUB1B CCNB1 CDC6 CDT1 CENPE DLGAP5 ESPL1 FBXO5 MAD2L1 NDC80 PLK1 PTTG1 RAD21 TRIP13 TTK	0.0153
neuron projection and apoptosis (R^2_0.7071, adjR^2_0.6257, p-value_0.001) at T3N0			
related non-neural pathways	gene number	gene name	p-value
regulation of mitotic sister chromatid segregation	16	AURKB BUB1 BUB1B CCNB1 CDC6 CDT1 CENPE DLGAP5 ESPL1 MAD2L1 NDC80 PLK1 PTTG1 RAD21 TRIP13 TTK	0
chromosome separation	21	AURKB BLM BUB1 BUB1B CCNB1 CDC6 CDT1 CENPE DLGAP5 EME1 EME2 ESPL1 MAD2L1 NCAPH NDC80 PLK1 PTTG1 RAD21 TOP2A TRIP13 TTK	0
regulation of mitotic sister chromatid separation	16	AURKB BUB1 BUB1B CCNB1 CDC6 CDT1 CENPE DLGAP5 ESPL1 MAD2L1 NDC80 PLK1 PTTG1 RAD21 TRIP13 TTK	0

regulation of metaphase/anaphase transition of cell cycle	15	AURKB BUB1 BUB1B CCNB1 CDC6 CDT1 CENPE DLGAP5 ESPL1 MAD2L1 NDC80 PLK1 RAD21 TRIP13 TTK	0.0006
regulation of mitotic metaphase/anaphase transition	15	AURKB BUB1 BUB1B CCNB1 CDC6 CDT1 CENPE DLGAP5 ESPL1 MAD2L1 NDC80 PLK1 RAD21 TRIP13 TTK	0.0006
neural structure formation in CNS (R^2_0.8265, adjR^2_0.529, p-value_0.002) at T3N0			
related non-neural pathways	gene number	gene name	p-value
sister chromatid segregation	36	AURKB BUB1 BUB1B CCNB1 CDC6 CDCA5 CDCA8 CDT1 CENPE CEP55 DLGAP5 DSCC1 ESPL1 KIF18A KIF18B KIF23 KIF2C KIFC1 KNSTRN MAD2L1 NCAPG NCAPH NDC80 NEK2 NUSAP1 PLK1 PRC1 PSRC1 PTTG1 RAD21 SMC4 SPAG5 TOP2A TRIP13 TTK ZWINT	0
metaphase/anaphase transition of mitotic cell cycle	15	AURKB BUB1 BUB1B CCNB1 CDC6 CDT1 CENPE DLGAP5 ESPL1 MAD2L1 NDC80 PLK1 RAD21 TRIP13 TTK	0
meiotic chromosome segregation	16	BLM BRIP1 BUB1 BUB1B CCNE1 CCNE2 EME1 EME2 ESPL1 NCAPH PLK1 PTTG1 SMC4 TOP2A TRIP13 TTK	0.0066
metaphase plate congression	13	CCNB1 CDCA5 CDCA8 CDT1 CENPE CEP55 FAM83D KIF18A KIF2C KIFC1 NDC80 PSRC1 SPAG5	0
regulation of chromosome segregation	22	AURKB BUB1 BUB1B CCNB1 CDC6 CDCA5 CDT1 CENPE DLGAP5 ESPL1 KIF2C KNSTRN MAD2L1 MKI67 NDC80 NEK2 PLK1 PTTG1 RAD21 SPAG5 TRIP13 TTK	0.0003
chromosome localization	14	CCNB1 CDCA5 CDCA8 CDT1 CENPE CEP55 DLGAP5 FAM83D KIF18A KIF2C KIFC1 NDC80 PSRC1 SPAG5	0.0005
chromosome separation	21	AURKB BLM BUB1 BUB1B CCNB1 CDC6 CDT1 CENPE DLGAP5 EME1 EME2 ESPL1 MAD2L1 NCAPH NDC80 PLK1 PTTG1 RAD21 TOP2A TRIP13 TTK	0
homologous recombination	10	BLM BRIP1 EME1 EME2 MND1 RAD21 RAD51 RAD54B TOP2A TRIP13	0.0011
metaphase/anaphase transition of cell cycle	15	AURKB BUB1 BUB1B CCNB1 CDC6 CDT1 CENPE DLGAP5 ESPL1 MAD2L1 NDC80 PLK1 RAD21 TRIP13 TTK	0.0024
neuron differentiation (R^2_0.809, adjR^2_0.6621, p-value_0.0009) at T3N0			

related non-neural pathways	gene number	gene name	p-value
homologous recombination	10	BLM BRIP1 EME1 EME2 MND1 RAD21 RAD51 RAD54B TOP2A TRIP13	0
metaphase/anaphase transition of mitotic cell cycle	15	AURKB BUB1 BUB1B CCNB1 CDC6 CDT1 CENPE DLGAP5 ESPL1 MAD2L1 NDC80 PLK1 RAD21 TRIP13 TTK	0
sister chromatid segregation	36	AURKB BUB1 BUB1B CCNB1 CDC6 CDCA5 CDCA8 CDT1 CENPE CEP55 DLGAP5 DSCC1 ESPL1 KIF18A KIF18B KIF23 KIF2C KIFC1 KNSTRN MAD2L1 NCAPG NCAPH NDC80 NEK2 NUSAP1 PLK1 PRC1 PSRC1 PTTG1 RAD21 SMC4 SPAG5 TOP2A TRIP13 TTK ZWINT	0.0014
regulation of chromosome segregation	22	AURKB BUB1 BUB1B CCNB1 CDC6 CDCA5 CDT1 CENPE DLGAP5 ESPL1 KIF2C KNSTRN MAD2L1 MKI67 NDC80 NEK2 PLK1 PTTG1 RAD21 SPAG5 TRIP13 TTK	0.0109
regulation of sister chromatid segregation	17	AURKB BUB1 BUB1B CCNB1 CDC6 CDCA5 CDT1 CENPE DLGAP5 ESPL1 MAD2L1 NDC80 PLK1 PTTG1 RAD21 TRIP13 TTK	0
chromosome separation	21	AURKB BLM BUB1 BUB1B CCNB1 CDC6 CDT1 CENPE DLGAP5 EME1 EME2 ESPL1 MAD2L1 NCAPH NDC80 PLK1 PTTG1 RAD21 TOP2A TRIP13 TTK	0.0378
mitotic sister chromatid segregation	35	AURKB BUB1 BUB1B CCNB1 CDC6 CDCA5 CDCA8 CDT1 CENPE CEP55 DLGAP5 DSCC1 ESPL1 KIF18A KIF18B KIF23 KIF2C KIFC1 KNSTRN MAD2L1 NCAPG NCAPH NDC80 NEK2 NUSAP1 PLK1 PRC1 PSRC1 PTTG1 RAD21 SMC4 SPAG5 TRIP13 TTK ZWINT	0.0277
neuron projection and apoptosis (R^2_0.7794, adjR^2_0.7324, p-value_0) at N1			
related non-neural pathways	gene number	gene name	p-value
metaphase plate congression	12	CCNB1 CDCA5 CDCA8 CENPE CEP55 FAM83D KIF18A KIF2C KIFC1 NDC80 NUF2 SPAG5	0
sister chromatid cohesion	7	BUB1 BUB1B CDCA5 DSCC1 ESCO2 ESPL1 PLK1	0
regulation of B cell activation	24	CD27 CTLA4 FCRL3 FOXP3 IGHG1 IGHG2 IGHV1-18 IGHV1-24 IGHV1-69 IGHV1-69-2 IGHV2-26 IGHV3-21 IGHV3-23 IGHV3-33 IGHV3-48 IGHV3-66 IGHV4-31 IGHV4-34	0

chromosome localization	13	CCNB1 CDCA5 CDCA8 CENPE CEP55 DLGAP5 FAM83D KIF18A KIF2C KIFC1 NDC80 NUF2 SPAG5	0.0055
microtubule cytoskeleton organization involved in mitosis	16	AURKB CCNB1 CENPA CENPE ESPL1 KIF11 KIF23 KIFC1 NDC80 NEK2 NUF2 NUSAP1 PLK1 PRC1 TPX2 TTK	0
phagocytosis, recognition	18	IGHG1 IGHG2 IGHV1-18 IGHV1-24 IGHV1-69 IGHV1-69-2 IGHV2-26 IGHV3-21 IGHV3-23 IGHV3-33 IGHV3-48 IGHV3-66 IGHV4-31 IGHV4-34 IGHV4-39 IGLC2 IGLC3 TRBC2	0.0007
mitotic cell cycle checkpoint	21	AURKB BLM BUB1 BUB1B CCNB1 CDC25C CLSPN E2F7 E2F8 GTSE1 KNTC1 MAD2L1 NDC80 ORC1 PLK1 TICRR TOP2A TRIP13 TTK ZWILCH ZWINT	0
regulation of acute inflammatory response	17	CCR7 CD19 IGHG1 IGHG2 IGHV1-69 IGHV3-23 IGHV3-33 IGHV3-48 IGHV4-34 IGHV4-39 IGKV3-11 IGKV4-1 IGLV1-40 IGLV1-44 IGLV1-47 IGLV3-1 IGLV3-21	0.033
cell cycle checkpoint	25	AURKB BLM BUB1 BUB1B CCNB1 CDC25C CDC45 CHEK1 CLSPN DTL E2F7 E2F8 GTSE1 KNTC1 MAD2L1 NDC80 ORC1 PLK1 TICRR TIMELESS TOP2A TRIP13 TTK ZWILCH	0.033
nuclear division	47	ANLN AURKB BLM BUB1 BUB1B CCNA2 CCNB1 CCNB2 CCNE2 CDC25C CDCA5 CDCA8 CENPE CEP55 CHEK1 DLGAP5 DSCC1 ESPL1 KIF11 KIF18A KIF18B KIF23 KIF2C KIFC1 KNTC1 MAD2L1 MKI67 MND1 NCAPG NCAPH NDC80 NEK2 NUF2 NUSAP1 PKMYT1 PLK1 PRC1 PTTG1 RAD51 SMC4 SPAG5 TOP2A TPX2 TRIP13 TTK UBE2C ZWINT	0.0002
regulation of complement activation	16	CD19 IGHG1 IGHG2 IGHV1-69 IGHV3-23 IGHV3-33 IGHV3-48 IGHV4-34 IGHV4-39 IGKV3-11 IGKV4-1 IGLV1-40 IGLV1-44 IGLV1-47 IGLV3-1 IGLV3-21	0.0006
T cell costimulation	6	CCL19 CCR7 CD5 CTLA4 ICOS PDCD1	0.0006
collagen fibril organization	6	ADAMTS14 AEBP1 COL12A1 COL1A2 COL5A1 COL5A2	0.0002
ATP-dependent chromatin remodeling	8	CENPA CENPI CENPL CENPN CENPO CENPW HJURP OIP5	0.0315
plasma membrane invagination	19	AURKB IGHG1 IGHG2 IGHV1-18 IGHV1-24 IGHV1-69 IGHV1-69-2 IGHV2-26 IGHV3-21 IGHV3-23 IGHV3-33 IGHV3-48 IGHV3-66 IGHV4-31 IGHV4-34 IGHV4-39 IGLC2 IGLC3 TRBC2	0.0313

regulation of humoral immune response	17	CCR7 CD19 IGHG1 IGHG2 IGHV1-69 IGHV3-23 IGHV3-33 IGHV3-48 IGHV4-34 IGHV4-39 IGKV3-11 IGKV4-1 IGLV1-40 IGLV1-44 IGLV1-47 IGLV3-1 IGLV3-21	0.0313
nuclear chromosome segregation	36	AURKB BLM BUB1 BUB1B CCNB1 CCNE2 CDCA5 CDCA8 CENPE CEP55 DLGAP5 DSCC1 ESCO2 ESPL1 FAM83D KIF18A KIF18B KIF23 KIF2C KIFC1 MAD2L1 NCAPG NCAPH NDC80 NEK2 NUF2 NUSAP1 PLK1 PRC1 PTTG1 SMC4 SPAG5 TOP2A TRIP13 TTK ZWINT	0.0109
positive regulation of B cell activation	20	CD27 FCRL3 IGHG1 IGHG2 IGHV1-18 IGHV1-24 IGHV1-69 IGHV1-69-2 IGHV2-26 IGHV3-21 IGHV3-23 IGHV3-33 IGHV3-48 IGHV3-66 IGHV4-31 IGHV4-34 IGHV4-39 IGLC2 IGLC3 TRBC2	0.0016
membrane invagination	19	AURKB IGHG1 IGHG2 IGHV1-18 IGHV1-24 IGHV1-69 IGHV1-69-2 IGHV2-26 IGHV3-21 IGHV3-23 IGHV3-33 IGHV3-48 IGHV3-66 IGHV4-31 IGHV4-34 IGHV4-39 IGLC2 IGLC3 TRBC2	0.0016
negative regulation of leukocyte apoptotic process	6	AURKB BLM CCL19 CCR7 CD27 IDO1	0.0225
regulation of mitotic sister chromatid separation	13	AURKB BUB1 BUB1B CCNB1 CENPE DLGAP5 ESPL1 MAD2L1 NDC80 PLK1 PTTG1 TRIP13 TTK	0.0055
metaphase/anaphase transition of mitotic cell cycle	12	AURKB BUB1 BUB1B CCNB1 CENPE DLGAP5 ESPL1 MAD2L1 NDC80 PLK1 TRIP13 TTK	0.0055
regulation of mitotic metaphase/anaphase transition	12	AURKB BUB1 BUB1B CCNB1 CENPE DLGAP5 ESPL1 MAD2L1 NDC80 PLK1 TRIP13 TTK	0.0055
regulation of metaphase/anaphase transition of cell cycle	12	AURKB BUB1 BUB1B CCNB1 CENPE DLGAP5 ESPL1 MAD2L1 NDC80 PLK1 TRIP13 TTK	0.0055
positive regulation of cell cycle phase transition	10	CCNB1 CDC25A CDC25C CDC45 CDCA5 DLGAP5 DTL ESPL1 FAM83D UBE2C	0.0322
negative regulation of nuclear division	11	AURKB BUB1 BUB1B CCNB1 CHEK1 MAD2L1 NDC80 PLK1 PTTG1 TRIP13 TTK	0.0211
mitotic sister chromatid segregation	31	AURKB BUB1 BUB1B CCNB1 CDCA5 CDCA8 CENPE CEP55 DLGAP5 DSCC1 ESPL1 KIF18A KIF18B KIF23 KIF2C KIFC1 MAD2L1 NCAPG NCAPH NDC80 NEK2 NUF2 NUSAP1 PLK1 PRC1 PTTG1 SMC4 SPAG5 TRIP13 TTK ZWINT	0.0211

mitotic nuclear division	43	ANLN AURKB BUB1 BUB1B CCNA2 CCNB1 CCNB2 CCNE2 CDC25C CDCA5 CDCA8 CENPE CEP55 CHEK1 DLGAP5 DSCC1 ESPL1 KIF11 KIF18A KIF18B KIF23 KIF2C KIFC1 KNTC1 MAD2L1 MKI67 NCAPG NCAPH NDC80 NEK2 NUF2 NUSAP1 PKMYT1 PLK1 PRC1 PTTG1 SMC4 SPAG5 TPX2 TRIP13 TTK UBE2C ZWINT	0.0329
regulation of mitotic nuclear division	27	ANLN AURKB BUB1 BUB1B CCNA2 CCNB1 CCNB2 CCNE2 CDC25C CDCA5 CENPE CHEK1 DLGAP5 ESPL1 KIF11 KNTC1 MAD2L1 MKI67 NDC80 NEK2 NUSAP1 PKMYT1 PLK1 PTTG1 TRIP13 TTK UBE2C	0.0329
humoral immune response mediated by circulating immunoglobulin	25	IGHG1 IGHG2 IGHV1-18 IGHV1-24 IGHV1-69 IGHV1-69-2 IGHV2-26 IGHV3-21 IGHV3-23 IGHV3-33 IGHV3-48 IGHV3-66 IGHV4-31 IGHV4-34 IGHV4-39 IGKV3-11 IGKV4-1 IGLC2 IGLC3 IGLV1-40 IGLV1-44 IGLV1-47 IGLV3-1 IGLV3-21 TRBC2	0.0329
neuron differentiation (R^2_0.7949, adjR^2_0.7380, p-value_0.043) at N1			
related non-neural pathways	gene number	gene name	p-value
B cell receptor signaling pathway	23	BLK CD19 CD79A CTLA4 FCRL3 IGHG1 IGHG2 IGHV1-18 IGHV1-24 IGHV1-69 IGHV1-69-2 IGHV2-26 IGHV3-21 IGHV3-23 IGHV3-33 IGHV3-48 IGHV3-66 IGHV4-31 IGHV4-34 IGHV4-39 IGLC2 IGLC3 TRBC2	0
Fc receptor mediated stimulatory signaling pathway	15	IGHG1 IGHG2 IGHV1-69 IGHV3-23 IGHV3-33 IGHV3-48 IGHV4-34 IGHV4-39 IGKV3-11 IGKV4-1 IGLV1-40 IGLV1-44 IGLV1-47 IGLV3-1 IGLV3-21	0
immune response-regulating cell surface receptor signaling pathway involved in phagocytosis	15	IGHG1 IGHG2 IGHV1-69 IGHV3-23 IGHV3-33 IGHV3-48 IGHV4-34 IGHV4-39 IGKV3-11 IGKV4-1 IGLV1-40 IGLV1-44 IGLV1-47 IGLV3-1 IGLV3-21	0
signal transduction involved in DNA damage checkpoint	8	CCNB1 CDC25C CHEK1 DTL E2F7 E2F8 GTSE1 PLK1	0
regulation of mitotic nuclear division	27	ANLN AURKB BUB1 BUB1B CCNA2 CCNB1 CCNB2 CCNE2 CDC25C CDCA5 CENPE CHEK1 DLGAP5 ESPL1 KIF11 KNTC1 MAD2L1 MKI67 NDC80 NEK2 NUSAP1 PKMYT1 PLK1 PTTG1 TRIP13 TTK UBE2C	0.0016
regulation of DNA-dependent DNA replication	6	BLM DSCC1 E2F7 E2F8 TICRR TIMELESS	0

membrane invagination	19	AURKB IGHG1 IGHG2 IGHV1-18 IGHV1-24 IGHV1-69 IGHV1-69-2 IGHV2-26 IGHV3-21 IGHV3-23 IGHV3-33 IGHV3-48 IGHV3-66 IGHV4-31 IGHV4-34 IGHV4-39 IGLC2 IGLC3 TRBC2	0
regulation of cyclin-dependent protein kinase activity	10	BLM CCNA2 CCNB1 CCNB2 CCNE2 CDC25A CDC25C CDKN3 PKMYT1 PLK1	0.0005
regulation of mitotic sister chromatid segregation	13	AURKB BUB1 BUB1B CCNB1 CENPE DLGAP5 ESPL1 MAD2L1 NDC80 PLK1 PTTG1 TRIP13 TTK	0
regulation of protein activation cascade	16	CD19 IGHG1 IGHG2 IGHV1-69 IGHV3-23 IGHV3-33 IGHV3-48 IGHV4-34 IGHV4-39 IGKV3-11 IGKV4-1 IGLV1-40 IGLV1-44 IGLV1-47 IGLV3-1 IGLV3-21	0
phagocytosis, engulfment	18	IGHG1 IGHG2 IGHV1-18 IGHV1-24 IGHV1-69 IGHV1-69-2 IGHV2-26 IGHV3-21 IGHV3-23 IGHV3-33 IGHV3-48 IGHV3-66 IGHV4-31 IGHV4-34 IGHV4-39 IGLC2 IGLC3 TRBC2	0
mitotic nuclear division	43	ANLN AURKB BUB1 BUB1B CCNA2 CCNB1 CCNB2 CCNE2 CDC25C CDCA5 CDCA8 CENPE CEP55 CHEK1 DLGAP5 DSCC1 ESPL1 KIF11 KIF18A KIF18B KIF23 KIF2C KIFC1 KNTC1 MAD2L1 MKI67 NCAPG NCAPH NDC80 NEK2 NUF2 NUSAP1 PKMYT1 PLK1 PRC1 PTTG1 SMC4 SPAG5 TPX2 TRIP13 TTK UBE2C ZWINT	0.0005
DNA-dependent DNA replication	16	BLM CCNE2 CDC45 DSCC1 E2F7 E2F8 GINS1 MCM10 MCM6 ORC1 ORC6 POLQ RAD51 TICRR TIMELESS WDHD1	0.0362
negative regulation of cell cycle G2/M phase transition	8	AURKB BLM CHEK1 CLSPN DTL ORC1 PLK1 TICRR	0.0312
complement activation	26	CD19 IGHG1 IGHG2 IGHV1-18 IGHV1-24 IGHV1-69 IGHV1-69-2 IGHV2-26 IGHV3-21 IGHV3-23 IGHV3-33 IGHV3-48 IGHV3-66 IGHV4-31 IGHV4-34 IGHV4-39 IGKV3-11 IGKV4-1 IGLC2 IGLC3 IGLV1-40 IGLV1-44 IGLV1-47 IGLV3-1 IGLV3-21 TRBC2	0.0065
signal transduction involved in DNA integrity checkpoint	8	CCNB1 CDC25C CHEK1 DTL E2F7 E2F8 GTSE1 PLK1	0
Fc-gamma receptor signaling pathway	15	IGHG1 IGHG2 IGHV1-69 IGHV3-23 IGHV3-33 IGHV3-48 IGHV4-34 IGHV4-39 IGKV3-11 IGKV4-1 IGLV1-40 IGLV1-44 IGLV1-47 IGLV3-1 IGLV3-21	0.0037

chromosome separation	16	AURKB BLM BUB1 BUB1B CCNB1 CENPE DLGAP5 ESPL1 MAD2L1 NCAPH NDC80 PLK1 PTTG1 TOP2A TRIP13 TTK	0
metaphase plate congression	12	CCNB1 CDCA5 CDCA8 CENPE CEP55 FAM83D KIF18A KIF2C KIFC1 NDC80 NUF2 SPAG5	0
regulation of chromosome separation	13	AURKB BUB1 BUB1B CCNB1 CENPE DLGAP5 ESPL1 MAD2L1 NDC80 PLK1 PTTG1 TRIP13 TTK	0
Fc-gamma receptor signaling pathway involved in phagocytosis	15	IGHG1 IGHG2 IGHV1-69 IGHV3-23 IGHV3-33 IGHV3-48 IGHV4-34 IGHV4-39 IGKV3-11 IGKV4-1 IGLV1-40 IGLV1-44 IGLV1-47 IGLV3-1 IGLV3-21	0.0351
sister chromatid segregation	33	AURKB BUB1 BUB1B CCNB1 CDCA5 CDCA8 CENPE CEP55 DLGAP5 DSCC1 ESCO2 ESPL1 KIF18A KIF18B KIF23 KIF2C KIFC1 MAD2L1 NCAPG NCAPH NDC80 NEK2 NUF2 NUSAP1 PLK1 PRC1 PTTG1 SMC4 SPAG5 TOP2A TRIP13 TTK ZWINT	0.0364
metaphase/anaphase transition of cell cycle	12	AURKB BUB1 BUB1B CCNB1 CENPE DLGAP5 ESPL1 MAD2L1 NDC80 PLK1 TRIP13 TTK	0.0019
negative regulation of mitotic nuclear division	11	AURKB BUB1 BUB1B CCNB1 CHEK1 MAD2L1 NDC80 PLK1 PTTG1 TRIP13 TTK	0.0248
development of glial (R^2_0.8496, adjR^2_0.827, p-value_0) at N1			
related non-neural pathways	gene number	gene name	p-value
collagen fibril organization	6	ADAMTS14 AEBP1 COL12A1 COL1A2 COL5A1 COL5A2	0
negative regulation of cell cycle G2/M phase transition	8	AURKB BLM CHEK1 CLSPN DTL ORC1 PLK1 TICRR	0
chromosome separation	16	AURKB BLM BUB1 BUB1B CCNB1 CENPE DLGAP5 ESPL1 MAD2L1 NCAPH NDC80 PLK1 PTTG1 TOP2A TRIP13 TTK	0
mitotic nuclear division	43	ANLN AURKB BUB1 BUB1B CCNA2 CCNB1 CCNB2 CCNE2 CDC25C CDCA5 CDCA8 CENPE CEP55 CHEK1 DLGAP5 DSCC1 ESPL1 KIF11 KIF18A KIF18B KIF23 KIF2C KIFC1 KNTC1 MAD2L1 MKI67 NCAPG NCAPH NDC80 NEK2 NUF2 NUSAP1 PKMYT1 PLK1 PRC1 PTTG1 SMC4 SPAG5 TPX2 TRIP13 TTK UBE2C ZWINT	0.0018

negative regulation of leukocyte apoptotic process	6	AURKB BLM CCL19 CCR7 CD27 IDO1	0.0015
regulation of sister chromatid segregation	14	AURKB BUB1 BUB1B CCNB1 CDCA5 CENPE DLGAP5 ESPL1 MAD2L1 NDC80 PLK1 PTTG1 TRIP13 TTK	0.0008
plasma membrane invagination	19	AURKB IGHG1 IGHG2 IGHV1-18 IGHV1-24 IGHV1-69 IGHV1-69-2 IGHV2-26 IGHV3-21 IGHV3-23 IGHV3-33 IGHV3-48 IGHV3-66 IGHV4-31 IGHV4-34 IGHV4-39 IGLC2 IGLC3 TRBC2	0
mitotic spindle organization	13	AURKB CCNB1 CENPE KIF11 KIF23 KIFC1 NDC80 NEK2 NUF2 PLK1 PRC1 TPX2 TTK	0.0076
mitotic sister chromatid separation	13	AURKB BUB1 BUB1B CCNB1 CENPE DLGAP5 ESPL1 MAD2L1 NDC80 PLK1 PTTG1 TRIP13 TTK	0
regulation of chromosome separation	13	AURKB BUB1 BUB1B CCNB1 CENPE DLGAP5 ESPL1 MAD2L1 NDC80 PLK1 PTTG1 TRIP13 TTK	0.008
regulation of protein activation cascade	16	CD19 IGHG1 IGHG2 IGHV1-69 IGHV3-23 IGHV3-33 IGHV3-48 IGHV4-34 IGHV4-39 IGKV3-11 IGKV4-1 IGLV1-40 IGLV1-44 IGLV1-47 IGLV3-1 IGLV3-21	0
phagocytosis, engulfment	18	IGHG1 IGHG2 IGHV1-18 IGHV1-24 IGHV1-69 IGHV1-69-2 IGHV2-26 IGHV3-21 IGHV3-23 IGHV3-33 IGHV3-48 IGHV3-66 IGHV4-31 IGHV4-34 IGHV4-39 IGLC2 IGLC3 TRBC2	0.0182
regulation of mitotic sister chromatid separation	13	AURKB BUB1 BUB1B CCNB1 CENPE DLGAP5 ESPL1 MAD2L1 NDC80 PLK1 PTTG1 TRIP13 TTK	0.0028
humoral immune response mediated by circulating immunoglobulin	25	IGHG1 IGHG2 IGHV1-18 IGHV1-24 IGHV1-69 IGHV1-69-2 IGHV2-26 IGHV3-21 IGHV3-23 IGHV3-33 IGHV3-48 IGHV3-66 IGHV4-31 IGHV4-34 IGHV4-39 IGKV3-11 IGKV4-1 IGLC2	0.0016
chromosome localization	13	CCNB1 CDCA5 CDCA8 CENPE CEP55 DLGAP5 FAM83D KIF18A KIF2C KIFC1 NDC80 NUF2 SPAG5	0.0071
chromosome segregation	43	AURKB BLM BUB1 BUB1B CCNB1 CCNE2 CDCA5 CDCA8 CENPE CENPN CENPW CEP55 DLGAP5 DSCC1 ESCO2 ESPL1 FAM83D HJURP KIF18A KIF18B KIF23 KIF2C KIFC1 MAD2L1 MKI67 NCAPG NCAPH NDC80 NEK2 NUF2 NUSAP1 OIP5 PLK1 PRC1 PTTG1 SKA1 SKA3 SMC4 SPAG5 TOP2A TRIP13 TTK ZWINT	0.0244

complement activation, classical pathway	25	IGHG1 IGHG2 IGHV1-18 IGHV1-24 IGHV1-69 IGHV1-69-2 IGHV2-26 IGHV3-21 IGHV3-23 IGHV3-33 IGHV3-48 IGHV3-66 IGHV4-31 IGHV4-34 IGHV4-39 IGKV3-11 IGKV4-1 IGLC2 IGLC3 IGLV1-40 IGLV1-44 IGLV1-47 IGLV3-1 IGLV3-21 TRBC2	0.0068
B cell receptor signaling pathway	23	BLK CD19 CD79A CTLA4 FCRL3 IGHG1 IGHG2 IGHV1-18 IGHV1-24 IGHV1-69 IGHV1-69-2 IGHV2-26 IGHV3-21 IGHV3-23 IGHV3-33 IGHV3-48 IGHV3-66 IGHV4-31 IGHV4-34 IGHV4-39 IGLC2 IGLC3 TRBC2	0.0068
neuron projection and apoptosis (R^2_0.861, adjR^2_0.8222, p-value_0.0009) at N2			
related non-neural pathways	gene number	gene name	p-value
chromosome organization	55	ASF1B ATAD2 AURKB BLM BRIP1 BUB1 BUB1B CCNA2 CCNB1 CDC45 CDCA5 CDCA8 CENPA CENPE CENPI CENPL CENPN CEP55 DLGAP5 DNA2 ESCO2 ESPL1 EXO1 FOXP3 GEN1 GINS1 HIST1H2BO HJURP KIF18A KIF18B KIF23 KIF2C KIFC1 MAD2L1 MCM2 MCM6 MKI67 NCAPG NCAPG2 NCAPH NDC80 NEK2 NUF2 NUSAP1 OIP5 PLK1 PRC1 RAD54L SMC4 SPAG5 TOP2A TRIP13 TTK UHRF1 ZWINT	0
cell cycle checkpoint	25	AURKB BLM BRIP1 BUB1 BUB1B CCNB1 CDC25C CDC45 CLSPN DNA2 DTL E2F8 GEN1 GTSE1 KNTC1 MAD2L1 NDC80 ORC1 PLK1 TICRR TOP2A TRIP13 TTK ZWILCH ZWINT	0
mitotic sister chromatid segregation	30	AURKB BUB1 BUB1B CCNB1 CDCA5 CDCA8 CENPE CEP55 DLGAP5 ESPL1 GEN1 KIF18A KIF18B KIF23 KIF2C KIFC1 MAD2L1 NCAPG NCAPH NDC80 NEK2 NUF2 NUSAP1 PLK1 PRC1 SMC4 SPAG5 TRIP13 TTK ZWINT	0
cell cycle process	73	ANLN AURKB BLM BRIP1 BUB1 BUB1B CCNA2 CCNB1 CCNB2 CDC25A CDC25C CDC45 CDCA5 CDCA8 CDKN3 CENPA CENPE CEP55 CLSPN DBF4 DLGAP5 DNA2 DTL E2F8 ESCO2 ESPL1 FAM83D FAP FOXM1 GEN1 GINS1 GPSM2 GTSE1 HMMR IQGAP3 KIF11 KIF18A KIF18B KIF20A KIF23 KIF2C KIFC1 KNTC1 MAD2L1 MCM10 MCM2 MCM6 MKI67 NCAPG NCAPH NDC80 NEK2 NUF2 NUSAP1 ORC1 ORC6 PLK1 PLK4 PRC1 PRR11 RRM2 SMC4 SPAG5 SPC25 TICRR TOP2A TPX2 TRIP13 TTK UBD UBE2C ZWILCH ZWINT	0
mitotic cell cycle checkpoint	21	AURKB BLM BUB1 BUB1B CCNB1 CDC25C CLSPN E2F8 GEN1 GTSE1 KNTC1 MAD2L1 NDC80 ORC1 PLK1 TICRR TOP2A TRIP13 TTK ZWILCH ZWINT	0

regulation of chromosome segregation	18	AURKB BUB1 BUB1B CCNB1 CDCA5 CENPE DLGAP5 ESPL1 GEN1 KIF2C MAD2L1 MKI67 NDC80 NEK2 PLK1 SPAG5 TRIP13 TTK	0.0005
regulation of nuclear division	24	ANLN AURKB BUB1 BUB1B CCNA2 CCNB1 CCNB2 CDC25C CDCA5 CENPE DLGAP5 ESPL1 GEN1 KIF11 KNTC1 MAD2L1 MKI67 NDC80 NEK2 NUSAP1 PLK1 TRIP13 TTK UBE2C	0
regulation of mitotic cell cycle	42	ANLN AURKB BLM BUB1 BUB1B CCNA2 CCNB1 CCNB2 CDC25A CDC25C CDC45 CDCA5 CENPE CLSPN DLGAP5 DTL E2F8 ESPL1 FAP GEN1 GPSM2 GTSE1 HMMR KIF11 KNTC1 MAD2L1 MKI67 NDC80 NEK2 NUSAP1 ORC1 PLK1 PLK4 TICRR TOP2A TPX2 TRIP13 TTK UBD UBE2C ZWILCH ZWINT	0
organelle fission	43	ANLN AURKB BLM BRIP1 BUB1 BUB1B CCNA2 CCNB1 CCNB2 CDC25C CDCA5 CDCA8 CENPE CEP55 DLGAP5 ESPL1 GEN1 KIF11 KIF18A KIF18B KIF23 KIF2C KIFC1 KNTC1 MAD2L1 MKI67 MTFR2 NCAPG NCAPH NDC80 NEK2 NUF2 NUSAP1 PLK1 PRC1 SMC4 SPAG5 TOP2A TPX2 TRIP13 TTK UBE2C ZWINT	0.0059
nuclear division	42	ANLN AURKB BLM BRIP1 BUB1 BUB1B CCNA2 CCNB1 CCNB2 CDC25C CDCA5 CDCA8 CENPE CEP55 DLGAP5 ESPL1 GEN1 KIF11 KIF18A KIF18B KIF23 KIF2C KIFC1 KNTC1 MAD2L1 MKI67 NCAPG NCAPH NDC80 NEK2 NUF2 NUSAP1 PLK1 PRC1 SMC4 SPAG5 TOP2A TPX2 TRIP13 TTK UBE2C ZWINT	0.0374
mitotic cell cycle process	68	ANLN AURKB BLM BUB1 BUB1B CCNA2 CCNB1 CCNB2 CDC25A CDC25C CDC45 CDCA5 CDCA8 CDKN3 CENPA CENPE CEP55 CLSPN DBF4 DLGAP5 DNA2 DTL E2F8 ESPL1 FOXM1 GEN1 GINS1 GPSM2 GTSE1 HMMR IQGAP3 KIF11 KIF18A KIF18B KIF20A KIF23 KIF2C KIFC1 KNTC1 MAD2L1 MCM10 MCM2 MCM6 MKI67 NCAPG NCAPH NDC80 NEK2 NUF2 NUSAP1 ORC1 ORC6 PLK1 PLK4 PRC1 RRM2 SMC4 SPAG5 SPC25 TICRR TOP2A TPX2 TRIP13 TTK UBD UBE2C ZWILCH	0

mitotic cell cycle	73	ANLN AURKB BLM BUB1 BUB1B CCNA2 CCNB1 CCNB2 CDC25A CDC25C CDC45 CDCA5 CDCA8 CDKN3 CENPA CENPE CEP55 CLSPN DBF4 DLGAP5 DNA2 DTL E2F8 ESPL1 FAP FOXM1 GEN1 GINS1 GPSM2 GTSE1 HMMR IQGAP3 KIF11 KIF15 KIF18A KIF18B KIF20A KIF23 KIF2C KIFC1 KNTC1 MAD2L1 MCM10 MCM2 MCM6 MKI67 NCAPG NCAPH NDC80 NEK2 NUF2 NUSAP1 ORC1 ORC6 PLK1 PLK4 PRC1 RRM2 SKA1 SKA3 SMC4 SPAG5 SPC25 TICRR TOP2A TPX2 TRIP13 TTK UBD UBE2C WDHD1 ZWILCH ZWINT	0
cell cycle	85	ANLN AURKB BLM BRIP1 BUB1 BUB1B CCNA2 CCNB1 CCNB2 CDC25A CDC25C CDC45 CDCA2 CDCA3 CDCA5 CDCA8 CDKN3 CENPA CENPE CEP55 CLSPN DBF4 DLGAP5 DNA2 DTL E2F8 ERCC6L ESCO2 ESPL1 FAM83D FAP FOXM1 GEN1 GINS1 GPSM2 GTSE1 HJURP HMMR IQGAP3 KIF11 KIF15 KIF18A KIF18B KIF20A KIF23 KIF2C KIFC1 KNTC1 MAD2L1 MCM10 MCM2 MCM6 MKI67 NCAPG NCAPG2 NCAPH NDC80 NEK2 NUF2 NUSAP1 OIP5 ORC1 ORC6 PLK1 PLK4 PRC1 PRR11 RAD54L RRM2 SKA1 SKA3 SMC4 SPAG5 SPC25 TICRR TOP2A TPX2 TRIP13 TTK UBD UBE2C UHRF1 WDHD1 ZWILCH ZWINT	0.0012
chromosome segregation	42	AURKB BLM BRIP1 BUB1 BUB1B CCNB1 CDCA5 CDCA8 CENPE CENPN CEP55 DLGAP5 ESCO2 ESPL1 FAM83D GEN1 HJURP KIF18A KIF18B KIF23 KIF2C KIFC1 MAD2L1 MKI67 NCAPG NCAPH NDC80 NEK2 NUF2 NUSAP1 OIP5 PLK1 PRC1 SKA1 SKA3 SMC4 SPAG5 SPC25 TOP2A TRIP13 TTK ZWINT	0.0012
mitotic nuclear division	39	ANLN AURKB BUB1 BUB1B CCNA2 CCNB1 CCNB2 CDC25C CDCA5 CDCA8 CENPE CEP55 DLGAP5 ESPL1 GEN1 KIF11 KIF18A KIF18B KIF23 KIF2C KIFC1 KNTC1 MAD2L1 MKI67 NCAPG NCAPH NDC80 NEK2 NUF2 NUSAP1 PLK1 PRC1 SMC4 SPAG5 TPX2 TRIP13 TTK UBE2C ZWINT	0.0012
cell division	52	ANLN AURKB BLM BRIP1 BUB1 BUB1B CCNA2 CCNB1 CCNB2 CDC25A CDC25C CDCA2 CDCA3 CDCA5 CDCA8 CENPA CENPE CEP55 E2F8 ERCC6L ESPL1 FAM83D GPSM2 KIF11 KIF18B KIF20A KIF23 KIF2C KIFC1 KNTC1 MAD2L1 NCAPG NCAPG2 NCAPH NDC80 NEK2 NUF2 NUSAP1 OIP5 ORC6 PLK1 PRC1 SKA1 SKA3 SMC4 SPAG5 SPC25 TOP2A TPX2 UBE2C ZWILCH ZWINT	0.0012
neural structure formation in CNS (R^2_0.8831, adjR^2_0.8688, p-value_0.0095) at N2			

related non-neural pathways	gene number	gene name	p-value
sister chromatid segregation	32	AURKB BUB1 BUB1B CCNB1 CDCA5 CDCA8 CENPE CEP55 DLGAP5 ESCO2 ESPL1 GEN1 KIF18A KIF18B KIF23 KIF2C KIFC1 MAD2L1 NCAPG NCAPH NDC80 NEK2 NUF2 NUSAP1 PLK1 PRC1 SMC4 SPAG5 TOP2A TRIP13 TTK ZWINT	0
mitotic cell cycle	73	ANLN AURKB BLM BUB1 BUB1B CCNA2 CCNB1 CCNB2 CDC25A CDC25C CDC45 CDCA5 CDCA8 CDKN3 CENPA CENPE CEP55 CLSPN DBF4 DLGAP5 DNA2 DTL E2F8 ESPL1 FAP FOXM1 GEN1 GINS1 GPSM2 GTSE1 HMMR IQGAP3 KIF11 KIF15 KIF18A KIF18B KIF20A KIF23 KIF2C KIFC1 KNTC1 MAD2L1 MCM10 MCM2 MCM6 MKI67 NCAPG NCAPH NDC80 NEK2 NUF2 NUSAP1 ORC1 ORC6 PLK1 PLK4 PRC1 RRM2 SKA1 SKA3 SMC4 SPAG5 SPC25 TICRR TOP2A TPX2 TRIP13 TTK UBD UBE2C WDHD1 ZWILCH ZWINT	0
mitotic cell cycle process	68	ANLN AURKB BLM BUB1 BUB1B CCNA2 CCNB1 CCNB2 CDC25A CDC25C CDC45 CDCA5 CDCA8 CDKN3 CENPA CENPE CEP55 CLSPN DBF4 DLGAP5 DNA2 DTL E2F8 ESPL1 FOXM1 GEN1 GINS1 GPSM2 GTSE1 HMMR IQGAP3 KIF11 KIF18A KIF18B KIF20A KIF23 KIF2C KIFC1 KNTC1 MAD2L1 MCM10 MCM2 MCM6 MKI67 NCAPG NCAPH NDC80 NEK2 NUF2 NUSAP1 ORC1 ORC6 PLK1 PLK4 PRC1 RRM2 SMC4 SPAG5 SPC25 TICRR TOP2A TPX2 TRIP13 TTK UBD UBE2C ZWILCH ZWINT	0
mitotic nuclear division	39	ANLN AURKB BUB1 BUB1B CCNA2 CCNB1 CCNB2 CDC25C CDCA5 CDCA8 CENPE CEP55 DLGAP5 ESPL1 GEN1 KIF11 KIF18A KIF18B KIF23 KIF2C KIFC1 KNTC1 MAD2L1 MKI67 NCAPG NCAPH NDC80 NEK2 NUF2 NUSAP1 PLK1 PRC1 SMC4 SPAG5 TPX2 TRIP13 TTK UBE2C ZWINT	0.0098

cell cycle	85	ANLN AURKB BLM BRIP1 BUB1 BUB1B CCNA2 CCNB1 CCNB2 CDC25A CDC25C CDC45 CDCA2 CDCA3 CDCA5 CDCA8 CDKN3 CENPA CENPE CEP55 CLSPN DBF4 DLGAP5 DNA2 DTL E2F8 ERCC6L ESCO2 ESPL1 FAM83D FAP FOXM1 GEN1 GINS1 GPSM2 GTSE1 HJURP HMMR IQGAP3 KIF11 KIF15 KIF18A KIF18B KIF20A KIF23 KIF2C KIFC1 KNTC1 MAD2L1 MCM10 MCM2 MCM6 MKI67 NCAPG NCAPG2 NCAPH NDC80 NEK2 NUF2 NUSAP1 OIP5 ORC1 ORC6 PLK1 PLK4 PRC1 PRR11 RAD54L RRM2 SKA1 SKA3 SMC4 SPAG5 SPC25 TICRR TOP2A TPX2 TRIP13 TTK UBD UBE2C UHRF1 WDHD1 ZWILCH ZWINT	0.0138
neuron differentiation (R^2_0.8908, adjR^2_0.8688, p-value_0.0038) at N2			
related non-neural pathways	gene number	gene name	p-value
regulation of cell cycle process	46	ANLN AURKB BLM BUB1 BUB1B CCNA2 CCNB1 CCNB2 CDC25A CDC25C CDC45 CDCA5 CENPE CLSPN DLGAP5 DTL E2F8 ESPL1 FAM83D FAP FOXM1 GEN1 GPSM2 GTSE1 HMMR KIF11 KIF20A KIF23 KNTC1 MAD2L1 MKI67 NDC80 NEK2 NUSAP1 ORC1 PLK1 PLK4 PRC1 RRM2 SPAG5 TICRR TPX2 TRIP13 TTK UBD UBE2C	0
mitotic nuclear division	39	ANLN AURKB BUB1 BUB1B CCNA2 CCNB1 CCNB2 CDC25C CDCA5 CDCA8 CENPE CEP55 DLGAP5 ESPL1 GEN1 KIF11 KIF18A KIF18B KIF23 KIF2C KIFC1 KNTC1 MAD2L1 MKI67 NCAPG NCAPH NDC80 NEK2 NUF2 NUSAP1 PLK1 PRC1 SMC4 SPAG5 TPX2 TRIP13 TTK UBE2C ZWINT	0
chromosome organization	55	ASF1B ATAD2 AURKB BLM BRIP1 BUB1 BUB1B CCNA2 CCNB1 CDC45 CDCA5 CDCA8 CENPA CENPE CENPI CENPL CENPN CEP55 DLGAP5 DNA2 ESCO2 ESPL1 EXO1 FOXP3 GEN1 GINS1 HIST1H2BO HJURP KIF18A KIF18B KIF23 KIF2C KIFC1 MAD2L1 MCM2 MCM6 MKI67 NCAPG NCAPG2 NCAPH NDC80 NEK2 NUF2 NUSAP1 OIP5 PLK1 PRC1 RAD54L SMC4 SPAG5 TOP2A TRIP13 TTK UHRF1 ZWINT	0.0039
cell cycle phase transition	44	ANLN AURKB BLM BUB1 BUB1B CCNA2 CCNB1 CCNB2 CDC25A CDC25C CDC45 CDCA5 CDKN3 CENPE CLSPN DBF4 DLGAP5 DTL E2F8 ESPL1 FAM83D FOXM1 GEN1 GTSE1 HMMR IQGAP3 KNTC1 MAD2L1 MCM10 MCM2 MCM6 NDC80 NEK2 ORC1 ORC6 PLK1 PLK4 RRM2 TICRR TPX2 TRIP13 TTK UBD UBE2C	0

mitotic sister chromatid segregation	30	AURKB BUB1 BUB1B CCNB1 CDCA5 CDCA8 CENPE CEP55 DLGAP5 ESPL1 GEN1 KIF18A KIF18B KIF23 KIF2C KIFC1 MAD2L1 NCAPG NCAPH NDC80 NEK2 NUF2 NUSAP1 PLK1 PRC1 SMC4 SPAG5 TRIP13 TTK ZWINT	0
mitotic cell cycle process	68	ANLN AURKB BLM BUB1 BUB1B CCNA2 CCNB1 CCNB2 CDC25A CDC25C CDC45 CDCA5 CDCA8 CDKN3 CENPA CENPE CEP55 CLSPN DBF4 DLGAP5 DNA2 DTL E2F8 ESPL1 FOXM1 GEN1 GINS1 GPSM2 GTSE1 HMMR IQGAP3 KIF11 KIF18A KIF18B KIF20A KIF23 KIF2C KIFC1 KNTC1 MAD2L1 MCM10 MCM2 MCM6 MKI67 NCAPG NCAPH NDC80 NEK2 NUF2 NUSAP1 ORC1 ORC6 PLK1 PLK4 PRC1 RRM2 SMC4 SPAG5 SPC25 TICRR TOP2A TPX2 TRIP13 TTK UBD UBE2C ZWILCH ZWINT	0
chromosome segregation	42	AURKB BLM BRIP1 BUB1 BUB1B CCNB1 CDCA5 CDCA8 CENPE CENPN CEP55 DLGAP5 ESCO2 ESPL1 FAM83D GEN1 HJURP KIF18A KIF18B KIF23 KIF2C KIFC1 MAD2L1 MKI67 NCAPG NCAPH NDC80 NEK2 NUF2 NUSAP1 OIP5 PLK1 PRC1 SKA1 SKA3 SMC4 SPAG5 SPC25 TOP2A TRIP13 TTK ZWINT	0
nuclear division	42	ANLN AURKB BLM BRIP1 BUB1 BUB1B CCNA2 CCNB1 CCNB2 CDC25C CDCA5 CDCA8 CENPE CEP55 DLGAP5 ESPL1 GEN1 KIF11 KIF18A KIF18B KIF23 KIF2C KIFC1 KNTC1 MAD2L1 MKI67 NCAPG NCAPH NDC80 NEK2 NUF2 NUSAP1 PLK1 PRC1 SMC4 SPAG5 TOP2A TPX2 TRIP13 TTK UBE2C ZWINT	0.0275
cell cycle process	73	ANLN AURKB BLM BRIP1 BUB1 BUB1B CCNA2 CCNB1 CCNB2 CDC25A CDC25C CDC45 CDCA5 CDCA8 CDKN3 CENPA CENPE CEP55 CLSPN DBF4 DLGAP5 DNA2 DTL E2F8 ESCO2 ESPL1 FAM83D FAP FOXM1 GEN1 GINS1 GPSM2 GTSE1 HMMR IQGAP3 KIF11 KIF18A KIF18B KIF20A KIF23 KIF2C KIFC1 KNTC1 MAD2L1 MCM10 MCM2 MCM6 MKI67 NCAPG NCAPH NDC80 NEK2 NUF2 NUSAP1 ORC1 ORC6 PLK1 PLK4 PRC1 PRR11 RRM2 SMC4 SPAG5 SPC25 TICRR TOP2A TPX2 TRIP13 TTK UBD UBE2C ZWILCH ZWINT	0.0112
nuclear chromosome segregation	35	AURKB BLM BRIP1 BUB1 BUB1B CCNB1 CDCA5 CDCA8 CENPE CEP55 DLGAP5 ESCO2 ESPL1 FAM83D GEN1 KIF18A KIF18B KIF23 KIF2C KIFC1 MAD2L1 NCAPG NCAPH NDC80 NEK2 NUF2 NUSAP1 PLK1 PRC1 SMC4 SPAG5 TOP2A TRIP13 TTK ZWINT	0.0155

mitotic cell cycle	73	ANLN AURKB BLM BUB1 BUB1B CCNA2 CCNB1 CCNB2 CDC25A CDC25C CDC45 CDCA5 CDCA8 CDKN3 CENPA CENPE CEP55 CLSPN DBF4 DLGAP5 DNA2 DTL E2F8 ESPL1 FAP FOXM1 GEN1 GINS1 GPSM2 GTSE1 HMMR IQGAP3 KIF11 KIF15 KIF18A KIF18B KIF20A KIF23 KIF2C KIFC1 KNTC1 MAD2L1 MCM10 MCM2 MCM6 MKI67 NCAPG NCAPH NDC80 NEK2 NUF2 NUSAP1 ORC1 ORC6 PLK1 PLK4 PRC1 RRM2 SKA1 SKA3 SMC4 SPAG5 SPC25 TICRR TOP2A TPX2 TRIP13 TTK UBD UBE2C WDHD1 ZWILCH ZWINT	0.0015
regulation of mitotic nuclear division	24	ANLN AURKB BUB1 BUB1B CCNA2 CCNB1 CCNB2 CDC25C CDCA5 CENPE DLGAP5 ESPL1 GEN1 KIF11 KNTC1 MAD2L1 MKI67 NDC80 NEK2 NUSAP1 PLK1 TRIP13 TTK UBE2C	0.0003
development of glial (R^2_0.7995, adjR^2_0.7515, p-value_0.0001) at N2			
related non-neural pathways	gene number	gene name	p-value
cell division	52	ANLN AURKB BLM BRIP1 BUB1 BUB1B CCNA2 CCNB1 CCNB2 CDC25A CDC25C CDCA2 CDCA3 CDCA5 CDCA8 CENPA CENPE CEP55 E2F8 ERCC6L ESPL1 FAM83D GPSM2 KIF11 KIF18B KIF20A KIF23 KIF2C KIFC1 KNTC1 MAD2L1 NCAPG NCAPG2 NCAPH NDC80 NEK2 NUF2 NUSAP1 OIP5 ORC6 PLK1 PRC1 SKA1 SKA3 SMC4 SPAG5 SPC25 TOP2A TPX2 UBE2C ZWILCH ZWINT	0
cell cycle	85	ANLN AURKB BLM BRIP1 BUB1 BUB1B CCNA2 CCNB1 CCNB2 CDC25A CDC25C CDC45 CDCA2 CDCA3 CDCA5 CDCA8 CDKN3 CENPA CENPE CEP55 CLSPN DBF4 DLGAP5 DNA2 DTL E2F8 ERCC6L ESCO2 ESPL1 FAM83D FAP FOXM1 GEN1 GINS1 GPSM2 GTSE1 HJURP HMMR IQGAP3 KIF11 KIF15 KIF18A KIF18B KIF20A KIF23 KIF2C KIFC1 KNTC1 MAD2L1 MCM10 MCM2 MCM6 MKI67 NCAPG NCAPG2 NCAPH NDC80 NEK2 NUF2 NUSAP1 OIP5 ORC1 ORC6 PLK1 PLK4 PRC1 PRR11 RAD54L RRM2 SKA1 SKA3 SMC4 SPAG5 SPC25 TICRR TOP2A TPX2 TRIP13 TTK UBD UBE2C UHRF1 WDHD1 ZWILCH ZWINT	0

cell cycle process	73	ANLN AURKB BLM BRIP1 BUB1 BUB1B CCNA2 CCNB1 CCNB2 CDC25A CDC25C CDC45 CDCA5 CDCA8 CDKN3 CENPA CENPE CEP55 CLSPN DBF4 DLGAP5 DNA2 DTL E2F8 ESCO2 ESPL1 FAM83D FAP FOXM1 GEN1 GINS1 GPSM2 GTSE1 HMMR IQGAP3 KIF11 KIF18A KIF18B KIF20A KIF23 KIF2C KIFC1 KNTC1 MAD2L1 MCM10 MCM2 MCM6 MKI67 NCAPG NCAPH NDC80 NEK2 NUF2 NUSAP1 ORC1 ORC6 PLK1 PLK4 PRC1 PRR11 RRM2 SMC4 SPAG5 SPC25 TICRR TOP2A TPX2 TRIP13 TTK UBD UBE2C ZWILCH ZWINT	0
regulation of mitotic nuclear division	24	ANLN AURKB BUB1 BUB1B CCNA2 CCNB1 CCNB2 CDC25C CDCA5 CENPE DLGAP5 ESPL1 GEN1 KIF11 KNTC1 MAD2L1 MKI67 NDC80 NEK2 NUSAP1 PLK1 TRIP13 TTK UBE2C	0
mitotic nuclear division	39	ANLN AURKB BUB1 BUB1B CCNA2 CCNB1 CCNB2 CDC25C CDCA5 CDCA8 CENPE CEP55 DLGAP5 ESPL1 GEN1 KIF11 KIF18A KIF18B KIF23 KIF2C KIFC1 KNTC1 MAD2L1 MKI67 NCAPG NCAPH NDC80 NEK2 NUF2 NUSAP1 PLK1 PRC1 SMC4 SPAG5 TPX2 TRIP13 TTK UBE2C ZWINT	0
nuclear chromosome segregation	35	AURKB BLM BRIP1 BUB1 BUB1B CCNB1 CDCA5 CDCA8 CENPE CEP55 DLGAP5 ESCO2 ESPL1 FAM83D GEN1 KIF18A KIF18B KIF23 KIF2C KIFC1 MAD2L1 NCAPG NCAPH NDC80 NEK2 NUF2 NUSAP1 PLK1 PRC1 SMC4 SPAG5 TOP2A TRIP13 TTK ZWINT	0
mitotic sister chromatid segregation	30	AURKB BUB1 BUB1B CCNB1 CDCA5 CDCA8 CENPE CEP55 DLGAP5 ESPL1 GEN1 KIF18A KIF18B KIF23 KIF2C KIFC1 MAD2L1 NCAPG NCAPH NDC80 NEK2 NUF2 NUSAP1 PLK1 PRC1 SMC4 SPAG5 TRIP13 TTK ZWINT	0.025
synapse formation (R^2_0.8312, adjR^2_0.8138, p-value_0.0015) at N3			
related non-neural pathways	gene number	gene name	p-value
chromosome segregation	44	AURKB BLM BRIP1 BUB1 BUB1B CCNB1 CCNE2 CDC6 CDCA5 CDCA8 CDT1 CENPE CEP55 DLGAP5 ESCO2 ESPL1 FAM83D HJURP KIF18A KIF18B KIF23 KIF2C KIFC1 KNSTRN MAD2L1 MKI67 NCAPG NCAPH NDC80 NEK2 NUF2 NUSAP1 OIP5 PLK1 PRC1 PTTG1 SKA1 SKA3 SPAG5 SPC25 TOP2A TRIP13 TTK ZWINT	0

mitotic nuclear division	42	ANLN AURKB BUB1 BUB1B CCNA2 CCNB1 CCNB2 CCNE2 CDC25C CDC6 CDCA5 CDCA8 CDT1 CENPE CEP55 DLGAP5 ESPL1 KIF11 KIF18A KIF18B KIF23 KIF2C KIFC1 KNSTRN MAD2L1 MKI67 NCAPG NCAPH NDC80 NEK2 NUF2 NUSAP1 PLK1 PRC1 PTTG1 SPAG5 TPX2 TRIP13 TTK UBE2C UBE2S ZWINT	0

neuron projection and apoptosis (R^2_0.9111, adjR^2_0.8581, p-value_0) at N3

related non-neural pathways	gene number	gene name	p-value
regulation of chromosome segregation	21	AURKB BUB1 BUB1B CCNB1 CDC6 CDCA5 CDT1 CENPE DLGAP5 ESPL1 KIF2C KNSTRN MAD2L1 MKI67 NDC80 NEK2 PLK1 PTTG1 SPAG5 TRIP13 TTK	0
chromosome separation	18	AURKB BLM BUB1 BUB1B CCNB1 CDC6 CDT1 CENPE DLGAP5 ESPL1 MAD2L1 NCAPH NDC80 PLK1 PTTG1 TOP2A TRIP13 TTK	0
regulation of cell cycle process	51	ANLN AURKB BLM BUB1 BUB1B CCNA2 CCNB1 CCNB2 CCNE2 CDC25A CDC25C CDC45 CDC6 CDCA5 CDT1 CENPE CLSPN DLGAP5 DONSON DTL E2F7 E2F8 ESPL1 FAM83D FAP FOXM1 GTSE1 HMMR KIF11 KIF20A KIF23 KNSTRN MAD2L1 MKI67 NDC80 NEK2 NUSAP1 ORC1 PLK1 PLK4 PRC1 PTTG1 RAB11FIP3 RAD51 RRM2 SPAG5 TICRR TPX2 TRIP13 TTK UBE2C	0.0052
organelle fission	49	ANLN AURKB BLM BRIP1 BUB1 BUB1B CCNA2 CCNB1 CCNB2 CCNE2 CDC25C CDC6 CDCA5 CDCA8 CDT1 CENPE CEP55 CKS2 DLGAP5 ESPL1 KIF11 KIF18A KIF18B KIF23 KIF2C KIFC1 KNSTRN MAD2L1 MKI67 MND1 MTFR2 NCAPG NCAPH NDC80 NEK2 NUF2 NUSAP1 PLK1 PRC1 PTTG1 RAD51 SPAG5 TOP2A TPX2 TRIP13 TTK UBE2C UBE2S ZWINT	0.044
nuclear chromosome segregation	38	AURKB BLM BRIP1 BUB1 BUB1B CCNB1 CCNE2 CDC6 CDCA5 CDCA8 CDT1 CENPE CEP55 DLGAP5 ESCO2 ESPL1 FAM83D KIF18A KIF18B KIF23 KIF2C KIFC1 KNSTRN MAD2L1 NCAPG NCAPH NDC80 NEK2 NUF2 NUSAP1 PLK1 PRC1 PTTG1 SPAG5 TOP2A TRIP13 TTK ZWINT	0.0192

mitotic cell cycle process	71	ANLN AURKB BLM BUB1 BUB1B CCNA2 CCNB1 CCNB2 CCNE2 CDC25A CDC25C CDC45 CDC6 CDCA5 CDCA8 CDKN3 CDT1 CENPA CENPE CEP55 CKS2 CLSPN DLGAP5 DNA2 DONSON DTL E2F7 E2F8 ESPL1 FOXM1 GINS1 GTSE1 HMMR IQGAP3 KIF11 KIF18A KIF18B KIF20A KIF23 KIF2C KIFC1 KNSTRN MAD2L1 MCM10 MCM2 MKI67 NCAPG NCAPH NDC80 NEK2 NUF2 NUSAP1 ORC1 ORC6 PLK1 PLK4 PRC1 PTTG1 RAD51 RRM2 SPAG5 SPC25 TICRR TOP2A TPX2 TRIP13 TTK TYMS UBE2C UBE2S ZWINT	0.0279
DNA metabolic process	50	AUNIP AURKB BLM BRIP1 CCNA2 CCNE2 CDC45 CDC6 CDCA5 CDT1 CLSPN DNA2 DONSON DTL E2F7 E2F8 ESCO2 EXO1 EXOSC4 FANCI FOXM1 FOXP3 GINS1 GZMA HELLS MCM10 MCM2 MND1 MPG NEIL3 NEK2 NUDT16L1 ORC1 ORC6 PARPBP POLQ PTTG1 RAD51 RAD51AP1 RAD54L RRM2 TICRR TIMELESS TK1 TNFSF4 TOP2A TRIP13 TYMS UBE2T UHRF1	0.0254
neuron differentiation (R^2_0.8692, adjR^2_0.8217, p-value_0.0299) at N3			
related non-neural pathways	gene number	gene name	p-value
mitotic cell cycle phase transition	46	ANLN AURKB BLM BUB1 BUB1B CCNA2 CCNB1 CCNB2 CCNE2 CDC25A CDC25C CDC45 CDC6 CDCA5 CDKN3 CDT1 CENPE CKS2 CLSPN DLGAP5 DONSON DTL E2F7 E2F8 ESPL1 FOXM1 GTSE1 HMMR IQGAP3 MAD2L1 MCM10 MCM2 NDC80 NEK2 ORC1 ORC6 PLK1 PLK4 RRM2 TICRR TPX2 TRIP13 TTK TYMS UBE2C UBE2S	0
regulation of mitotic cell cycle	44	ANLN AURKB BLM BUB1 BUB1B CCNA2 CCNB1 CCNB2 CCNE2 CDC25A CDC25C CDC45 CDC6 CDCA5 CDT1 CENPE CKS2 CLSPN DLGAP5 DONSON DTL E2F7 E2F8 ESPL1 FAP GTSE1 HMMR KIF11 MAD2L1 MKI67 NDC80 NEK2 NUSAP1 ORC1 PLK1 PLK4 PTTG1 TICRR TOP2A TPX2 TRIP13 TTK UBE2C ZWINT	0
organelle fission	49	ANLN AURKB BLM BRIP1 BUB1 BUB1B CCNA2 CCNB1 CCNB2 CCNE2 CDC25C CDC6 CDCA5 CDCA8 CDT1 CENPE CEP55 CKS2 DLGAP5 ESPL1 KIF11 KIF18A KIF18B KIF23 KIF2C KIFC1 KNSTRN MAD2L1 MKI67 MND1 MTFR2 NCAPG NCAPH NDC80 NEK2 NUF2 NUSAP1 PLK1 PRC1 PTTG1 RAD51 SPAG5 TOP2A TPX2 TRIP13 TTK UBE2C UBE2S ZWINT	0.0047
development of glial (R^2_0.7721, adjR^2_0.7449, p-value_0.0157) at N3			
related non-neural pathways	gene number	gene name	p-value

mitotic nuclear division	42	ANLN AURKB BUB1 BUB1B CCNA2 CCNB1 CCNB2 CCNE2 CDC25C CDC6 CDCA5 CDCA8 CDT1 CENPE CEP55 DLGAP5 ESPL1 KIF11 KIF18A KIF18B KIF23 KIF2C KIFC1 KNSTRN MAD2L1 MKI67 NCAPG NCAPH NDC80 NEK2 NUF2 NUSAP1 PLK1 PRC1 PTTG1 SPAG5 TPX2 TRIP13 TTK UBE2C UBE2S ZWINT	0
cell cycle process	80	ANLN AUNIP AURKB BLM BRIP1 BUB1 BUB1B CCNA2 CCNB1 CCNB2 CCNE2 CDC25A CDC25C CDC45 CDC6 CDCA5 CDCA8 CDKN3 CDT1 CENPA CENPE CEP55 CKS2 CLSPN DLGAP5 DNA2 DONSON DTL E2F7 E2F8 ESCO2 ESPL1 FAM83D FAP FOXM1 GINS1 GTSE1 HMMR IQGAP3 KIF11 KIF18A KIF18B KIF20A KIF23 KIF2C KIFC1 KNSTRN MAD2L1 MCM10 MCM2 MKI67 MND1 NCAPG NCAPH NDC80 NEK2 NUF2 NUSAP1 ORC1 ORC6 PLK1 PLK4 PRC1 PRR11 PTTG1 RAB11FIP3 RAD51 RRM2 SPAG5 SPC25 TICRR TIMELESS TOP2A TPX2 TRIP13 TTK TYMS UBE2C UBE2S ZWINT	0
nuclear division	48	ANLN AURKB BLM BRIP1 BUB1 BUB1B CCNA2 CCNB1 CCNB2 CCNE2 CDC25C CDC6 CDCA5 CDCA8 CDT1 CENPE CEP55 CKS2 DLGAP5 ESPL1 KIF11 KIF18A KIF18B KIF23 KIF2C KIFC1 KNSTRN MAD2L1 MKI67 MND1 NCAPG NCAPH NDC80 NEK2 NUF2 NUSAP1 PLK1 PRC1 PTTG1 RAD51 SPAG5 TOP2A TPX2 TRIP13 TTK UBE2C UBE2S ZWINT	0.0046

Supplementary Table S6. p-value and the difference of R^2 of TNBC

	innate immunity			adaptive immunity			cell cycle regulation			DNA replication		
		p-value	difference of R^2		p-value	difference of R^2		p-value	difference of R^2		p-value	difference of R^2
T1N0	telencephalon development	9.53E-04	0.111361	telencephalon development	1.50E-03	0.094457	telencephalon development	6.69E-06	0.011543	regulation of synaptic transmission, glutamatergic	8.11E-04	0.01294
	pallium development	3.37E-03	0.094916	pallium development	9.76E-04	0.114813				telencephalon development	3.28E-08	0.010012
T2N0	central nervous system neuron differentiation	6.94E-06	0.027513	pallium development	1.45E-06	0.049302	negative regulation of synaptic transmission	1.30E-05	0.062065			
	telencephalon development	5.11E-09	0.01474	neural precursor cell proliferation	6.63E-07	0.018365	cerebral cortex development	2.13E-03	0.015198			
	neural tube closure	5.36E-03	0.019144									
T3N0	associative learning	5.64E-08	0.090845	associative learning	4.65E-10	0.088971	forebrain neuron differentiation	2.17E-12	0.131189	forebrain neuron differentiation	2.44E-10	0.176115
				cerebral cortex development	5.39E-03	0.011326	neuroblast proliferation	3.49E-05	0.040174	neuroblast proliferation	1.21E-02	0.020106
N1	regulation of dendrite morphogenesis	4.22E-08	0.042076	neuron death	2.93E-09	0.042626	regulation of neurotransmitter secretion	4.83E-13	0.033768	primary neural tube formation	2.22E-16	0.020003
				neural crest cell differentiation	3.43E-03	0.011398	regulation of dendrite morphogenesis	9.32E-04	0.010468			
N3	cerebral cortex development	3.33E-04	0.049779									

Supplementary Table S7. p-value and the difference of R^2 of NTNBC

	**innate immunity**			**adaptive immunity**			**cell cycle regulation**			**DNA replication**		
	neural pathway	p-value	difference of R^2	neural pathway	p-value	difference of R^2	neural pathway	p-value	difference of R^2	neural pathway	p-value	difference of R^2
T1N0	axonal transport	0.00E+00	0.013669	brain development	0.00E+00	0.010132						
	regulation of neuron apoptotic process	6.73E-12	0.025327	negative regulation of synaptic transmission	2.88E-08	0.017649						
	neuron fate commitment	4.54E-07	0.023168	neural tube formation	5.32E-07	0.022061						
T2N0	central nervous system neuron differentiation	0.00E+00	0.02722	forebrain development	0.00E+00	0.034142	regulation of nervous system development	0	0.044719	neuron apoptotic process	5.08E-11	0.011426
	pallium development	5.55E-10	0.02506	central nervous system development	7.40E-07	0.016227				cerebral cortex development	0.00E+00	0.020613
	neuron death	5.06E-13	0.032842	neuron death	1.74E-03	0.012892						
				central nervous system neuron differentiation	6.07E-03	0.01206						
T3N0							gliogenesis	6.93E-07	0.022098	primary neural tube formation	3.03E-02	0.014199
							regulation of gliogenesis	5.45E-08	0.018617			
N1	gliogenesis	0.00E+00	0.028433	neuron fate commitment	0.00E+00	0.038106	pallium development	0	0.034039	spinal cord development	0.00E+00	0.023514
	cerebral cortex development	7.27E-12	0.02432	central nervous system development	2.61E-13	0.020147						
	pallium development	5.70E-08	0.054379	gliogenesis	0.00E+00	0.061907						
	neural tube closure	2.22E-16	0.081129	neural precursor cell proliferation	2.89E-09	0.038622						
N2	sensory perception of sound	2.95E-03	0.019787	primary neural tube formation	0.00E+00	0.0352						
	pallium development	2.49E-04	0.019108	central nervous system neuron differentiation	3.11E-08	0.075032						
	neuron fate commitment	1.38E-02	0.011607									
N3							sensory perception of sound	1.14E-06	0.012496	neural tube formation	3.27E-06	0.02623
							neural tube closure	3.40E-06	0.010591	pallium development	1.26E-04	0.013745
							regulation of neural precursor cell proliferation	3.58E-07	0.014575			
							sensory perception of mechanical stimulus	2.62E-05	0.010146			

Supplementary Table S8. Sample size distributed by staging information in TNBC and NTNBC.

	T1N0	T2N0	T3N0	N1	N2	N3	normal
TNBC	19	47	10	25	12	6	11
NTNBC	90	221	47	118	57	29	52
